# Mechanistic Versatility at Ir(PSiP) Pincer Catalysts: Triflate Proton Shuttling from 2-Butyne to Diene and [3]Dendralene Motifs

**DOI:** 10.1021/acs.organomet.2c00375

**Published:** 2022-09-13

**Authors:** José L. Andrés, Elizabeth Suárez, Marta Martín, Eduardo Sola

**Affiliations:** Instituto de Síntesis Química y Catálisis Homogénea (ISQCH), CSIC − Universidad de Zaragoza, Facultad de Ciencias, E50009 Zaragoza, Spain

## Abstract

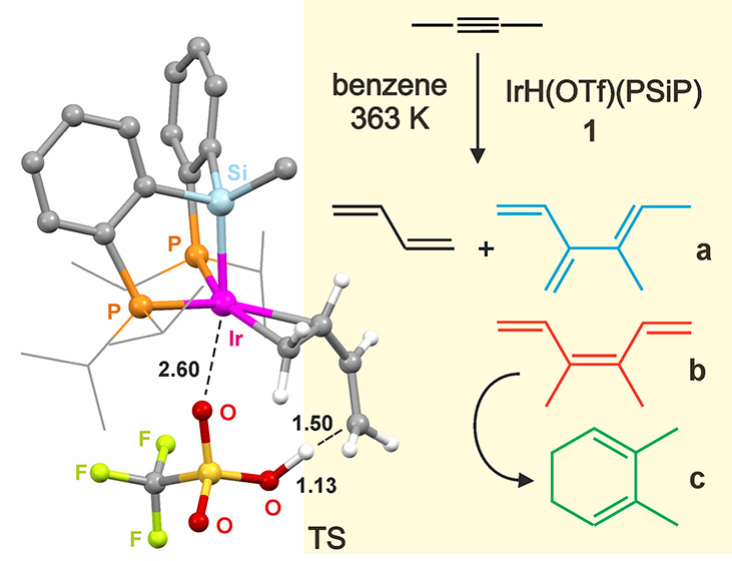

The five-coordinate hydrido complex [IrH(OTf)(PSiP)] (**1**) catalytically transforms 2-butyne into a mixture of its isomer 1,3-butadiene, and [3]dendralene and linear hexatriene dimerization products: (*E*)-4-methyl-3-methylene-1,4-hexadiene and (3*Z*)-3,4-dimethyl-1,3,5-hexatriene, respectively. Under the conditions of the catalytic reaction, benzene, and 363 K, the hexatriene further undergoes thermal electrocyclization into 2,3-dimethyl-1,3-cyclohexadiene. The reactions between **1** and the alkyne substrate allow isolation or nuclear magnetic resonance (NMR) observation of catalyst resting states and possible reaction intermediates, including complexes with the former PSiP pincer ligands disassembled into PSi and PC chelates, and species coordinating allyl or carbene fragments en route to products. The density functional theory (DFT) calculations guided by these experimental observations disclose competing mechanisms for C–H bond elaboration that move H atoms either classically, as hydrides, or as protons transported by the triflate. This latter role of triflate, previously recognized only for more basic anions such as carboxylates, is discussed to result from combining the unfavorable charge separation in the nonpolar solvent and the low electronic demand from the metal to the anion at coordination positions trans to silicon. Triflate deprotonation of methyl groups is key to release highly coordinating diene products from stable allyl intermediates, thus enabling catalytic cycling.

## Introduction

The di- and oligomerization of alkynes catalyzed by transition-metal complexes provide atom-economic access to a variety of structural motifs.^1^ Prevailing examples are Reppe-type [2 + 2 + 2] cyclotrimerizations to form arenes^2−5^ and, in the case of 1-alkynes, oxidative couplings to 1,3-diynes^6,7^ and dimerizations into 1-en-3-ynes or butatrienes:^8−10^ all of them of great synthetic utility if regioselective.^11,12^ Along with these classics, the chemical literature shows particular examples leading to other less-common structures. Among them, catalytic [2 + 2 + 2 + 2] cyclotetramerizations to cyclooctatetraenes^13^ or [2 + 2 + 1] cyclotrimerizations leading to fulvenes^14,15^ can occasionally compete with the formation of six-membered rings in Reppe-type transformations. Also, catalytic dimerization into butadienes^16,17^ or bis-allenes,^18^ tetramerization into bicyclic isobenzenes,^19^ and oligomerization to form linear-conjugated acyclic polyenes^20−22^ have been demonstrated in particular cases. In contrast, only a few stoichiometric alkyne-based syntheses have been reported toward dendralenes: the cross-conjugated versions of acyclic polyenes.^23,24^

**Scheme 1:**
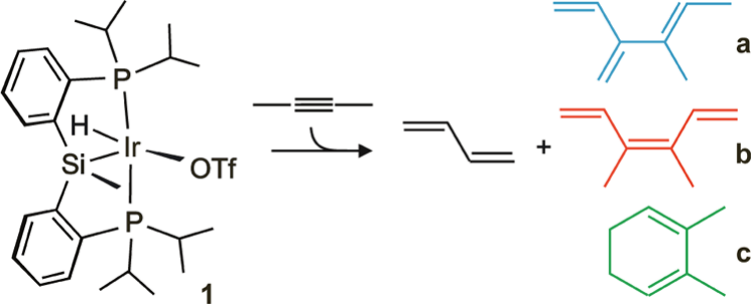
Catalytic Transformation of 2-Butyne

During our investigation of organometallic reactivity patterns in Ir(PSiP) pincer complexes,^25^ we observed that the five-coordinate hydride [IrH{κO-O_3_S(CF_3_)}{κ*P,P,Si*-SiMe(C_6_H_4_-2-*Pi*Pr_2_)_2_}] = ([IrH(OTf)(PSiP)], **1**)^26^ was capable of catalytically transforming 2-butyne into its more stable 1,3-butadiene isomer,^27^ also forming dimerization products **a–c** (Scheme 1). Aside from the isomerization into butadiene, which is exceptional for nonactivated alkynes,^28−31^ we found particularly appealing the generation of dendralene **a**, (*E*)-4-methyl-3-methylene-1,4-hexadiene, since current synthetic methods toward these challenging branched structures mainly rely on cross-coupling reactions of low atom economy.^32−37^ Aimed at identifying keys for this unprecedented catalytic outcome, this work scrutinizes the reactions between **1** and 2-butyne to conclude that transformations eventually rely on the ability of triflate to leverage the trans influence and coordination flexibility of the PSiP ligand to assist proton shifts.

## Results and Discussion

### Catalytic Observations

**Figure 1:**
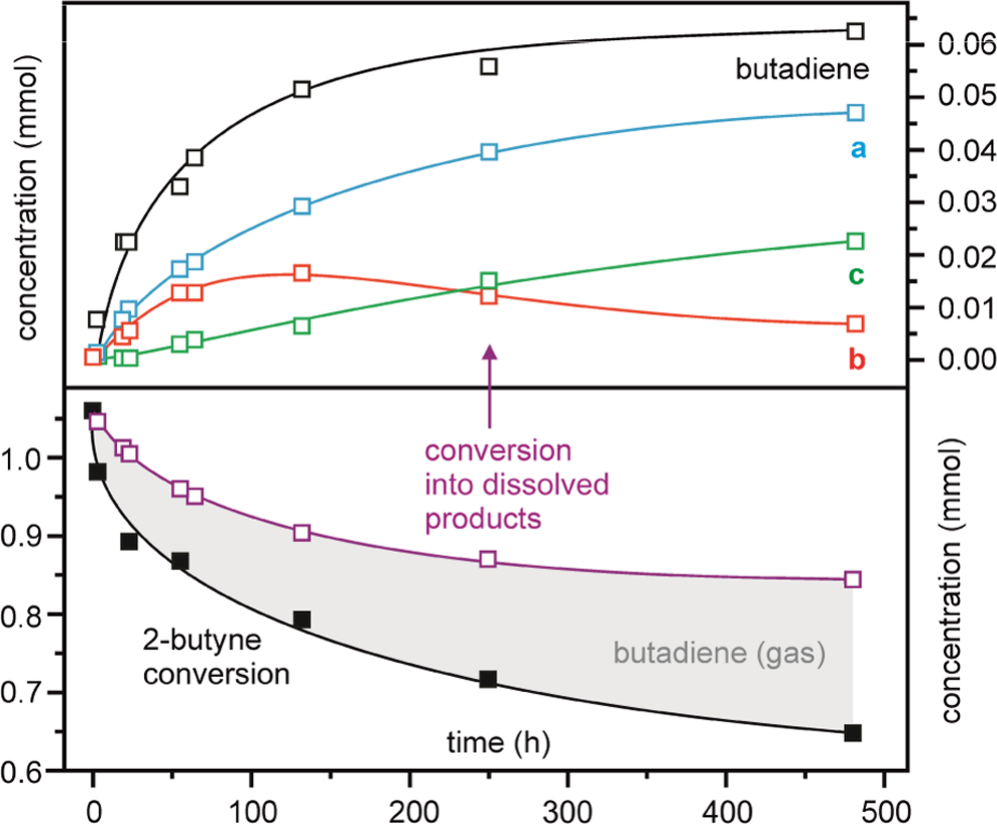
Product evolution with time for the catalytic transformation of 2-butyne. Conditions: C_6_D_6_ (0.4 mL), 363 K, **1** (4 mg, 0.0052 mmol).

In C_6_D_6_ solution, complex **1** was found to slowly transform 2-butyne into the mixture of products shown in Scheme 1. Reproducible initial TOFs around 5 h^–1^ were obtained under 200-fold alkyne excess in sealed NMR tubes at 363 K (for further experimental details, see the Supporting Information). As shown in the reaction profiles of Figure 1, the main catalytic course was alkyne isomerization into butadiene, which was mostly released to the gas phase. The outcome composition also changed throughout the reaction because of the thermal electrocyclization of **b**, (3*Z*)-3,4-dimethyl-1,3,5-hexatriene, into **c**, 2,3-dimethyl-1,3-cyclohexadiene, which is a likely process in view of the literature results.^38−40^ Attending to the expected coordination capabilities of the reaction products, the progressive slowdown and eventual deactivation could be attributed to catalyst inhibition by products, as will be further substantiated below.

The catalytic transformation of Scheme 1 became nonefficient away from the experimental conditions specified in Figure 1. Replacement of the C_6_D_6_ solvent with the slightly more polar C_6_D_5_Cl gave much slower reactions that deactivate after just a couple of turnovers at 363 K. The reactions were also found nonproductive in acetone-*d*_6_ at 333 K, or in C_6_D_5_Cl at 363 K using the related cationic catalyst precursor [IrH(PSiP)(NCMe)_2_]BF_4_. Precursor [IrHCl(PSiP)] was found active in C_6_D_6_, although it produced reactions much slower than its triflate analogue, and formed 1,3-butadiene but not dimerization products. A graphic comparison of reaction profiles under these mentioned conditions is shown in the Supporting Information (Figure S7). At least in part, catalyst deactivation in solvents such as C_6_D_5_Cl could result from the presence of adventitious water, which was observed to irreversibly modify the catalyst Ir(PSiP) scaffold with concomitant, diagnostic, formation of 2-butene, as will be further illustrated below.

### Intermediates Search

**Figure 2:**
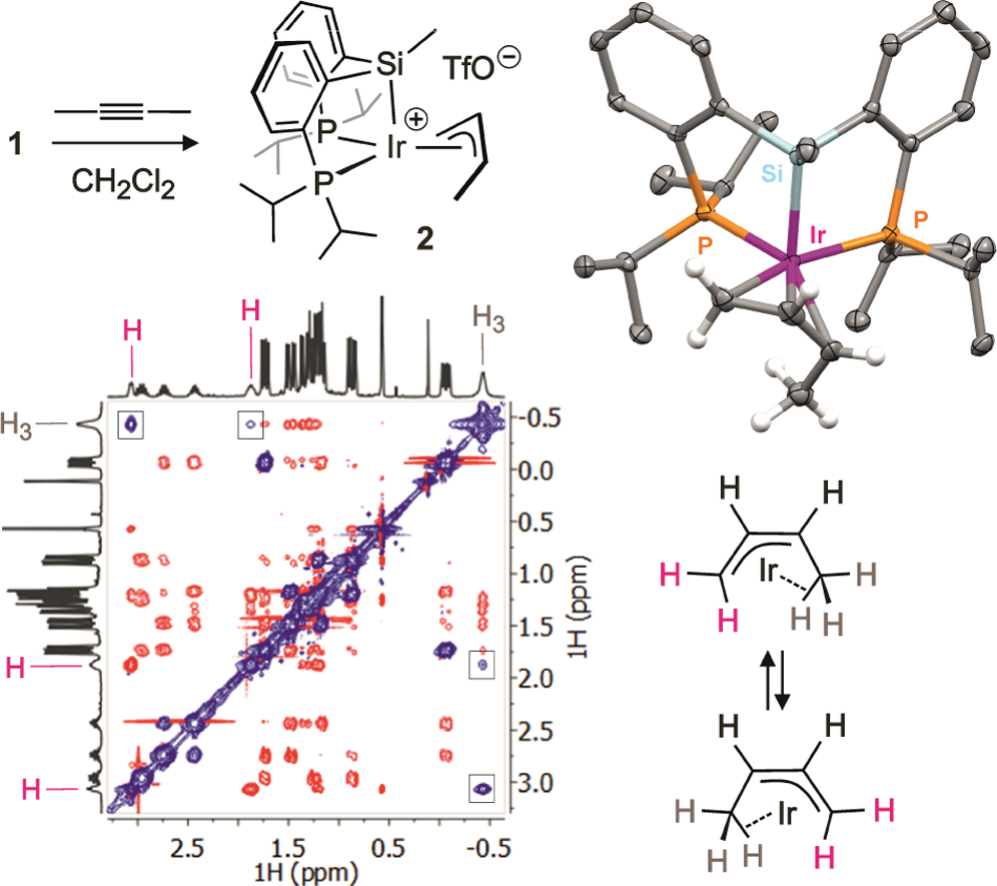
Preparation of **2**, X-ray structure of its cation (H atoms of the PSiP ligand are omitted for clarity), and part of the ^1^H NOESY NMR spectrum (CD_2_Cl_2_, 298 K) evidencing the H-atom exchange within the methylallyl ligand.

The reaction between **1** and 2-butyne in moderate excess (2–3 equiv), in dichloromethane as solvent, produced the isolable cationic complex [Ir(η^3^-CH_2_CHCHMe){κ*P,P,Si*-SiMe(C_6_H_4_-2-*Pi*Pr_2_)_2_}](CF_3_SO_3_) (**2**, Figure 2). The structure determined by X-ray diffraction in crystals obtained from this solution displays a *fac*-coordinated PSiP together with a η^3^-methylallyl ligand. The NMR spectra of **2** in CD_2_Cl_2_ are consistent with this solid-state structure, showing two doublets with a *cis* mutual coupling constant of 5.1 Hz in the ^31^P{^1^H} spectrum, and ^1^H multiplets at δ 1.84, 3.03, 5.13, and 5.82 attributable to the four hydrogens of the allyl skeleton. The X-ray structure of the complex also evidences an agostic interaction with the methyl substituent of the allyl ligand at the, otherwise vacant, coordination position trans to Si. The refined Ir–H distance in this interaction is 2.21(5) Å, while the *J*_CH_ coupling constant determined in the ^13^C INEPT NMR signal of this methyl (δ 8.96) was 121.6 Hz. This is just slightly below that of the methyl group at silicon (128.1 Hz) though still compatible with an agostic CH averaged in the NMR timescale with two nonagostic ones.^41^

Interestingly, the ^1^H NMR NOESY spectrum of **2** at room temperature (Figure 2) evidences intraligand H exchange among the agostic methyl and both methylene hydrogens at the other side of the allyl. Given that such an exchange symmetrizes the cation, it also causes exchange cross-peaks between inequivalent fragments of the PSiP ligand on each side of the molecule. Pseudo-first-order kinetic constants for this process could be obtained in any set of exchanging ^1^H NMR signals, via spin saturation transfer (spin labeling or EXSY) or linewidth analysis, depending on the temperature. Those determined in C_6_D_5_Cl in the temperature range 300–363 K led to activation parameters Δ*H*^‡^ = 19(±1) kcal mol^–1^ and Δ*S*^‡^ = 2(±2) cal K^–1^ mol^–1^, in agreement with an intramolecular process (for details, see the Supporting Information). Attending to the features of complex **2**, the H-atom exchange is likely to involve a hypothetical symmetric hydride-butadiene intermediate. Accordingly, prolonged heating of **2** in C_6_D_5_Cl led to the progressive release of butadiene with the regeneration of **1**, though the reaction was very slow and accompanied by partial decomposition. The treatment of **2** with an excess of acetonitrile also produced butadiene, in this case with clean formation of the known six-coordinate cationic hydride [IrH(PSiP)(NCMe)_2_](CF_3_SO_3_).^26^ These reactions outline a likely end for the 2-butyne to 1,3-butadiene isomerization pathway in which product release is the last and the likely rate-limiting step.

**Figure 3:**
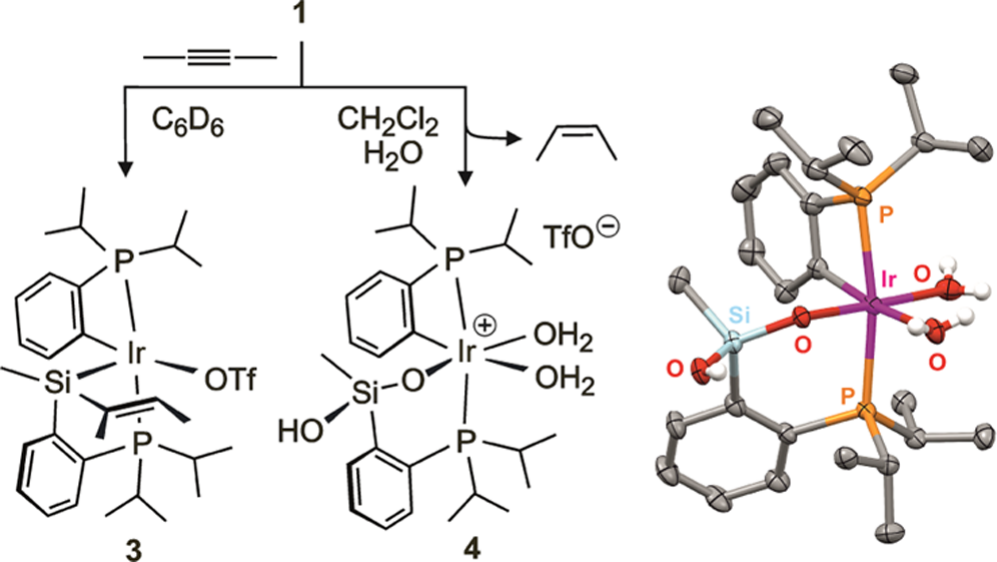
Formation of complexes **3** and **4** and X-ray structure of the cation of **4** (H atoms except those coming from water are omitted for clarity).

In contrast to that observed in chlorinated solvents, the reaction between **1** and 2-butyne in C_6_D_6_ did not produce cationic complex **2**, at least not initially, but mainly the isomeric complex [Ir{κ*O*-O_3_S(CF_3_)}{κ*P,Si*-SiMe(*Z*-CMe=CHMe)(C_6_H_4_-2-*Pi*Pr_2_)}(κ*C,P*-C_6_H_4_-2-*Pi*Pr_2_)] (**3**) (Figure 3). Yet, the lifetime of **3** in this solution was found to be relatively short, quantitatively yielding crystals of **2** after a few hours at room temperature. The ^31^P{^1^H} NMR spectrum of **3** displays two doublets at disparate chemical shifts, δ 59.44 and −19.19, with a *J*_PP_ coupling constant that evidences mutually trans phosphorus: 298.9 Hz. The ^1^H NMR spectrum indicates the presence of the *Z*-alkenyl moiety expected from 2-butyne insertion into the Ir–H bond, though none of this moiety’s resonances shows coupling with any of the phosphorus atoms. Instead, a clear cross-peak between the methyl at the alkenyl α carbon and the silicon atom is seen in the ^1^H/^29^Si HMBC correlation (Figure S18). In addition, a set of four ^1^H NMR aromatic signals at unusual chemical shifts, from δ 6.05 to 6.62, displays relatively large *J*_HP_ coupling constants but not ^1^H/^29^Si HMBC correlations at all, suggesting the cleavage of a Si–C bond in the pincer ligand backbone accompanied by metalation of the resulting aryl. Similar reversible disassembling processes of this PSiP ligand have been previously recognized in Ni and Pd complexes.^42−46^

The structural proposal for **3** in Figure 3 includes triflate coordination, which “a priori” would prevent excessive unsaturation at the metal and unlikely charge separation in benzene solution. Besides, it is compatible with the ^19^F NMR signal: a broad singlet at δ −77.58, close to that observed for the starting complex **1**. In fact, the need to accommodate the triflate at the coordination sphere of the complex to remain soluble in C_6_D_6_ might make the difference in this solvent, triggering alkenyl moiety migration to silicon, just as observed for other Si–C forming reactions in Ru(PSiP) pincers provoked by an increase of the metal coordination number.^47,48^ Si–C bond cleavages and formations leading to **3** must be reversible, as the solutions of **3** eventually yield crystals of **2**. Accordingly, the use as catalyst precursor of aliquots of benzene solutions containing **3** led to results comparable to those using isolated complexes **1** or **2**.

A somehow related disassembling of the PSiP ligand was observed when the synthesis of **2** in CD_2_Cl_2_ was attempted in the presence of small amounts of added water. This reagent provoked a rapid release of 2-butene with concomitant formation of a new major complex, [Ir{κ*O,P*-OSiMe(OH)(C_6_H_4_-2-*Pi*Pr_2_)}(κ*C,P*-C_6_H_4_-2-*Pi*Pr_2_)(OH_2_)_2_](CF_3_SO_3_) (**4**, Figure 3), which shows NMR features that resemble those of **3**: very different trans phosphorus in the ^31^P{^1^H} NMR spectrum as well as a ^1^H NMR pattern suggesting an orthometalated PC chelate ligand. The complex formed crystals suitable for an X-ray diffraction study that led to the structure shown in Figure 3. It indeed displays a pincer ligand split into PC and PO chelate fragments, as a result of two Si–O bond formations^49−52^ and a Si–C cleavage. It further exemplifies that PSiP silicon functionalization, irreversible in this case, may trigger Si–C bond cleavage, as proposed in the formation of **3**. In addition, it indicates the moisture sensitiveness of the catalytic system, which is in contrast to the compatibility with water demonstrated by **1** in the absence of 2-butyne.^26^ Complex **2** was found to be water-compatible too, hence an unobserved intermediate capable of releasing 2-butene is the likely moisture-sensitive weak link of the 2-butyne isomerization cycle. The solutions containing **4** were confirmed to be inactive catalyst precursors.

**Scheme 2:**
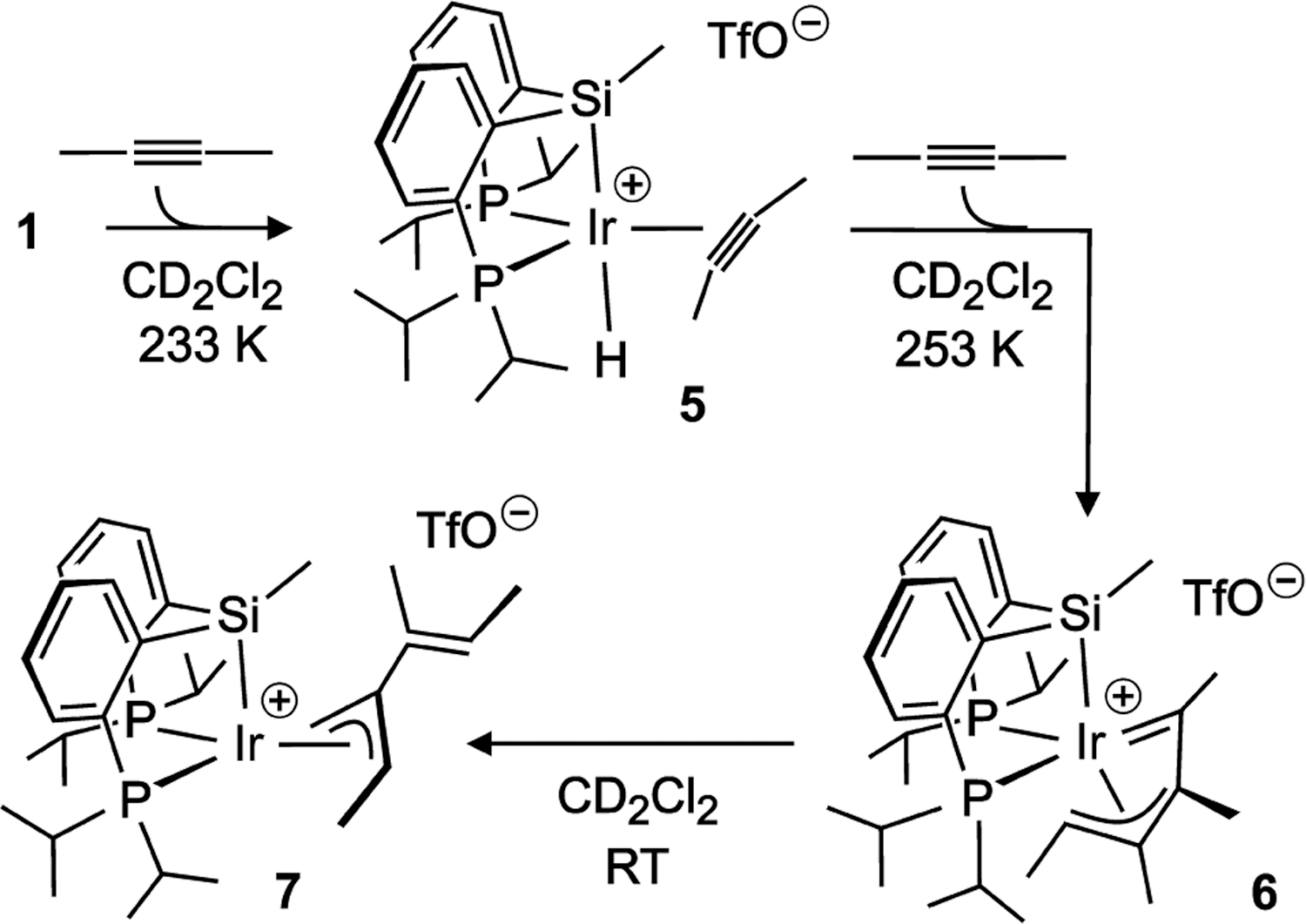
Complexes Observed in CD_2_Cl_2_ at a Low Temperature

The room temperature chemistry described above was extended through low-temperature studies that led to the identification of further possible reaction intermediates. At 233 K in CD_2_Cl_2_, the addition of 3–4 equiv of 2-butyne to solutions of **1** gave rise to a new set of ^1^H NMR signals that suggest alkyne coordination to form [IrH{κ*P,P,Si*-SiMe(C_6_H_4_-2-*Pi*Pr_2_)_2_}{η^2^-CMe≡CMe}](CF_3_SO_3_) (**5**, Scheme 2). In particular, there is a new hydride triplet shifted about 15 ppm toward low field with respect to that of **1**, and a singlet attributable to coordinated 2-butyne, again downfield the resonance of free 2-butyne. Attending to its ^31^P{^1^H} NMR singlet resonance, the adduct may retain the *mer* coordination of the PSiP ligand after alkyne binding, although the chemical equivalence of the P atoms would also be compatible with a *fac* PSiP arrangement if triflate does not coordinate (the option chosen in Scheme 2). Deciding on the latter is not obvious from the unique broad signal in the ^19^F NMR spectrum, though its chemical shift (δ −78.94) is more consistent with a free anion. Noteworthy, the latter would be in contrast to that previously observed in adducts of **1** with smaller incoming ligands such as dihydrogen.^26^

Adduct **5** slowly disappeared upon increasing the temperature to 253 K, selectively forming complex [Ir{κ*C*, η^3^-CMeCMeCMeCHMe}{κ*P,P,Si*-SiMe(C_6_H_4_-2-*Pi*Pr_2_)_2_}](CF_3_SO_3_) (**6**, Scheme 2). Along with a ^31^P{^1^H} pattern confirming *fac* PSiP coordination, the NMR spectra of **6** reveal the incorporation of two alkyne molecules: evident from the presence of four nonequivalent methyl group resonances in the ^1^H and ^13^C{^1^H} spectra. The bonding between the two former alkyne fragments is confirmed by the ^1^H COSY NMR spectrum, which shows cross-peaks correlating up to three of these methyl groups. The most characteristic NMR signals of the newly assembled ligand are a unique ^1^H CH at δ 4.95, a quartet featuring a *J*_HH_ coupling constant of 6.4 Hz, and a low field doublet in the ^13^C{^1^H} spectrum, δ 239.85, *J*_CP_ = 65.6 Hz, indicative of a carbene moiety. Overall, the NMR information led to the structural proposal of Scheme 2, which displays a butadienyl ligand in the κ*C*, η^3^ carbene-allyl coordination mode. This mode, which spans three *fac* coordination positions, has been previously recognized in related d^4^ and d^6^ complexes,^53−56^ and is expected to prevail over the simpler η^2^-alkenyl alternative^57^ or the κ*C*, η^2^ (alkenyl-alkene) bidentate mode, which is the preferred option in d^8^ square-planar environments.^58^ In any case, such a possible binding versatility is likely related to the dynamic behavior of the complex evidenced by the exchange peaks in the ^1^H NOESY NMR spectrum. As for **2**, the dynamic process of **6** renders equivalent halves of the PSiP ligand although, unlike **2**, the other ligand, the butadienyl in this case, does not evidence intraligand H exchange nor increases its symmetry (Figure S39). Hence, rather than a C–H bond activation, the dynamic process of **6** likely involves just a change in the butadienyl coordination mode allowing a transient planar conformation^59,60^ within the Ir–Si–Me plane.

When the solutions of **6** in CD_2_Cl_2_ were warmed to room temperature, the complex was observed to transform into a new species [Ir{η^3^-CH_2_C(*Z*-CMe=CHMe)CHMe}{κ*P,P,Si*-SiMe(C_6_H_4_-2-*Pi*Pr_2_)_2_}](CF_3_SO_3_) (**7**, Scheme 2). Even though the reaction was rather selective (above 80%), minor unidentified compounds were also formed. The NMR spectra of **7** are reminiscent of those of allyl complex **2**, in particular because of the four ^1^H multiplets corresponding to Hs of the ligand skeleton, at δ 1.57, 3.48, 5.84, and 6.21 in this case. Just like in **2**, the first two correspond to methylene hydrogens whereas the last two are CHs. Yet, unlike **2**, the ^1^H COSY NMR spectrum of **7** indicates that there is no coupling between the CH_2_ and any of the CHs (Figure S44), which implies that none of the CHs is adjacent to the methylene. Under this premise, the alkenyl-methyl-substituted allyl ligand proposed in Scheme 2 is the only possible option.

As for **2**, the allyl’s methyl substituent of **7** is significantly shielded (^1^H and ^13^C NMR signals at δ −0.38 and 9.03, respectively) and also seems to exchange with the methylene protons. In this case, the exchange cannot symmetrize **7** but generates a different isomer instead. Attending to its calculated energy (see below), this second isomer cannot explain the accompanying minor signals of the NMR spectra, which are more likely due to hindered conformational changes, as several exchange cross-peaks in the ^1^H NOESY seem to relate **7** with the minor products (Figure S47). Notably, once again in parallel with **2**, the H-β-elimination likely involved in the allyl intraligand H exchange of **7** would produce coordinated dendralene **a**.

### Intermediates Modeling and Mechanism

Optimized structures (PBE1PBE/def2-svp) and energies (wb97xd/def2-tzvpp) were calculated for all experimentally observed complexes described in the previous section. In most cases, the DFT calculations in gas phase found several possible conformational minima for each structure, those of lowest energy consistently matching the structures deduced by NMR, ^1^H NOESY spatial relationships included (see the Supporting Information). The structure calculated for the cation of **6** (**[6**_**calc**_**]**^**+**^) also supports the proposed κ*C*, η^3^ carbene-allyl coordination as the more stabilizing, though an unsaturated isomer showing the η^2^-alkenyl alternative, **[6′**_**calc**_**]**^**+**^, 10.4 kcal mol^–1^ above, might account for the easy symmetrization observed in solution.

**Figure 4:**
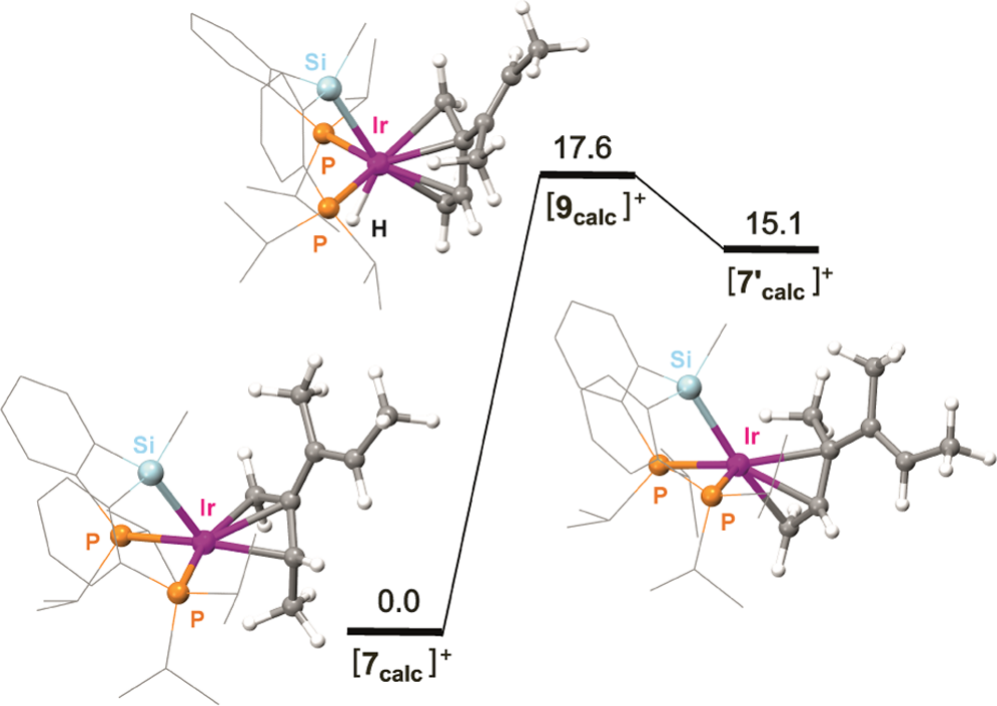
Hydrido-dendralene **[9**_**calc**_**]**^**+**^ and allyl intermediates calculated for the intraligand H-atom exchange in cationic complex **7**.

Calculations were extended to the intraligand H-atom exchange observed for the methylallyl complex **2** and its proposed mechanism, which may comprise a hydrido-butadiene intermediate (**[8**_**calc**_**]**^**+**^) 12.2 kcal mol^–1^ above **[2**_**calc**_**]**^**+**^ (Figure S48). The free energy of the transition state calculated for the exchange process (**[TS**_**2–8**_**]**^**+**^), 18.0 kcal mol^–1^, matches that experimentally determined in C_6_D_6_Cl, 18.4(±1.6) kcal mol^–1^. Even though the optimized structure of **[8**_**calc**_**]**^**+**^ is not symmetric and hence cannot fully explain the experimentally observed process, we assume that its energy still offers margin for conformational changes leading to symmetrization. Intermediate **[9**_**calc**_**]**^**+**^, the hydrido-dendralene analogue of **[8**_**calc**_**]**^**+**^, was found 17.6 kcal mol^–1^ above intermediate **[7**_**calc**_**]**^**+**^, while the other side of the proposed intra-allyl H-atom exchange, **[7′**_**calc**_**]**^**+**^, lies 15.1 kcal mol^–1^ above and displays the structure shown in Figure 4.

**Figure 5:**
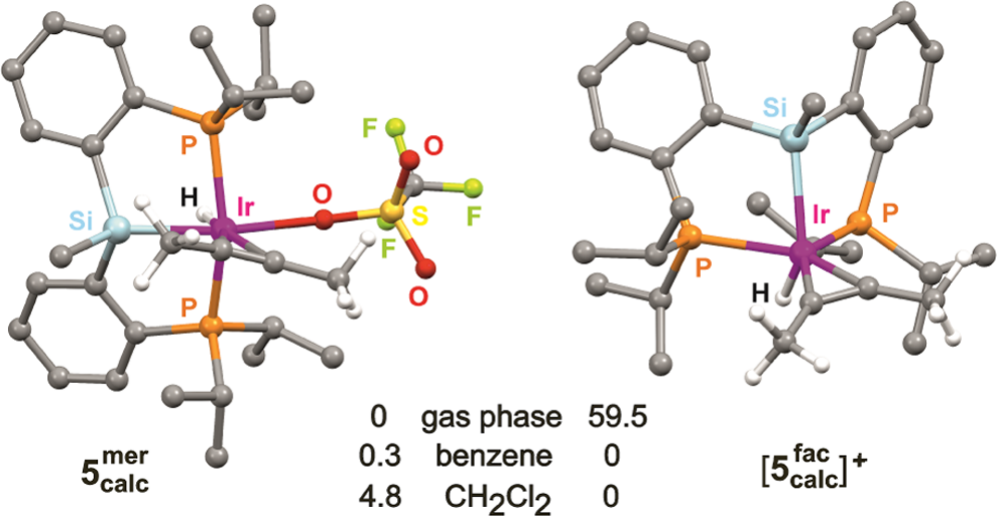
Calculated structures for **5** and their relative Gibbs free energies in different media (kcal mol^–1^).

The possible coordination of triflate anion came out as a major uncertainty for modeling. Figure 5 depicts two structures calculated for complex **5**, very different from each other but both compatible with the symmetry observed by NMR in CD_2_Cl_2_. As expected, that with a coordinated triflate and a *mer* PSiP ligand (**5**_**calc**_^**mer**^) is clearly favored in the gas phase, but the alternative ion pair with a *fac*-coordinated PSiP (**[5**_**calc**_^**fac**^**](OTf)**) becomes slightly more favorable when considering solvents, benzene or CH_2_Cl_2_, using the PCM solvation model. Unfortunately, the difference in Gibbs free energy between the two options is too small to indicate a clear preference: an ambiguity that persists beyond the alkyne coordination step. Still, the following discussion will show that triflate coordination is indeed a relevant mechanistic issue, although it is not until the product release step that it becomes determinant for catalytic turnover.

**Figure 6:**
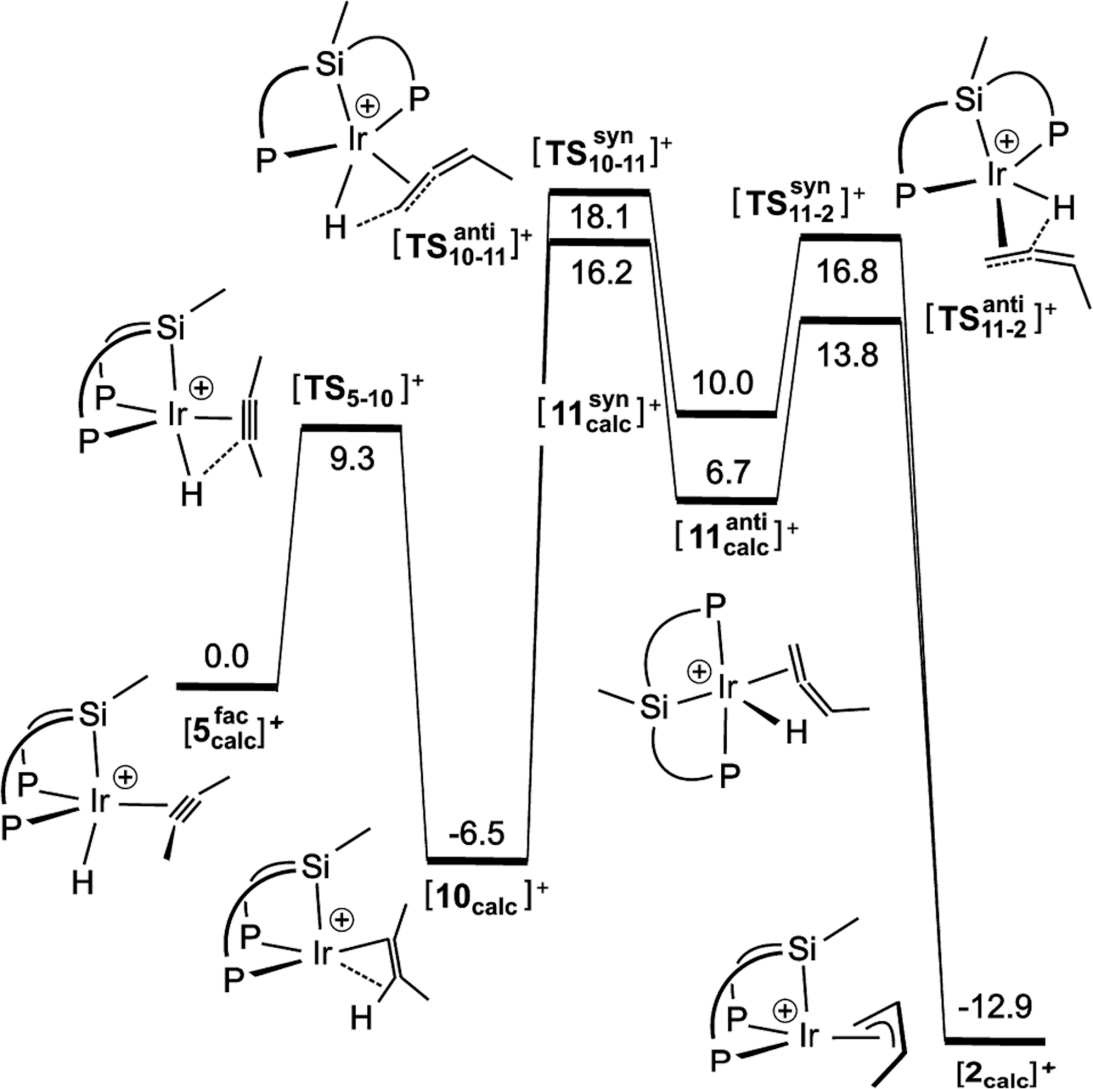
Calculated pathway for the transformation of **5** into **2** through the classical Green’s mechanism. Gibbs free energies in benzene (kcal mol^–1^). The drawings for the *syn* TSs have been omitted for the sake of clarity. C···H = 1.6–1.8 Å. For full geometric details, see the Supporting Information.

The transformation of alkynes into allyl ligands at the coordination sphere of late transition-metal complexes is key for certain catalytic functionalizations affording branched structures.^61,62^ A possible mechanism via hydrido-allene intermediates was suggested by Werner and Wolf in Rh(Cp) complexes^63^ following early proposals by Green et al. in Mo derivatives.^64,65^Figure 6 summarizes the free energy profile corresponding to this mechanism in benzene, starting from the first observable intermediate **5** (**[5**_**calc**_^**fac**^**]**^**+**^) and finishing in the final reaction product at room temperature **2** (**[2**_**calc**_**]**^**+**^). The highest barrier in this profile is 24.6 kcal mol^–1^, hence compatible with a room temperature reaction. Again, calculations found several minima for intermediates **[10**_**calc**_**]**^**+**^ and **[11**_**calc**_**]**^**+**^, although for the sake of clarity, only those of lowest energy are represented in Figure 6 (and described in the Supporting Information). Given that catalyst offers two different faces (*syn* or *anti*) for hydride β-elimination and subsequent insertion, trajectories of different energy are possible at each face, although differences are small.

After a new alkyne coordination and alkenyl migratory insertion in intermediate **[10**_**calc**_**]**^**+**^, this mechanism could also account for the transformations and intermediates formed upon coupling of two equivalents of alkyne: those en route from **6** to **7**. Yet, such extrapolation would not directly afford **7** from **6**, but only via isomer **7′** and the additional transformation shown in Figure 4.

**Figure 7:**
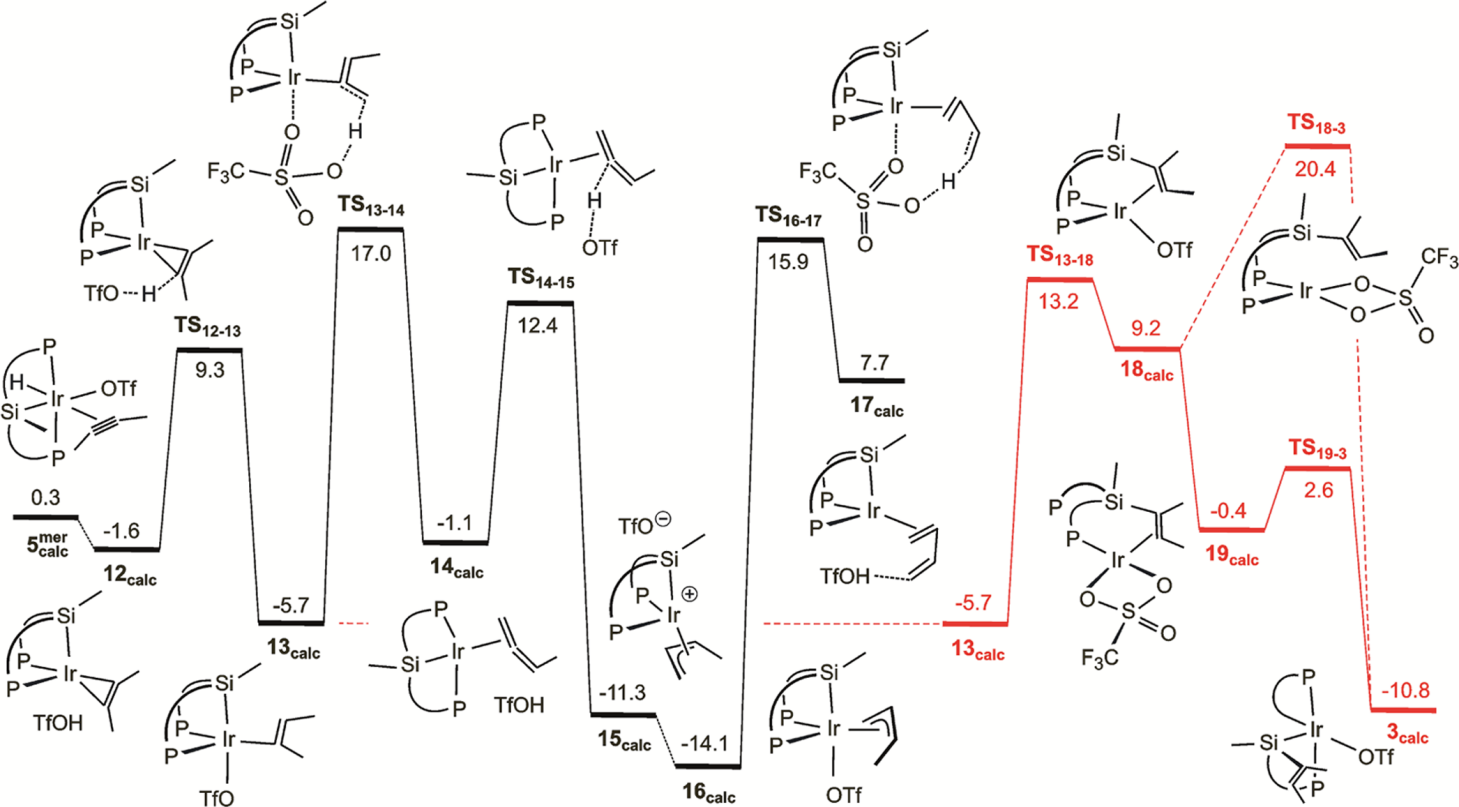
Calculated mechanism for the transformation of 2-butyne into 1,3-butadiene, and PSiP ligand disassembling (in red). The Gibbs free energy scale is the same as that in Figure 6. Ir···O = 2.4–2.7 Å, C···H = 1.3–2.0 Å, O···H = 1.1–1.4 Å. For full geometric details, see the Supporting Information.

Triflate coordination to iridium hampers the above mechanism moving H atoms as hydrides but enables an alternative that shuttles them as protons (Figure 7). This possibility has already been demonstrated for anionic ligands such as carboxylates in catalytic transformations initiated by ligand-assisted C–H bond cleavages through the so-called CMD mechanism.^66^ After cleavage, protons can be transferred to external bases or alternative ligands^67^ or return to an alternative position of the original ligand. In the latter tautomerization processes, the overall mechanism is often termed LAPS (ligand-assisted proton shuttle).^68^ Such abilities to move protons are less expected for the less basic triflate,^69^ although in coordination positions trans to silicon, it might harness their characteristic high trans influence^70^ to dock without significant loss of electron density. In fact, all calculated intermediates of Figure 7 featuring triflates trans to silicon display Ir–O distances above 2.3 Å, well beyond the mean value found in the CCDC for coordinated triflates,^71^ 2.22 Å, and also longer than those calculated for precursor **1** or complex **3**: about 2.15 Å in both cases.

Calculations in benzene solution (Figure 7) indicate that isomer **5**_**calc**_^**mer**^ can favorably rearrange (−1.3 kcal mol^–1^) into triflic acid and intermediate **12**_**calc**_. According to its pseudo-tetrahedral geometry and the structural parameters of the alkyne moiety, this calculated product of formal triflic acid reductive elimination should be described as containing a four-electron 2-butyne ligand.^72^ Remarkably, the acid can readily re-protonate this complex, directly in one of the alkyne carbons, to form alkenyl **13**_**calc**_. Despite the different mechanisms, the energy profiles leading to alkenyl complexes **[10**_**calc**_**]**^**+**^ and **13**_**calc**_ from isomers **5**_**calc**_ display similar barriers. To the best of our knowledge, this outer-sphere protonation alternative to alkyne insertion in metal–hydride bonds has not been previously discussed, in spite of the fact that it could readily explain selectivities (anti-additions, anti-Markovnikov, etc.) often observed in the broad context of catalytic alkyne functionalization.^73^

Given that an incidental movement of either triflic acid or triflate away from iridium is likely under this mechanism, multiple attack trajectories to the alkyne ligand or any of its transformations may be conceivable. In consequence, those in Figure 7 should be better regarded as just a mechanism verification rather than an optimized proposal. Still, a comparison of profiles in Figures 6 and 7 evidences only minor benefits in moving protons over moving hydrides, which suggests that both mechanisms could compete in the formation of methylallyl complexes. However, the presence of triflate in the vicinity of the complex enables a butadiene-releasing pathway that is not feasible in its absence. It again implies an intramolecular formation of triflic acid, in this case from the triflate and methylallyl ligands of intermediate **16**_**calc**_, which is the most stable in benzene as solvent. The deprotonation transition state (**TS**_**16–17**_) features a barrier of 30.0 kcal mol^–1^, consistent with the sluggishness of the overall catalytic reaction. Noteworthy, this calculated barrier raises to an unreachable 38.8 kcal mol^–1^ in solvents such as dichloromethane (Figure S50), mainly because of the additional energy necessary to coordinate triflate to the solvated cation **[2**_**calc**_**]**^**+**^, which is the preferred option in this solvent. Triflic acid formation leads to unsaturated η^2^-butadiene intermediate **17**_**calc**_, in which we propose an alkene-by-alkyne replacement reforming **12**_**calc**_ as the cycle closing step. This termination sequence would also be plausible attending to the observed catalyst inhibition by products.

Just like in the case of moving hydrides, we propose that the mechanism of moving protons and its benefits could be extrapolated to intermediates containing dimeric ligands en route to products **a** and **b**. In this respect, hypothetical analogues of **16**_**calc**_ with an additional *Z*-C(Me)=CHMe alkenyl substituent (that present in **7**) may offer up to three methyl groups susceptible to deprotonation, likely within the reach of a loosely coordinated triflate. From such an intermediate, a final deprotonation step similar to **TS**_**16–17**_ would form dendralene **a**, whereas an alternative deprotonation of the terminal methyl group of the alkenyl substituent would yield conjugated triene **b**. Extrapolation of this mechanism would also be conceivable for catalyst precursor [IrHCl(PSiP)] since chloride has been recognized as capable of shuttling protons in related ligand tautomerization processes.^74^ Yet, although better than triflate from the p*K*_a_ point of view, chloride should be less versatile to reach acidic sites and less prone to give way to a second alkyne equivalent.

Finally, our calculations explored the possible participation in catalysis of compounds such as **3** that feature disassembled PSiP ligands. The formation of **3** from calculated alkenyl **13**_**calc**_, in red in Figure 7, may involve the expected Si–C reductive elimination/oxidative addition sequence, though the latter requires a previous triflate-assisted dissociation of a phosphine arm. Only this way, calculations afford barriers compatible with the experimental observation of **3** prior to crystallization of **2**. Yet, our exploration of hydride or proton movements in intermediates such as **18**_calc_, **19**_calc_, or **3**_calc_ has not identified possible kinetic advantages over the mechanisms in Figures 6 and 7, in particular none affecting the rate-limiting release of products. In this respect, **3** may resemble other PSiP ligand functionalization products observed in Pd catalysis, recognized as mere off-cycle resting states without significance for catalytic turnover.^46^

## Conclusions

The coordination environment of complex [IrH(OTf)(PSiP)] (**1**) gathers a particularly rich arsenal of mechanistic resources applicable to catalytic transformation of organic molecules. Besides classical options in common with other transition-metal hydrides, it includes reversible Si–C bond cleavages and formations previously recognized in PSiP pincers, as well as the ability of triflate to move protons that emerges from this study. All of those resources seem mutually compatible and could operate simultaneously, though only the last one makes possible the eventual release of strongly coordinating diene and polyene products that unlocks catalysis. We suggest that the nature of solvent, which disfavors charge separation, and the low electronic demand to bind at the position trans to silicon combine to keep triflate close to the metal and basic enough to accomplish deprotonations of the organic moiety. We believe this positive combination of ligand properties to be transferable to other metal complexes and conditions, to optimize reactions such as those studied here or accomplish other challenging catalytic syntheses.

## Supporting Information Available

Catalytic and synthetic experimental procedures, characterization data, computational details, calculated energies, and additional schemes and figures (PDF)
Coordinates of optimized complexes, intermediates, and transition states (xyz)

## Supplementary Material

om2c00375_si_001.pdf

om2c00375_si_002.xyz
