# Supplementary material for: Mechanistic Versatility at Ir(PSiP) Pincer Catalysts: Triflate Proton Shuttling from 2-Butyne to Diene and [3]Dendralene Motifs
Source: Organometallics. 2022 Sep 13;41(18):2622–30. doi: 10.1021/acs.organomet.2c00375 (PMC9518705; doi:10.1021/acs.organomet.2c00375)
Supplement: Supplementary file 1 — om2c00375_si_001.pdf [file om2c00375_si_001.pdf]

Supporting Information for:

Mechanistic versatility at Ir(PSiP) pincer catalysts: Triflate proton shuttling from 2-butyne to diene and [3]dendralene motifs

José L. Andrés, Elizabeth Suárez, Marta Martín and Eduardo Sola*

Instituto de Síntesis Química y Catálisis Homogénea (ISQCH), CSIC–Universidad de Zaragoza, E50009, Spain. E-mail: sola@unizar.es

Contents:

General information 2

Catalytic reactions 2

Synthesis of the complexes and observation of intermediates 8

Preparation of **2** 7

Intraligand H exchange kinetics for complex **2** 11

3 13

4 17

5 22

6 23

7 27

Structural analysis of **2**, [IrH{κP,P,Si-SiMe(C_6H_4-2-P*i*Pr_2)$_2$}($NCCH_3$)$_2$](BF_4) and **4** 32

Computational details 33

Energies of the calculated compounds and transition states 33

References 36

General information.

All manipulations were carried out with exclusion of air by using standard Schlenk techniques or in an argon-filled drybox (MBraun). Solvents were obtained from a solvent purification system (MBraun). Deuterated solvents were dried with appropriate drying agents and degassed with argon prior to use. C, H and N analyses were carried out in a Perkin-Elmer 2400 CHNS/O analyzer. NMR spectra were recorded on Bruker Avance 400 or 300 MHz spectrometers. ^{1}H (400.13 or 300.13 MHz) and ^{13}C (100.6 or 75.5 MHz) NMR chemical shifts were measured relative to partially deuterated solvent peaks but are reported in ppm relative to TMS. ^{19}F (376.5 MHz), ^{31}P (162.0 or 121.5 MHz) and ^{29}Si (79.5 or 59.6 MHz) chemical shifts were measured relative to $CFCl_3$, H_3PO_4 (85%) and TMS, respectively. Coupling constants are given in hertz. In general, NMR spectral assignments were achieved through $^{1}H\{^{1}H\}$, ^{1}H COSY, ^{1}H NOESY, $^{1}H\{^{31}P\}$, ^{13}C APT, and $^{1}H/^{13}C$ HSQC experiments. Unless otherwise indicated, the NMR data are given at room temperature.

Precursor complex $[IrH\{\kappa O\text{-}O_3S(CF_3)\}\{\kappa P,P,Si\text{-}SiMe(C_6H_4\text{-}2\text{-}P\textit{i}Pr_2)_2\}]$ (**1**),[1] and catalyst precursor $[IrHCl\{\kappa P,P,Si\text{-}SiMe(C_6H_4\text{-}2\text{-}P\textit{i}Pr_2)_2\}]$[2] were prepared as previously described. Cationic catalyst precursor $[IrH\{\kappa P,P,Si\text{-}SiMe(C_6H_4\text{-}2\text{-}P\textit{i}Pr_2)_2\}(NCCH_3)_2](BF_4)$ was prepared through a modification of the synthesis reported for its triflate analogue $[IrH\{\kappa P,P,Si\text{-}SiMe(C_6H_4\text{-}2\text{-}P\textit{i}Pr_2)_2\}(NCCH_3)_2](CF_3SO_3)$,[1] as described below. All other reagents were commercial and were used as received.

Preparation of $[IrH\{\kappa P,P,Si\text{-}SiMe(C_6H_4\text{-}2\text{-}P\textit{i}Pr_2)_2\}(NCCH_3)_2](BF_4)$. A solution of $[IrHCl\{\kappa \textit{P,P,Si}\text{-}SiMe(C_6H_4\text{-}2\text{-}P\textit{i}Pr_2)_2\}]$[1] (131.6 mg, 0.20 mmol) in acetone (5 mL) was treated with silver tetrafluoroborate (38.9 mg, 0.20 mmol) and stirred for 16 h in the dark. The resulting suspension was stored at 253 K for one day, then filtered through Celite to remove the insoluble silver chloride and taken to dryness. The addition of acetonitrile/diethyl ether (0.05/5 mL) produced a pale-yellow solid, which was separated by decantation, washed with diethyl ether, and dried in vacuo. Yield: 95 mg (60%). Anal. Calcd for $C_{29}H_{46}BF_4N_2P_2SiIr$: C, 43.99; H, 5.86; N, 3.54. Found: C, 43.72; H, 5.55; N, 3.20. ^{1}H and $^{31}P\{^{1}H\}$ NMR spectra coincide with those previously reported for the triflate analogue. ^{19}F NMR ($CDCl_3$): δ −153.3 (s). The crystals used in the X-ray diffraction experiment were obtained from a mixture of diethyl ether and acetonitrile solution stored at 253 K.

Catalytic reactions.

In a typical procedure, 2-butyne (86 μL, 1.06 mmol) and mesitylene (internal reference, 30 μL) were added through a micro syringe to a solution of complex **1** (4.0 mg, 5.18 μmol) in the desired deuterated solvent (0.4 mL), contained in a sealable (PTFE-valved) NMR tube. The tube was immersed in a heating bath at the desired reaction temperature and was followed by ^{1}H and $^{13}C\{^{1}H\}$ NMR. Spectra were recorded at intervals at 298 K, that is after removing the sample from the heating bath and cooling it. Given the slowness of the reactions, the impact of this sampling method was assumed to be negligible and no corrections were applied.

All reaction products were previously described and were identified by their ^{1}H and $^{13}C\{^{1}H\}$ NMR spectra with the aid of the corresponding correlation spectra.

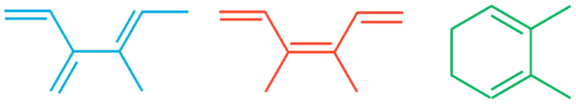

a **b** **c**

(**a**) 1,2-dimethyl-[3]dendralene or 4-methyl-3-methylene-1,4-hexadiene (CAS Registry Numbers 26198-74-3 and 674283-55-7):[3] ^{1}H NMR (C_6D_6, 298 K): δ 1.52 (dq, $^{3}J_{HH}$ = 6.7, $^{5}J_{HH}$ = 1.0, 3H, CH_3), 1.70 (dq, $^{4}J_{HH}$ = 1.3, $^{5}J_{HH}$ = 1.0, 3H, CH_3), 4.99 (m, $^{2}J_{HH}$ = 1.9, 1H, $=CH_2$), 5.02 (dddd, J_{HH} = 10.6, 2.0,

0.7, 0.5, 1H, $=CH_2$), 5.05 (dm, $^2J_{HH}$ = 1.9, 1H, $=CH_2$), 5.31 (dddd, J_{HH} = 17.3, 2.0, 0.4, 0.4, 1H, $=CH_2$), 5.61 (qq, $^3J_{HH}$ = 6.7, $^4J_{HH}$ = 1.3, 1H, =CH), 6.42 (ddd, J_{HH} = 17.3, 10.6, 0.9, 1H, =CH). $^{31}C\{^1H\}$ NMR (C_6D_6, 298 K): δ 13.85, 15.13 (CH_3), 112.50, 115.95 ($=CH_2$), 123.76 (=CH), 135.26 (C), 138.23 (=CH), 151.16 (C).

(**b**) 3,4-dimethyl-(3*Z*)-1,3,5-hexatriene (CAS Registry Numbers 76401-93-9 and 132911-36-5):[4] ^1H NMR (C_6D_6, 298 K): δ 1.72 (s, 6H, CH_3), 5.02 (dd, $^3J_{HH}$ = 10.9, $^2J_{HH}$ = 1.4, 2H, $=CH_2$), 5.16 (dd, $^3J_{HH}$ = 17.0, $^2J_{HH}$ = 1.4, 2H, $=CH_2$), 7.05 (dd, $^3J_{HH}$ = 17.0, $^3J_{HH}$ = 10.9, 2H, =CH). $^{31}C\{^1H\}$ NMR (C_6D_6, 298 K): δ 14.93 (CH_3), 113.32 ($=CH_2$), 130.85 (C), 135.02 (=CH).

(**c**) **2,3-dimetyl-1,3-ciclohexadiene** (CAS Registry Number 4430-91-5):[5] ^1H NMR (C_6D_6, 298 K): δ 1.67 (m, 6H, CH_3), 1.98 (m, 4H, CH_2), 5.53 (m, 2H, =CH). $^{31}C\{^1H\}$ NMR (C_6D_6, 298 K): δ 19.88 (CH_3), 23.39 (CH_2), 122.23 (=CH), 133.94 (C).

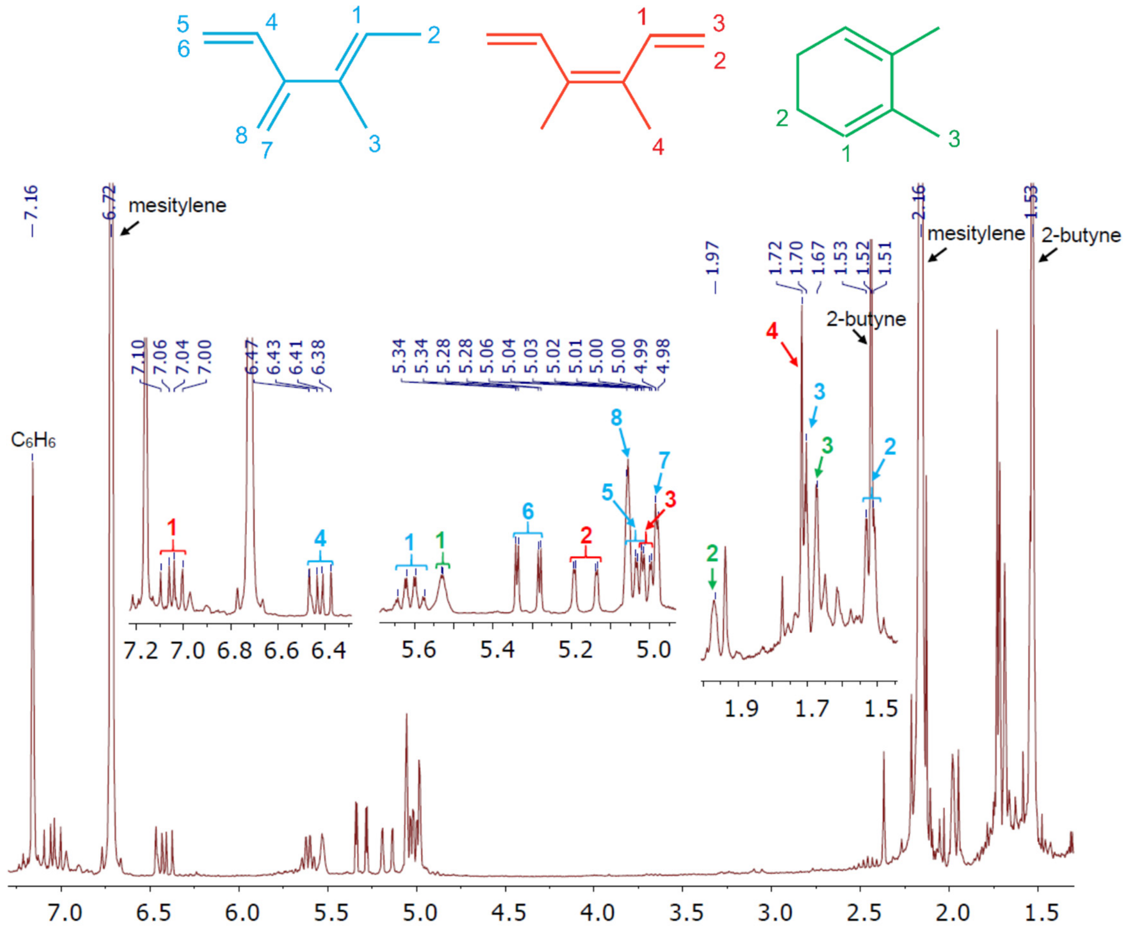

Figure S1. ^1H NMR spectrum (298 K, C_6D_6) of the catalytic reaction outcome. A stream of argon was previously bubbled through to remove the butadiene.

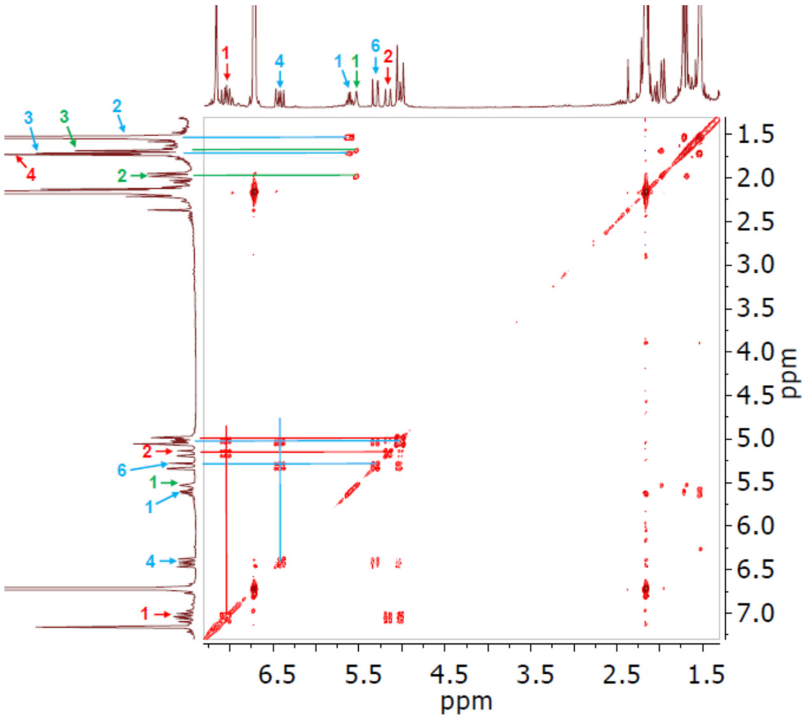

Figure S2. ^{1}H COSY NMR spectrum (298 K, C_6D_6) of the catalytic reaction outcome. A stream of argon was previously bubbled through to remove the butadiene.

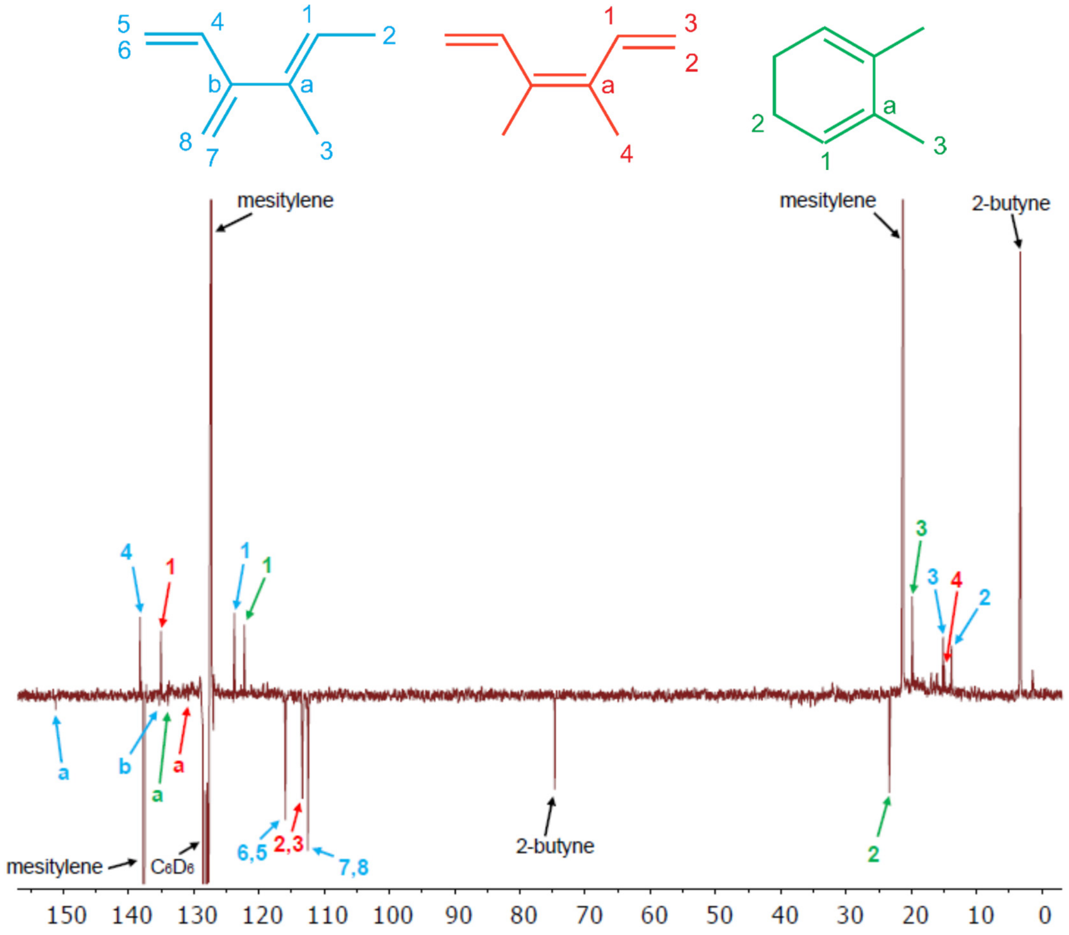

Figure S3. $^{31}C\{^{1}H\}$ APT NMR spectrum (298 K, C_6D_6) of the catalytic reaction outcome. A stream of argon was previously bubbled through to remove the butadiene.

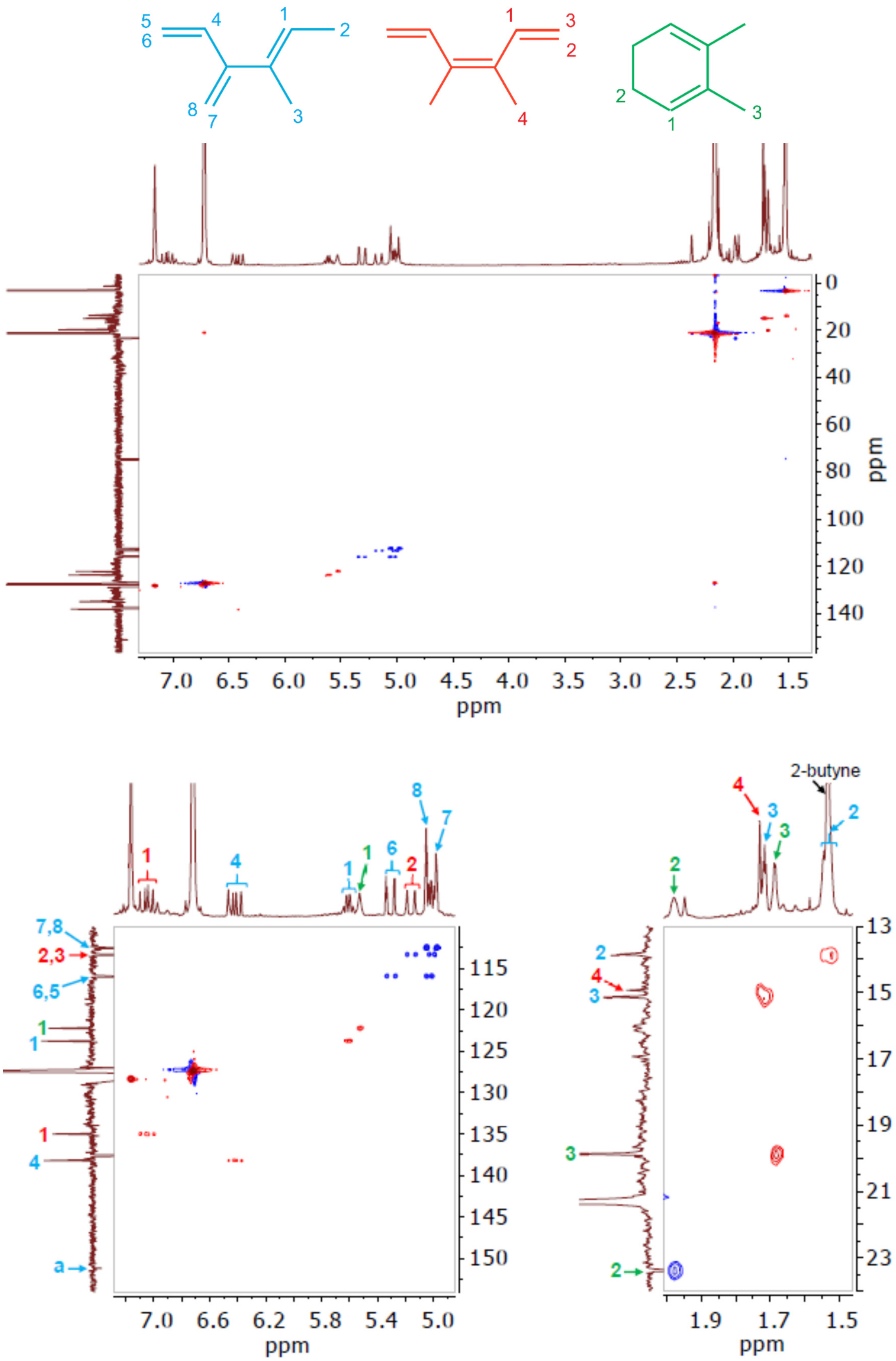

Figure S4. $^1H/^{13}C$ HSQC-edited NMR spectrum (298 K, C_6D_6) of the catalytic reaction outcome. A stream of argon was previously bubbled through to remove the butadiene.

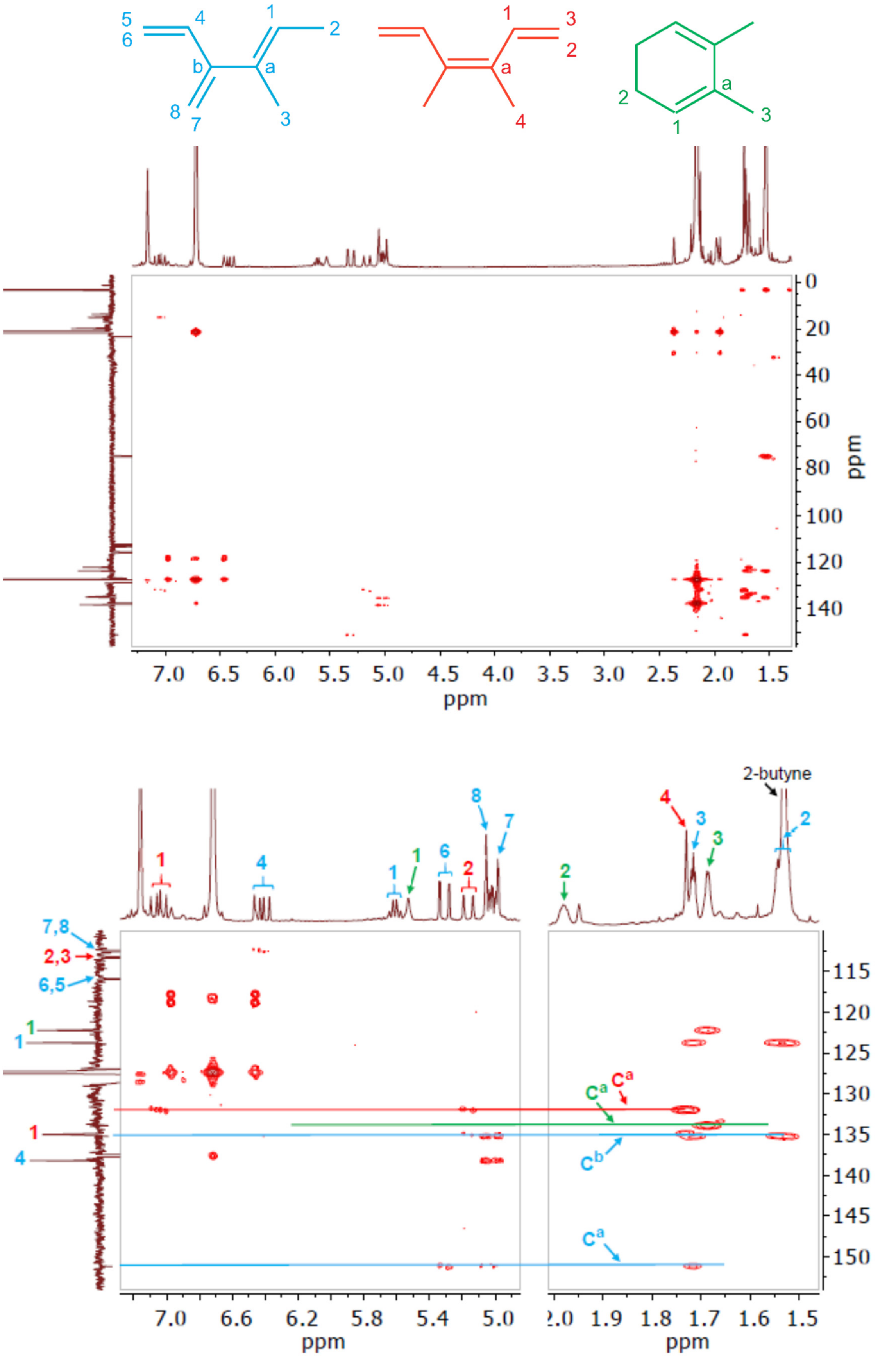

Figure S5. $^{1}H/^{13}C$ HMBC NMR spectrum (298 K, C_6D_6) of the catalytic reaction outcome. A stream of argon was previously bubbled through to remove the butadiene.

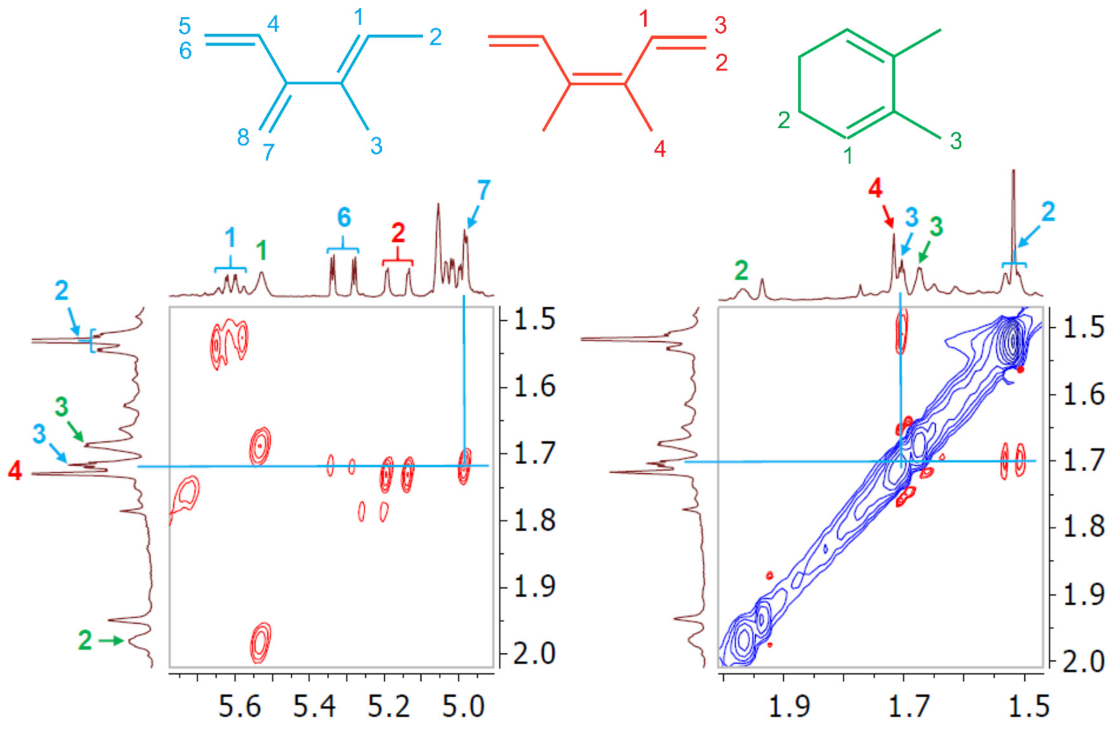

Figure S6. Selected sections of the ^{1}H NOESY NMR spectrum (298 K, C_6D_6) of the catalytic reaction outcome. A stream of argon was previously bubbled through to remove the butadiene.

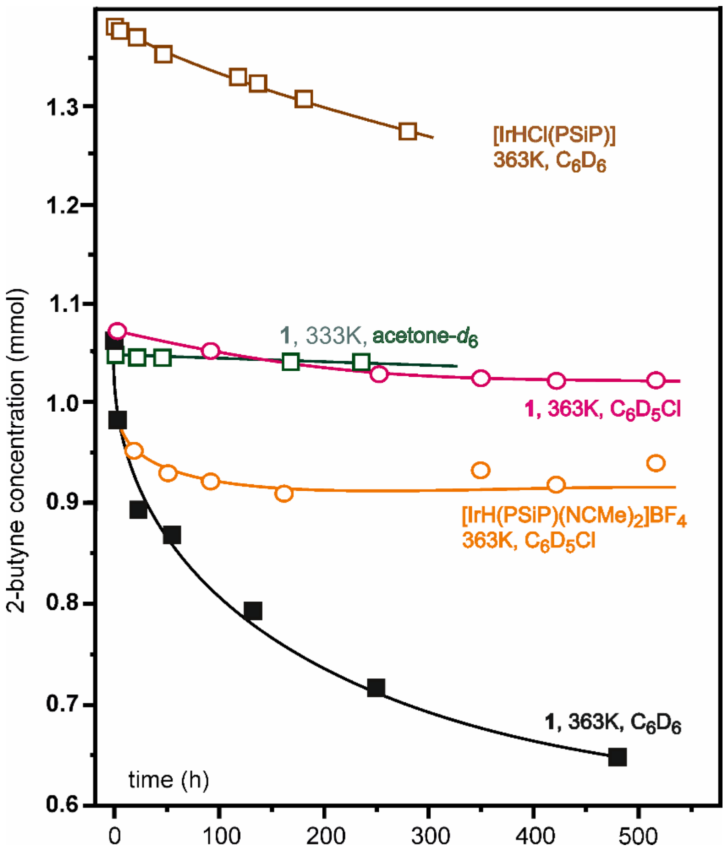

Figure S7. Reaction profiles of catalytic reactions using different catalyst precursors, solvents, and temperatures.

Synthesis of the complexes and observation of intermediates.

Preparation of [Ir(η^3-$CH_2CHCHMe$){κP,P,Si-SiMe(C_6H_4-2-P*i*Pr_2)$_2$}](CF_3SO_3) (2): 2-butyne (35 µL, 0.45 mmol) was added to a solution of **1** (154 mg, 0.20 mmol) in CH_2Cl_2 (10 mL), and stirred for 24 h at room temperature. The solvent was evaported to ca. 0.5 mL and hexane (3 mL) was added to produce a yellow solid, which was dried in vacuo. Yield 150 mg (91 %). Anal. Calcd. for $C_{30}H_{46}O_3F_3IrP_2SiS$: C, 43.62; H, 5.61; S, 3.88. Found: C, 44.00; H, 5.81; S; 3.90. ^{1}H NMR (CD_2Cl_2): δ −0.47 (br, 3H, $CH_2CHCHCH_3$), −0.10 (dd, $^3J_{HP}$ = 16.3, $^3J_{HH}$ = 7.0, 3H, $PCHCH_3$), 0.53 (s, 3H, $SiCH_3$), 0.83 (dd, $^3J_{HP}$ = 15.7, $^3J_{HH}$ = 6.9, 3H, $PCHCH_3$), 1.10-1.23 (m, 9H, $PCHCH_3$), 1.30 (dd, $^3J_{HP}$ = 18.4, $^3J_{HH}$ = 7.1, 3H, $PCHCH_3$), 1.44 (dd, $^3J_{HP}$ = 18.1, $^3J_{HH}$ = 7.6, 3H, $PCHCH_3$), 1.70 (dd, $^3J_{HP}$ = 12.6, $^3J_{HH}$ = 7.0, 3H, $PCHCH_3$), 1.84 (m, 1H, $CH_2CHCHCH_3$), 2.40, 2.70, 2.93 (all m, 1H each, $PCHCH_3$), 3.03 (m, 1H, $CH_2CHCHCH_3$), 3.39 (m, 1H, $PCHCH_3$), 5.13 (m, 1H, $CH_2CHCHCH_3$), 5.82 (m, 1H, $CH_2CHCHCH_3$), 7.36 (m, 2H, CH), 7.47 (m, 1H, CH), 7.54 (m, 3H, CH), 7.76 and 7.81 (both m, 1H each, CH). ^{19}F NMR (CD_2Cl_2): δ −78.80 (s). ^{31}P{^{1}H} NMR (CD_2Cl_2): δ 46.94, 47.25 (both d, $^2J_{PP}$ = 5.1). ^{13}C{^{1}H} NMR (CD_2Cl_2): δ 0.57 (d, $^3J_{CP}$ = 1.7, $SiCH_3$), 8.96 (br, $CH_2CHCHCH_3$), 18.79, 19.16 (both s, $PCHCH_3$), 19.28 (d, $^2J_{CP}$ = 1.8, $PCHCH_3$), 19.43 (d, $^2J_{CP}$ = 5.3, $PCHCH_3$), 19.65 (s, $PCHCH_3$), 19.75 (d, $^2J_{CP}$ = 3.8, $PCHCH_3$), 20.43 (d, $^2J_{CP}$ = 4.9, $PCHCH_3$), 24.22 (d, $^2J_{CP}$ = 5.8, $PCHCH_3$), 26.18 (dd, $^1J_{CP}$ = 32.4, $^3J_{CP}$ = 1.7, $PCHCH_3$), 28.19 (d, $^1J_{CP}$ = 23.7, $PCHCH_3$), 30.91 (dd, $^1J_{CP}$ = 26.4, $^3J_{CP}$ = 1.1, $PCHCH_3$), 31.92 (dd, $^1J_{CP}$ = 29.8, $^3J_{CP}$ = 3.1, $PCHCH_3$), 41.61 (d, $^2J_{CP}$ = 24.9, $CH_2CHCHCH_3$), 75.96 (d, $^2J_{CP}$ = 12.2, $CH_2CHCHCH_3$), 100.36 (br, $CH_2CHCHCH_3$), 121.51 (q, $^1J_{CF}$ = 320.6, CF_3SO_3), 131.40 (d, J_{CP} = 4.0, CH), 129.80 (d, J_{CP} = 7.2, CH), 129.90 (d, J_{CP} = 4.8, CH), 130.09 (d, J_{CP} = 6.6, CH), 131.23 (d, J_{CP} = 2.2, CH), 131.81 (d, J_{CP} = 2.3, CH), 132.47 (d, J_{CP} = 19.2, CH), 132.80 (d, J_{CP} = 19.4, CH), 137.94 (d, $^1J_{CP}$ = 57.9, C), 141.67 (d, $^1J_{CP}$ = 53.2, C), 151.65 (d, $^2J_{CP}$ = 38.5, C), 152.27 (dd, $^2J_{CP}$ = 39.1, $^3J_{CP}$ = 1.1, C). ^{29}Si{^{1}H} NMR (CD_2Cl_2): δ 26.70 (brdd, J_{SiP} = 5.2, $J_{SiP'}$ < linewidth). The complex was observed to be air-sensitive in solution and solid state. The crystals used in the X-ray diffraction experiment were obtained from a hexane solution stored at 253 K.

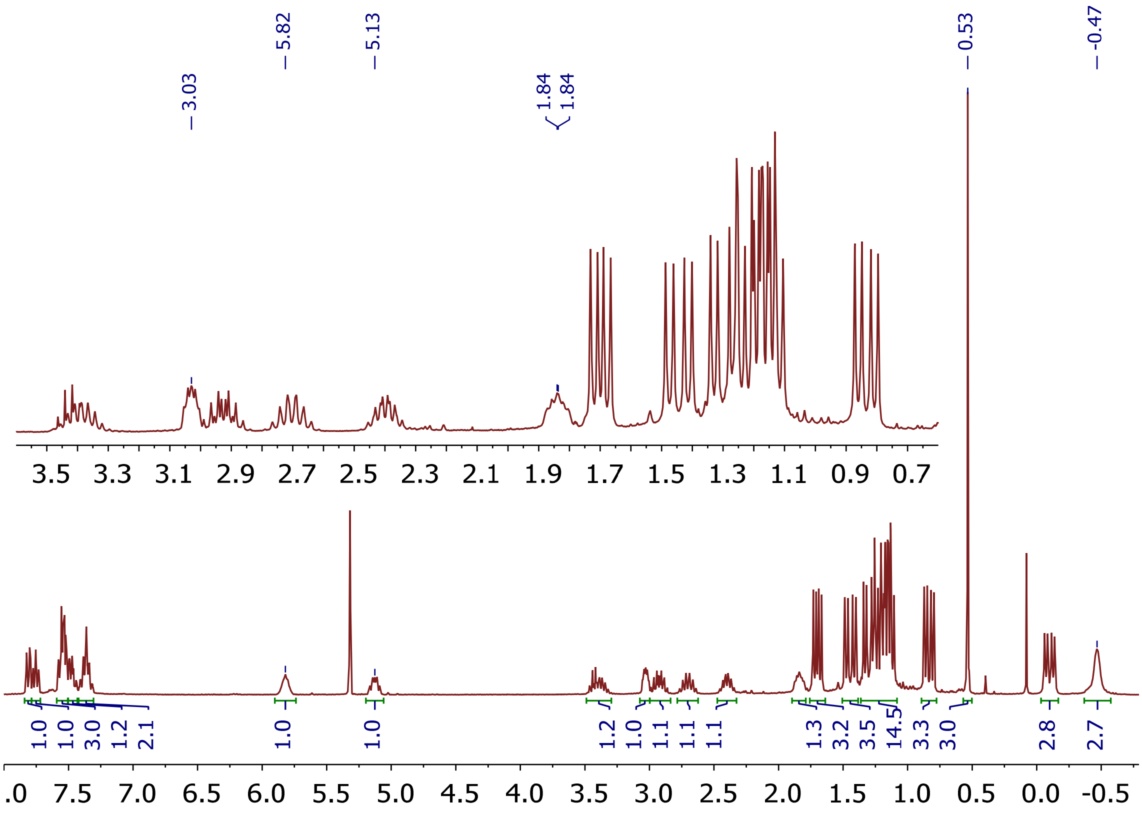

Figure S8. ^{1}H NMR spectrum of **2** in CD_2Cl_2 at 298 K.

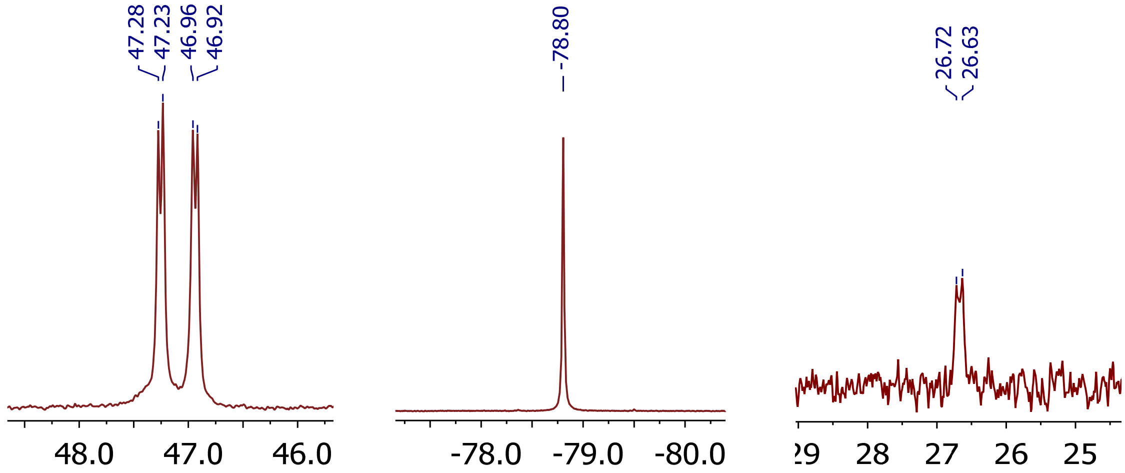

Figure S9. (left) $^{31}P\{^{1}H\}$, (center) ^{19}F, and (right) $^{29}Si\{^{1}H\}$ NMR spectra of complex **2** in CD_2Cl_2 at 298 K.

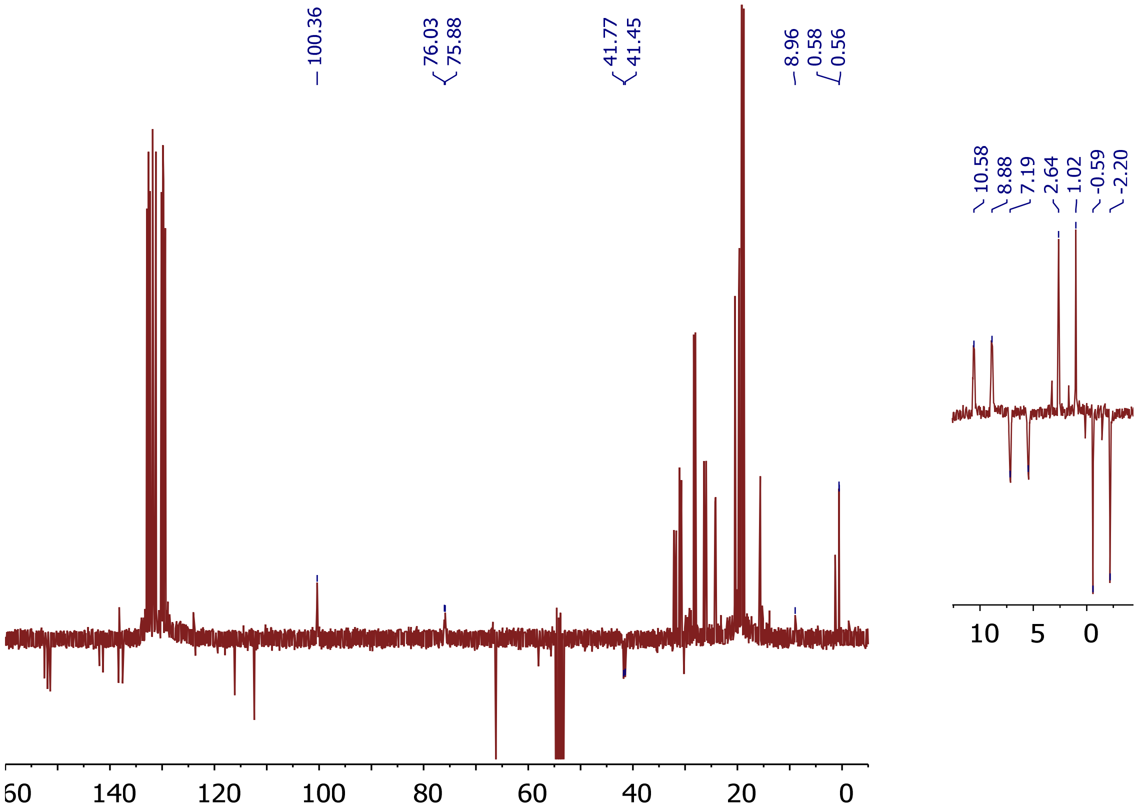

Figure S10. ^{13}C NMR spectra of complex **2** in CD_2Cl_2: (left) $^{13}C\{^{1}H\}$ APT at 298 K, and (right) detail of the INEPT at 233 K.

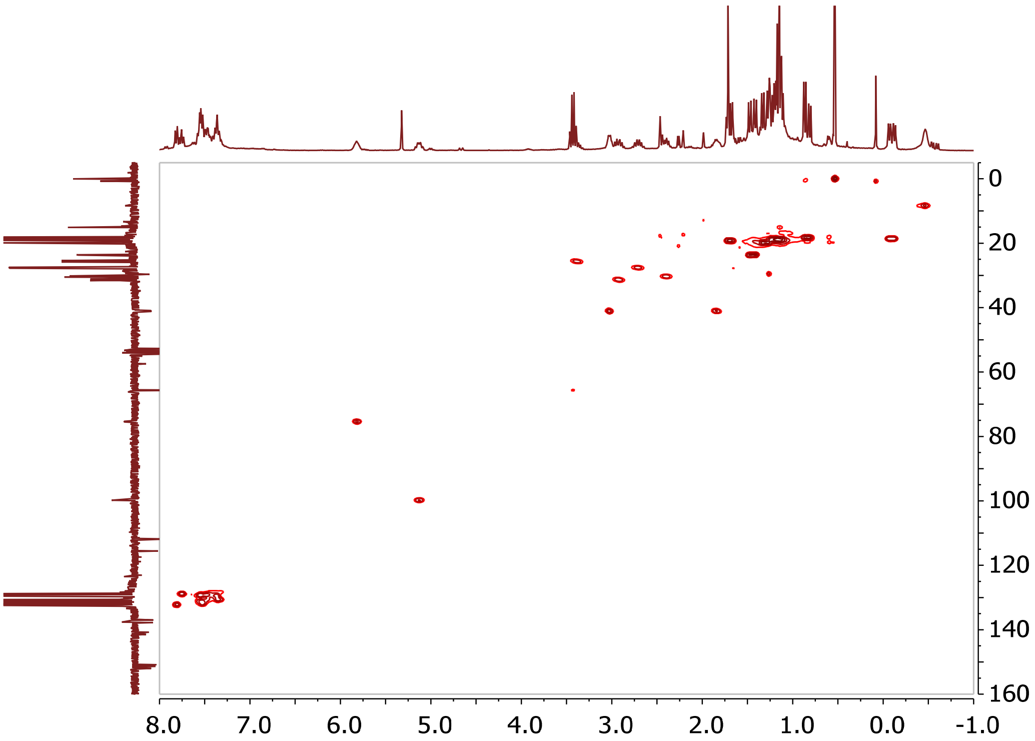

Figure S11. $^{1}H/^{13}C$ HSQC NMR spectrum of complex **2** in CD_2Cl_2 at 298 K.

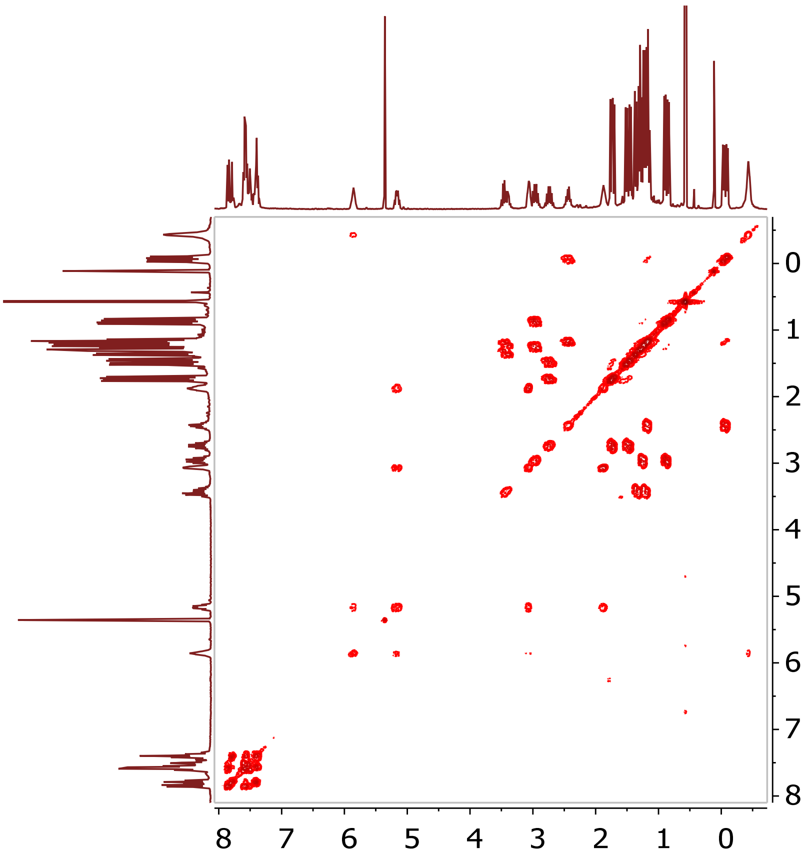

Figure S12. ^{1}H COSY NMR spectrum of complex **2** in CD_2Cl_2 at 298 K.

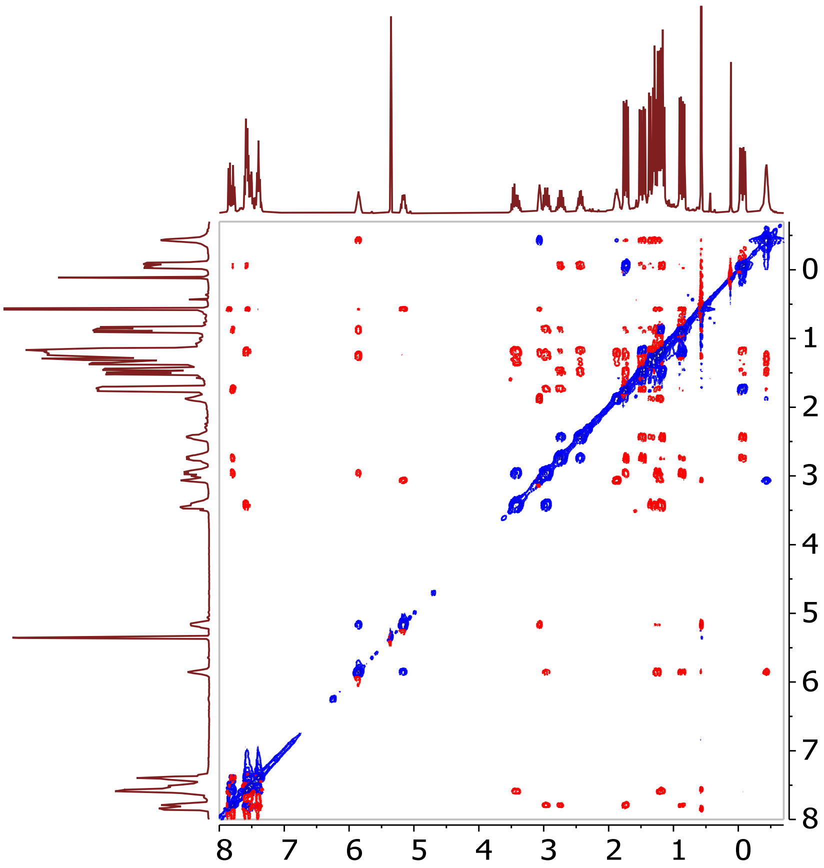

Figure S13. ^{1}H NOESY NMR spectrum of complex **2** in CD_2Cl_2 at 298 K.

Intraligand H exchange kinetics for complex 2. Pseudo first-order rate constants (k_{obs}) for the exchange were determined by spin saturation transfer following either EXSY[6] or spin labeling[7] methods. In the latter, the pre-saturation pulse was applied at the ^{1}H NMR signal corresponding to the methylene hydrogen at δ 3.03, and its effect was measured in the integral of the broad singlet corresponding to agostic methyl group. In this case $k = 3\ k_{obs}$ to account for the population of the ^{1}H NMR signal used for determinations. In the EXSY experiments, any set of exchanging signals was convenient for calculation of k using the EXSYCalc free software (https://mestrelab.com/software/freeware/). The temperature range of the experimental determinations was extended using rate constants derived from linewidths. The figure below shows a methyl signal of the PSiP ligand and the corresponding simulations. Again, any signal involved in the exchange was suitable for such analysis. Rate constants k were used to estimate the activation parameters via the Eyring representation of the figure below. Errors in $\Delta H^{\ddagger}$ and $\Delta S^{\ddagger}$ were estimated through conventional error propagation formulas,[8,9] assuming 1K error in the temperature and a 10% error in the experimental rate constant.

Table S1. Rate constants for intraligand H exchange in complex 2

Method	Temp (K)	k_{obs} (s^{-1})	k (s^{-1})
EXSY	300	0.25	0.25
Spin labeling	300	0.07	0.21
EXSY	308	0.78	0.78
Spin labeling	313	0.39	1.17
Spin labeling	318	0.54	1.62
Spin labeling	323	0.75	2.25
Line width	333	7.2	7.2
Line width	343	18.3	18.3
Line width	353	39	39
Line width	363	68	68

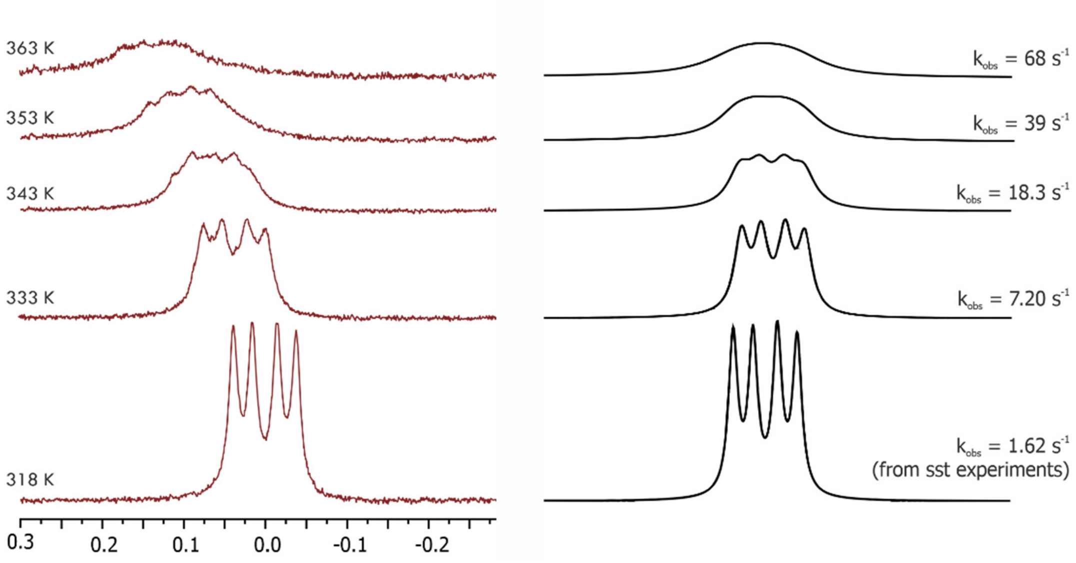

Intercept	24.8019 ± 0.81405
Slope	-9554.38 ± 263.31
R-square	0.99396

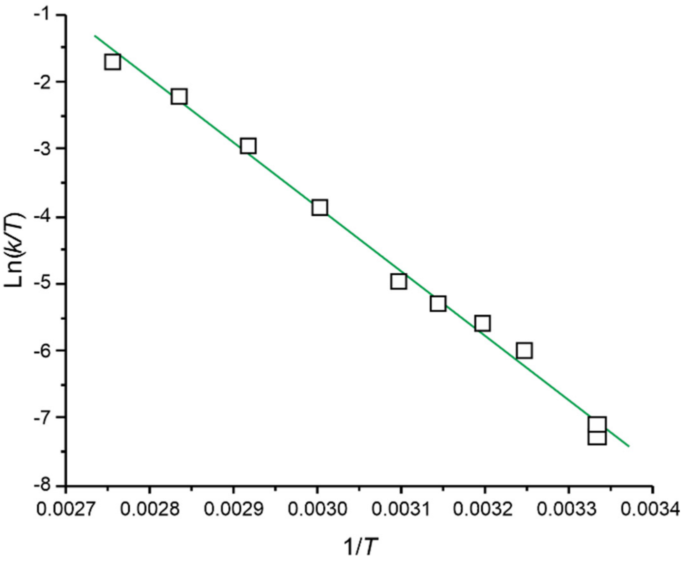

Figure S14. Above: Experimental (left) and calculated (right) ^{1}H NMR signals of a PSiP methyl of **2**. Below: Eyring representation of the rate constants.

[Ir{κO-$O_3S(CF_3)$}{κP,Si-SiMe(*Z*-CMe=CHMe)(C_6H_4-2-P*i*Pr_2)}(κC,P-C_6H_4-2-P*i*Pr_2)] (3): 2-butyne (24 µL, 0.31 mmol) was added to a solution of **1** (5.4 mg, 7.0 µmol) in C_6D_6 (0.4 mL). The NMR spectra revealed formation of **3** as the major reaction product: ^{1}H NMR (C_6D_6): δ −0.17 (s, 3H, $SiCH_3$), 0.78-1.11 (m, 12H, PCHCH_3), 1.22 (dd, $^3J_{HP}$ = 12.6, $^3J_{HH}$ = 7.2, 3H, PCHCH_3), 1.27 (dd, $^3J_{HP}$ = 14.3, $^3J_{HH}$ = 7.0, 3H, PCHCH_3), 1.44 (dd, $^3J_{HP}$ = 18.6, $^3J_{HH}$ = 6.9, 3H, PCHCH_3), 1.50 (dd, $^3J_{HP}$ = 15.9, $^3J_{HH}$ = 6.4, 3H, PCHCH_3), 1.81 (dq, $^3J_{HH}$ = 6.5, $^5J_{HH}$ = 1.0, 3H, C(CH_3)=CHCH_3), 1.91 (dq, $^4J_{HH}$ = 1.7, $^5J_{HH}$ = 1.0, 3H, C(CH_3)=$CHCH_3$), 2.55, 2.64, 2.77, 3.29 (all m, 1H each, PC*H*CH_3), 6.05 (ddm, $^4J_{HP}$ = 7.9, $^3J_{HH}$ = 4.1, 1H, IrC_6H_4), 6.43 (m, 1H, IrC_6H_4), 6.55 (qq, $^3J_{HH}$ = 6.5, $^4J_{HH}$ = 1.7, 1H, C(CH_3)=C*H*CH_3), 6.61 (m, 2H, IrC_6H_4), 7.00-7.15 (m, 3H, CH), 7.48 (dm, $^3J_{HH}$ = 7.1, 1H, CH). ^{19}F NMR (C_6D_6): δ −78.80 (s). ^{31}P{^{1}H} NMR (C_6D_6): δ −19.19, 59.44 (both d, $^2J_{PP}$ = 298.9). ^{13}C{^{1}H} NMR data extracted from ^{1}H/^{13}C HMBC and HSQC spectra (C_6D_6): δ 1.45 ($SiCH_3$), 14.11 (C(CH_3)=CH*C*H_3), 15.00 (C(*C*H_3)=$CHCH_3$), 17.00, 17.51, 18.73, 18.90, 20.22, 20.38, 21.23 (PCH*C*H_3), 23.71, 24.40, 24.97, 25.19 (P*C*HCH_3), 120.61 (C, IrC_6H_4), 122.70 (CH, IrC_6H_4), 127.67, 128.03 (both CH), 128.60 (CH, IrC_6H_4), 129.73 (CH), 130.90 (CH, IrC_6H_4), 134.20 (CH), 137.40 (CH, IrC_6H_4), 137.60 (C(CH_3)=*C*HCH_3), 136.50 (*C*(CH_3)=$CHCH_3$), 147.30, 154.50, 154.70 (C). ^{29}Si{^{1}H} NMR (C_6D_6): δ 10.82 (dd, J_{SiP} ≈ $J_{SiP'}$ = 3.6).

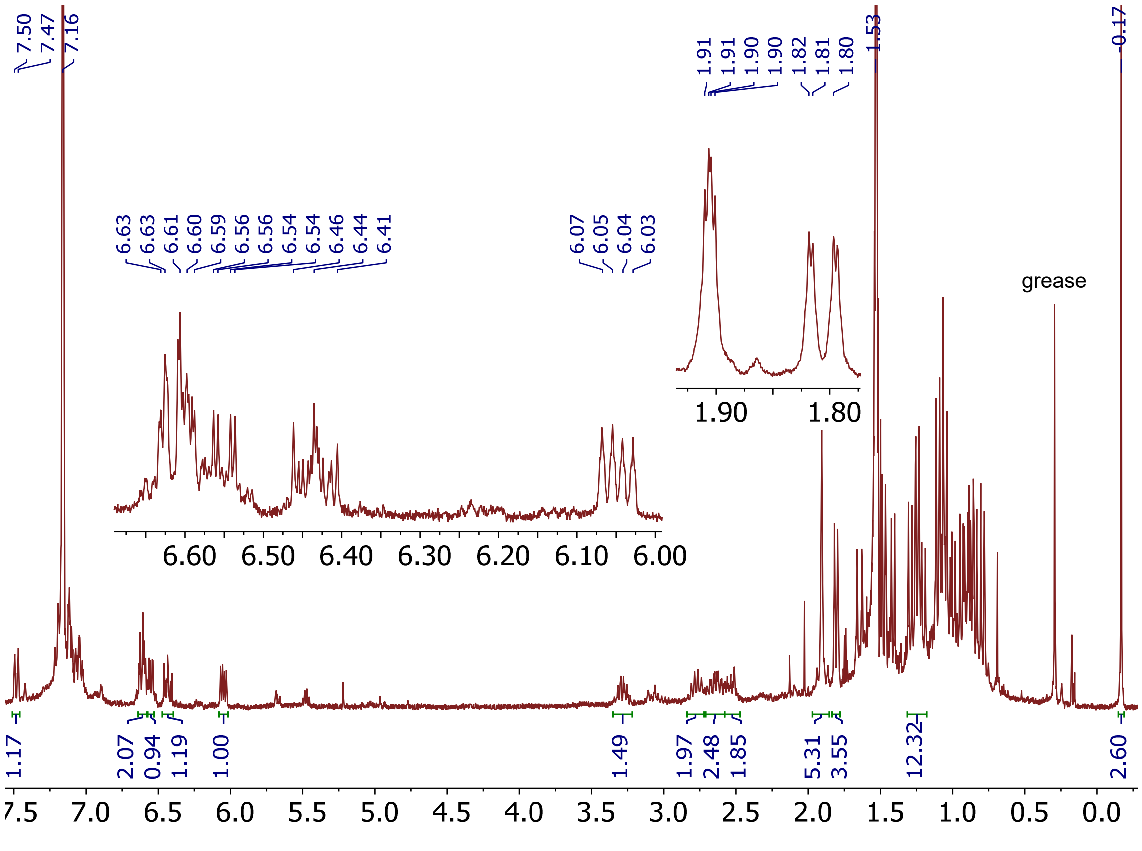

Figure S15. ^{1}H NMR spectrum of **3** in C_6D_6 at 298 K.

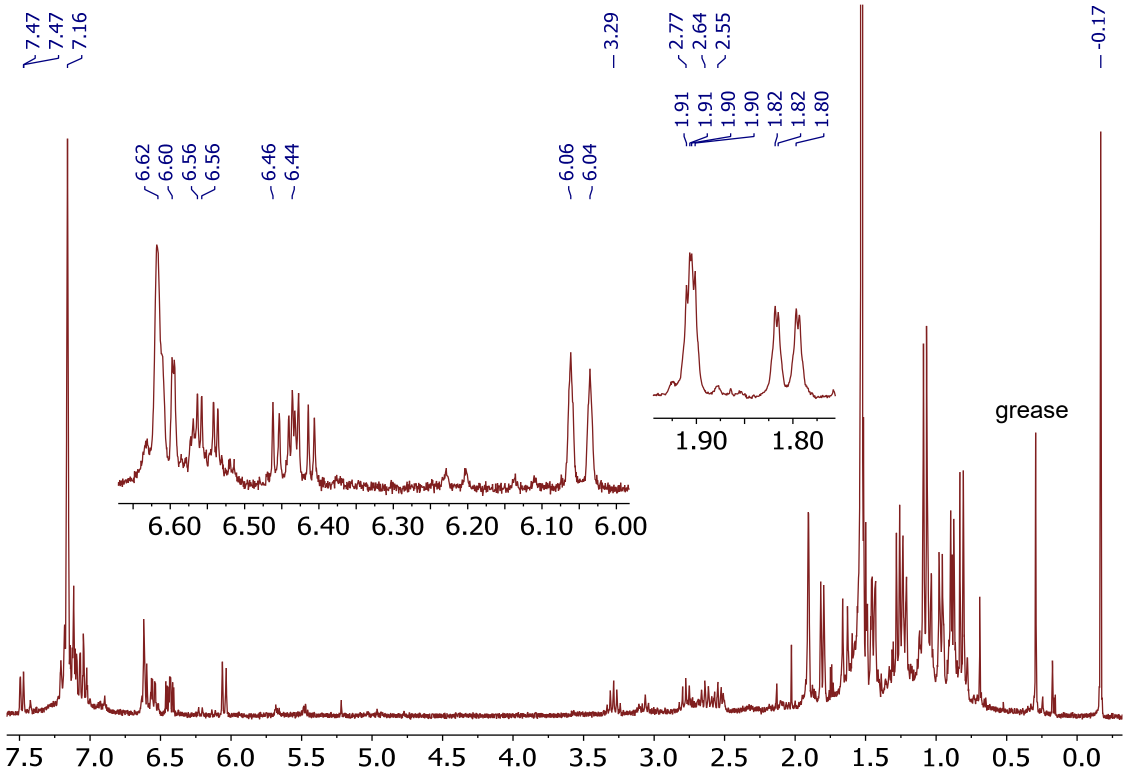

Figure S16. $^{1}H\{^{31}P\}$ NMR spectrum of **3** in C_6D_6 at 298 K.

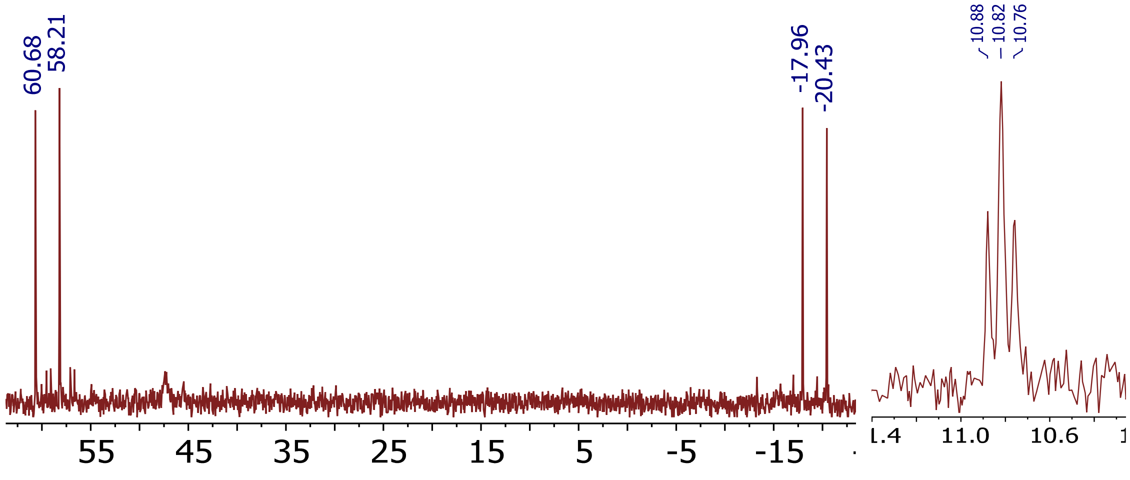

Figure S17. (left) $^{31}P\{^{1}H\}$ and (right) $^{29}Si\{^{1}H\}$ NMR spectra of **3** in C_6D_6 at 298 K.

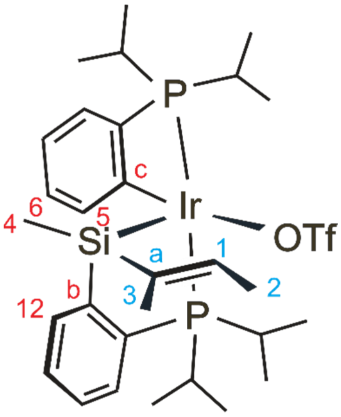

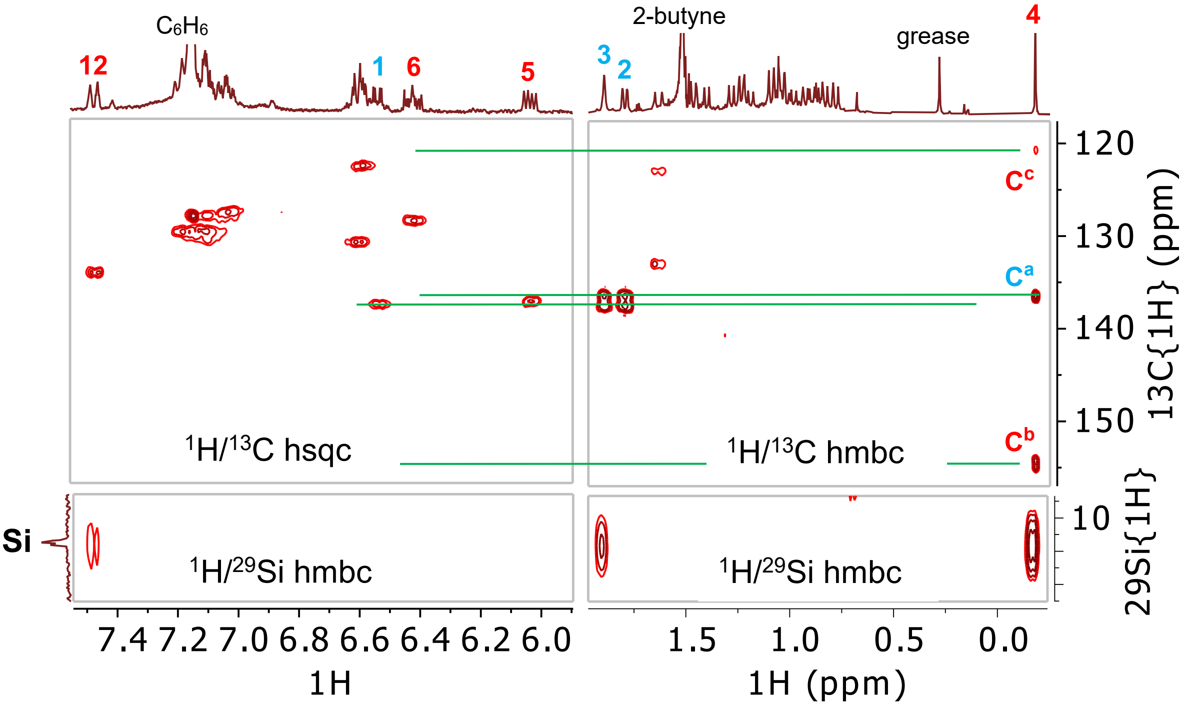

Figure S18. Selected sections of the $^{1}H/^{13}C$ HSQC and HMBC, and $^{1}H/^{29}Si$ HMBC correlation spectra of **3** in C_6D_6 at 298 K.

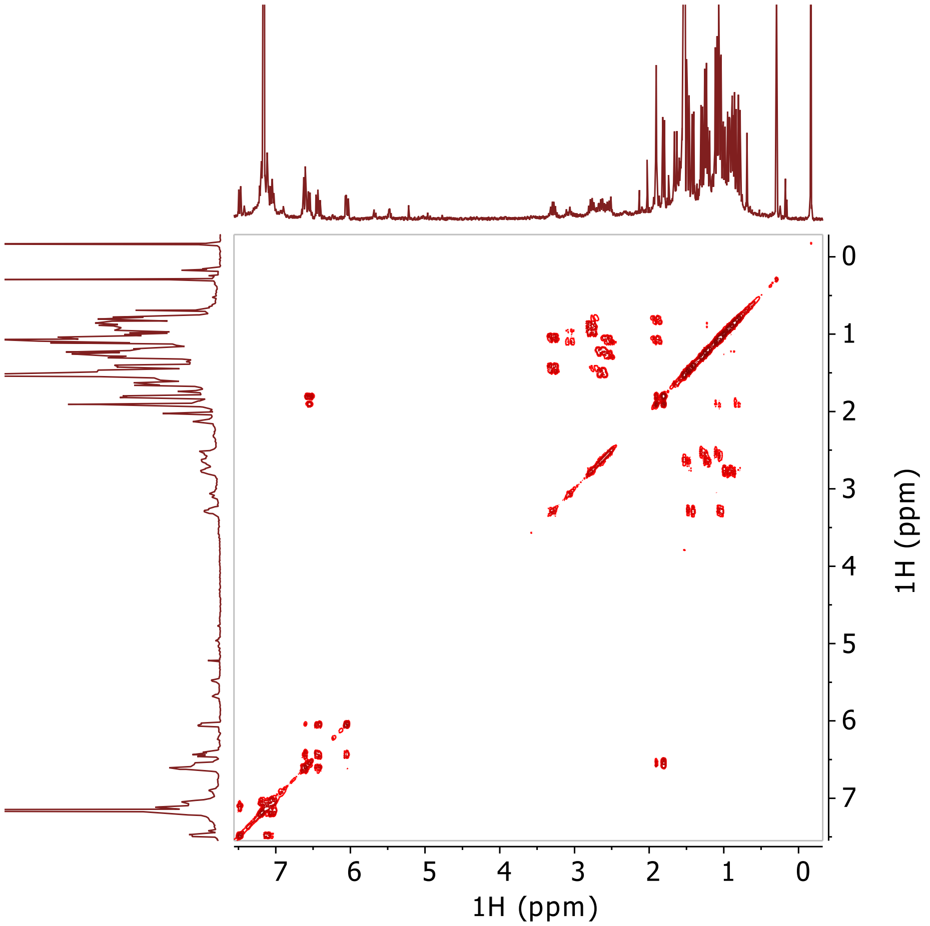

Figure S19. ^{1}H COSY NMR spectrum of complex **3** in C_6D_6 at 298 K.

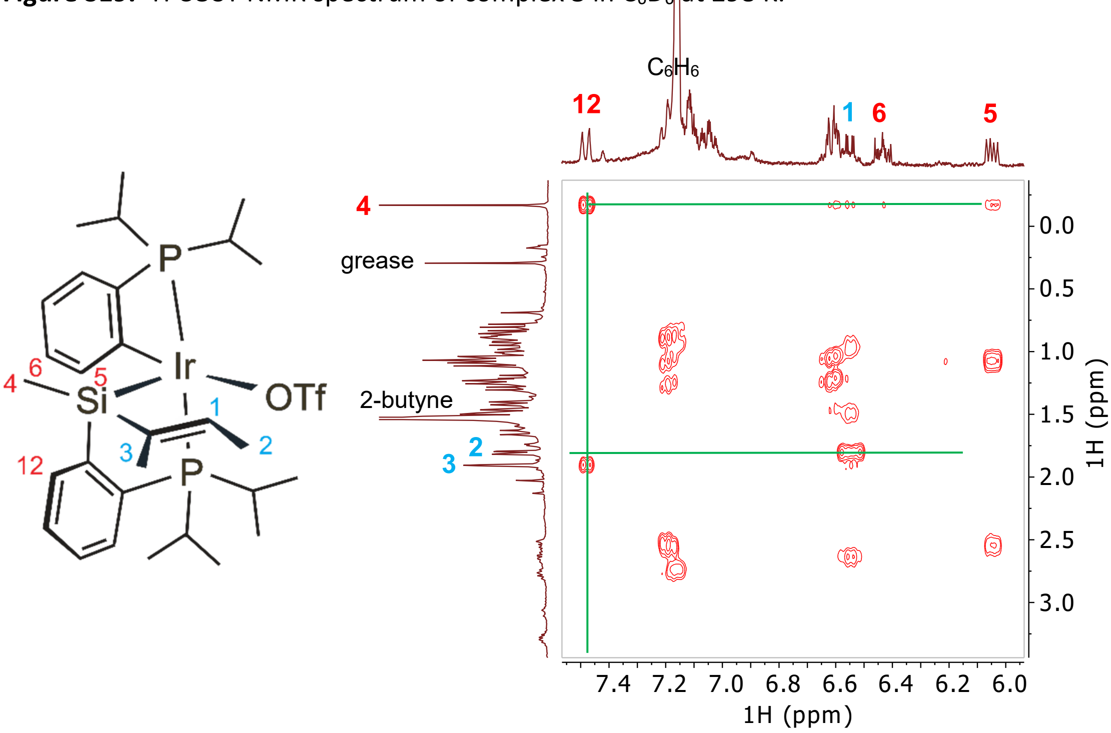

Figure S20. Selected region of the ^{1}H NOESY NMR spectrum of complex **3** in C_6D_6 at 298 K.

[Ir{κO,P-OSiMe(OH)(C_6H_4-2-P*i*Pr$_2$)}(κC,P-C_6H_4-2-P*i*Pr$_2$)(OH_2)$_2$](CF_3SO_3) (4): A solution of **1** (11.6 mg, 15 μmol) in CD_2Cl_2 (0.4 mL) at 253 K, was treated with 2 μL of water and 2-butyne (3 μL, 37 μmol). The NMR spectra revealed the formation of 2-butene together with complex **4** as the major organometallic product, accompanied by significant amounts of complexes **2** and **6** (see below) in a relative proportion 1:0.4:0.5, respectively. Partial data for **4**: ^{1}H NMR (CD_2Cl_2, 253 K): δ −0.15 (s, 3H, SiCH$_3$), 0.95 (m, 6H, PCHC*H*$_3$), 1.25 (m, 3H, PCHC*H*$_3$), 1.40 (m, 9H, PCHC*H*$_3$), 1.55 (m, 6H, PCHC*H*$_3$), 1.96 (br, 2H, OH$_2$), 2.61-2.71 (m, 2H, PC*H*CH$_3$), 291-3.04 (m, 2H, PC*H*CH$_3$), 4.45 (s, 1H, OH), 6.12 (ddm, $^3J_{HH}$ = 7.8, $^4J_{HP}$ = 4.0, 1H, IrC$_6$H$_4$), 6.70 (ddd, $^3J_{HH}$ = 7.8, 7.7, $^4J_{HH}$ = 1.5, 1H, IrC$_6$H$_4$), 6.89 (dddd, $^3J_{HH}$ = 7.7, 7.5, $^4J_{HH}$ = 0.8, $^4J_{HP}$ = 2.6, 1H, IrC$_6$H$_4$), 7.01 (ddd, $^3J_{HH}$ = 7.5, $^4J_{HH}$ = 1.1, $^3J_{HP}$ = 8.2, 1H, IrC$_6$H$_4$), 8.13 (dm, $^3J_{HH}$ = 7.4, 1H, CH). ^{19}F NMR (CD_2Cl_2): δ −78.60 (s). ^{31}P{^{1}H} NMR (CD_2Cl_2, 253 K): δ −18.19, 58.01 (both d, $^2J_{PP}$ = 290.7). ^{13}C{^{1}H} NMR (CD_2Cl_2, 253 K): δ 3.87 (SiCH$_3$), 23.64 (d, $^1J_{CP}$ = 19.4, P*C*HCH$_3$), 24.60 (d, $^1J_{CP}$ = 26.5, P*C*HCH$_3$), 25.11 (d, $^1J_{CP}$ = 28.2, P*C*HCH$_3$), 25.92 (dd, $^1J_{CP}$ = 19.4, $^3J_{CP}$ = 4.4, P*C*HCH$_3$), 121.36 (dd, $^2J_{CP}$ = 6.0, 3.7, C, IrC$_6$H$_4$), 123.67 (d, J_{CP} = 7.8, CH, IrC$_6$H$_4$), 129.36 (CH, IrC$_6$H$_4$), 131.71 (CH, IrC$_6$H$_4$), 136.70 (dd, $^3J_{CP}$ = 14.8, 3.2, CH, IrC$_6$H$_4$), 137.60 (d, $^1J_{CP}$ = 50.8, C), 147.05 (d, $^2J_{CP}$ = 40.5, C), 153.84 (d, $^2J_{CP}$ = 45.4, C). ^{29}Si{^{1}H} NMR (CD_2Cl_2, 253 K): δ 6.74 (dd, $J_{SiP} \approx J_{SiP'}$ = 3.7). The crystals used in the X-ray diffraction experiment were obtained from a dichloromethane solution stored at 253 K.

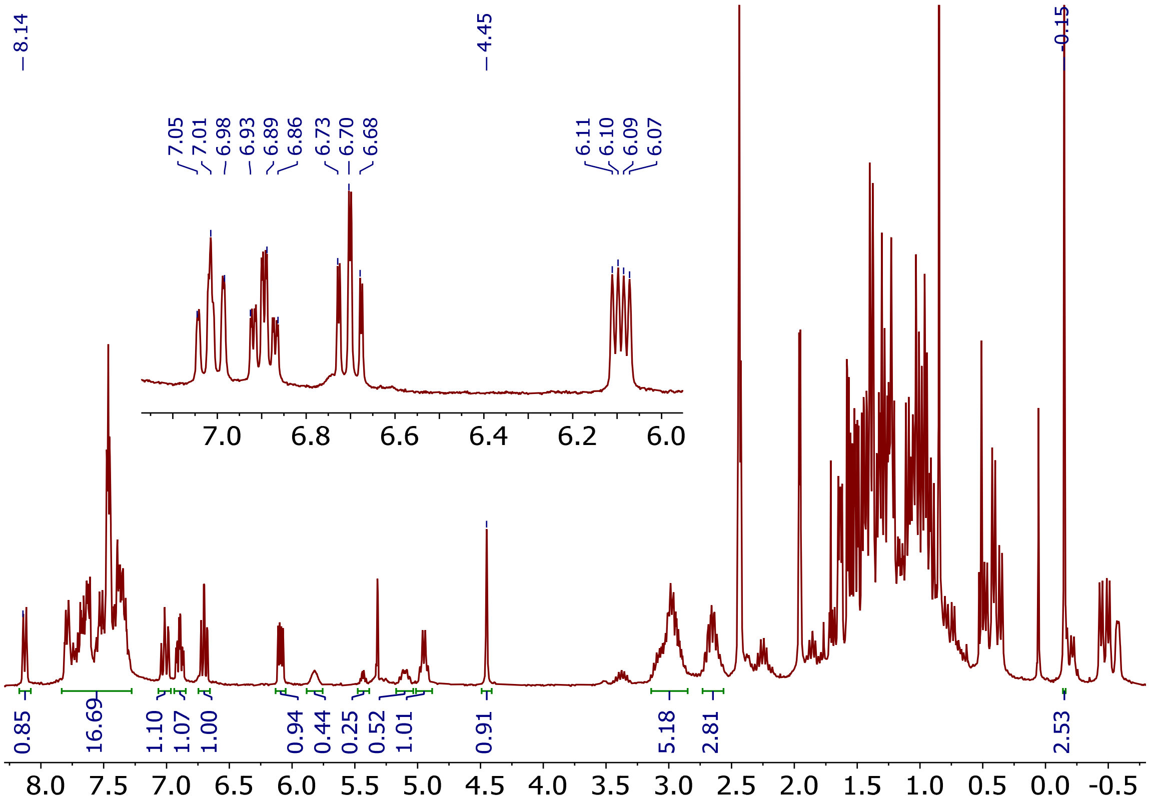

Figure S21. ^{1}H NMR spectrum of **4** in CD_2Cl_2 at 253 K.

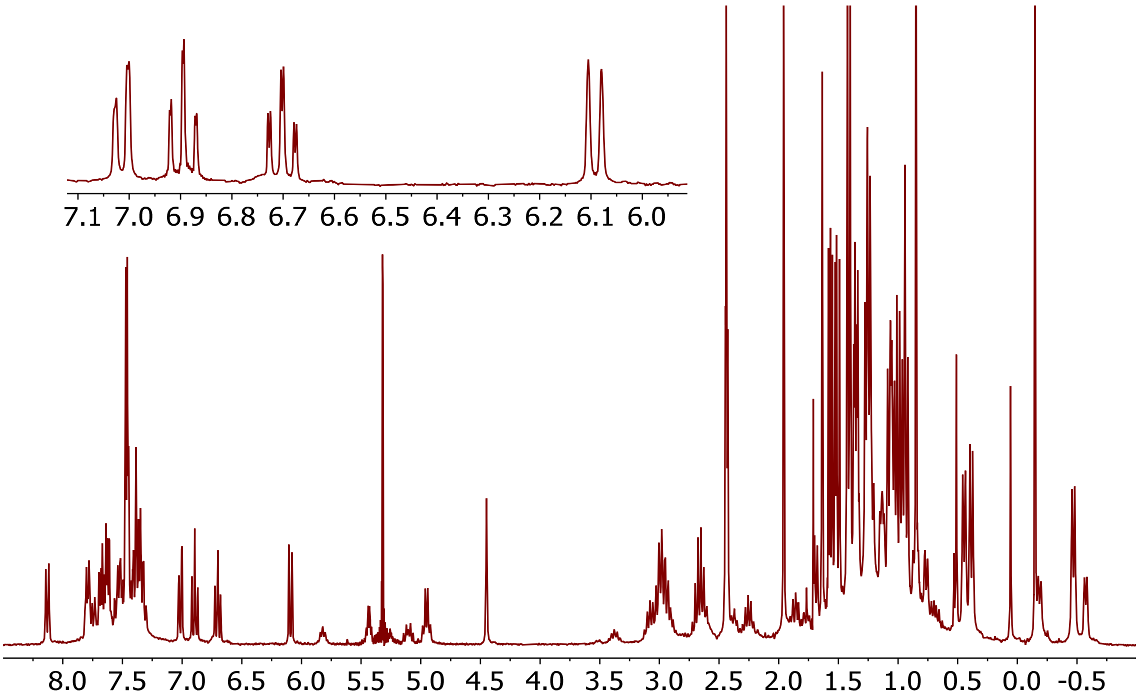

Figure S22. $^{1}H\{^{31}P\}$ NMR spectrum of **4** in CD_2Cl_2 at 253 K.

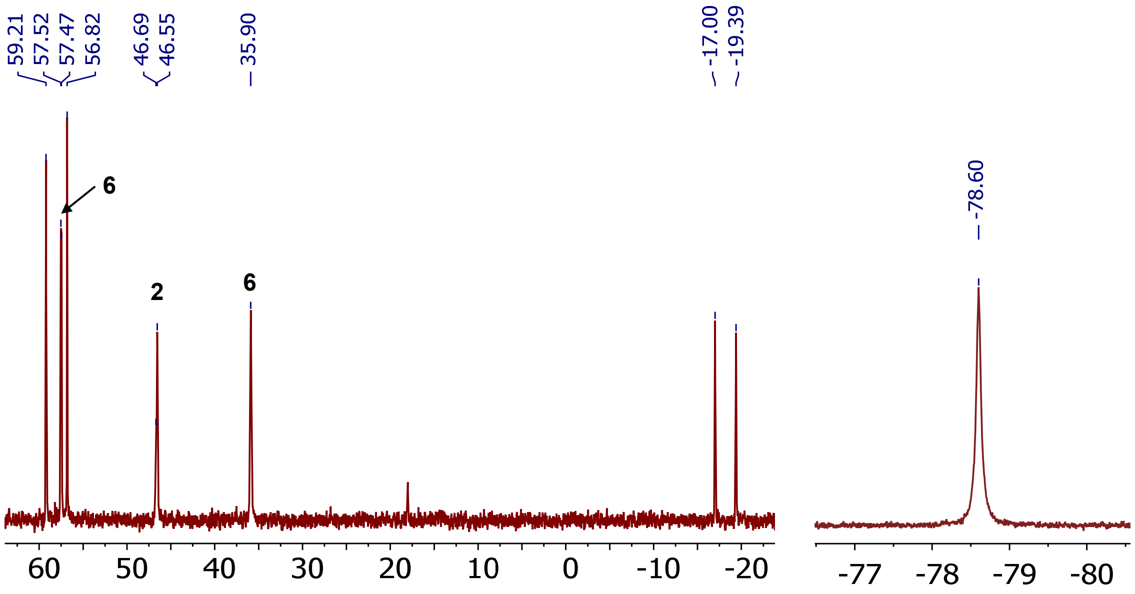

Figure S23. NMR spectra of **4** in CD_2Cl_2, (left) $^{31}P\{^{1}H\}$ at 253 K, and (right) ^{19}F at 298 K.

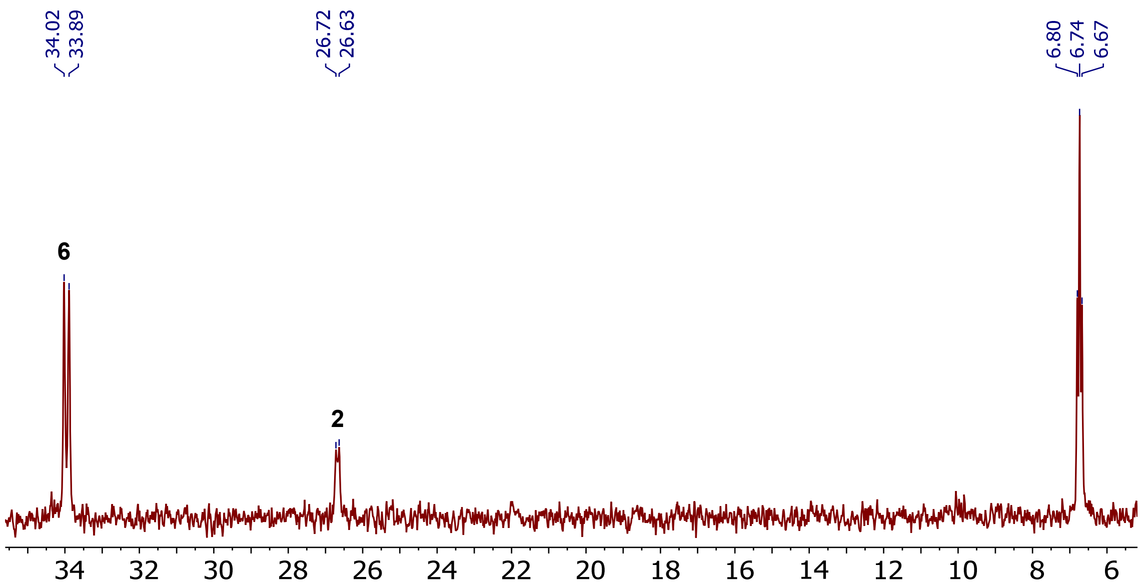

Figure S24. $^{29}Si\{^1H\}$ NMR spectrum of **4** in CD_2Cl_2 at 253 K.

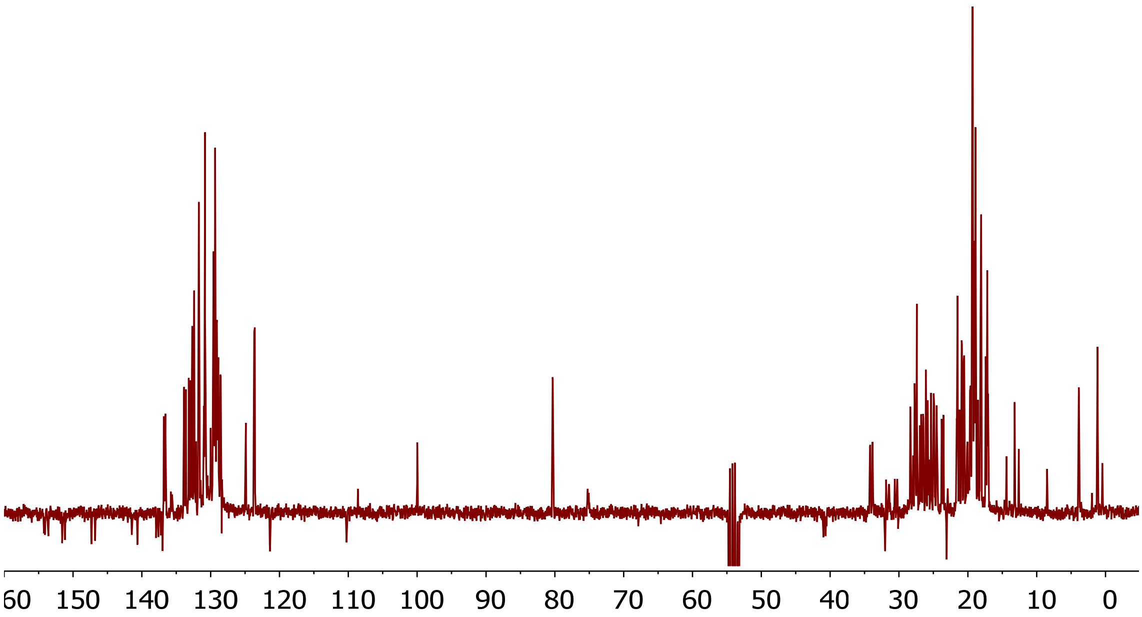

Figure S25. $^{13}C\{^1H\}$-apt NMR spectrum of **4** in CD_2Cl_2 at 253 K.

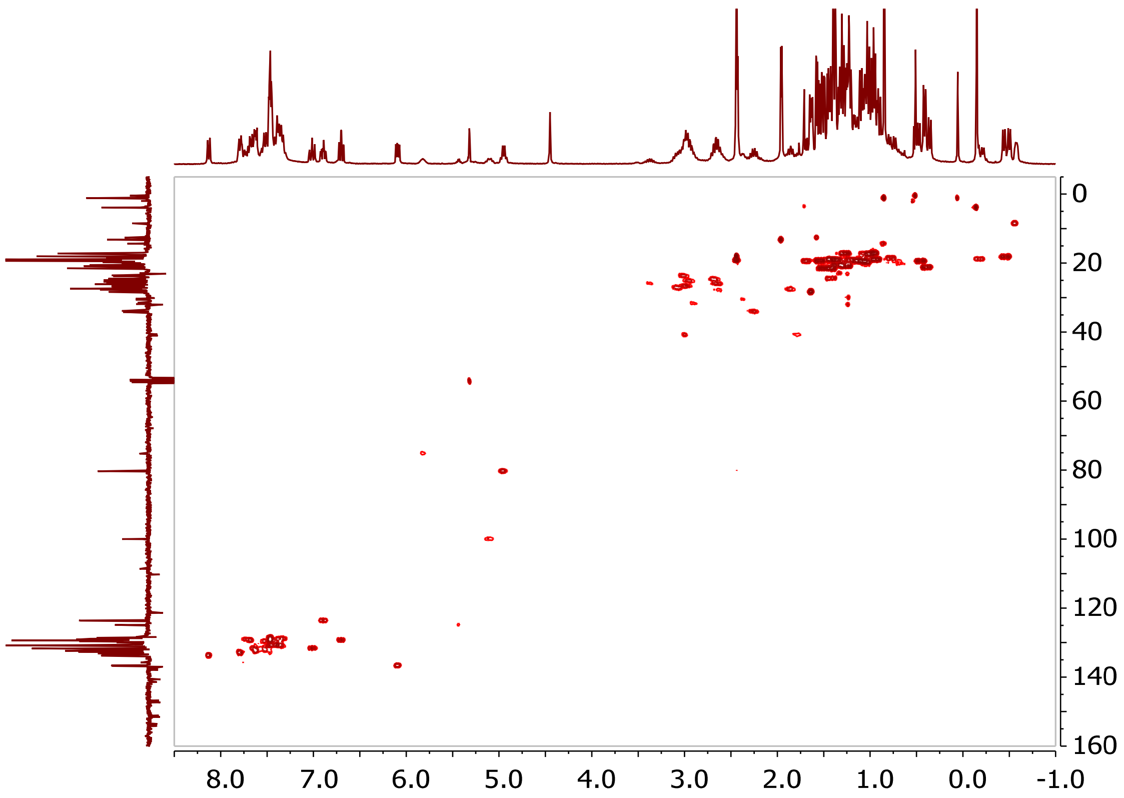

Figure S26. $^{1}H/^{13}C$ HSQC NMR spectrum of **4** in CD_2Cl_2 at 253 K.

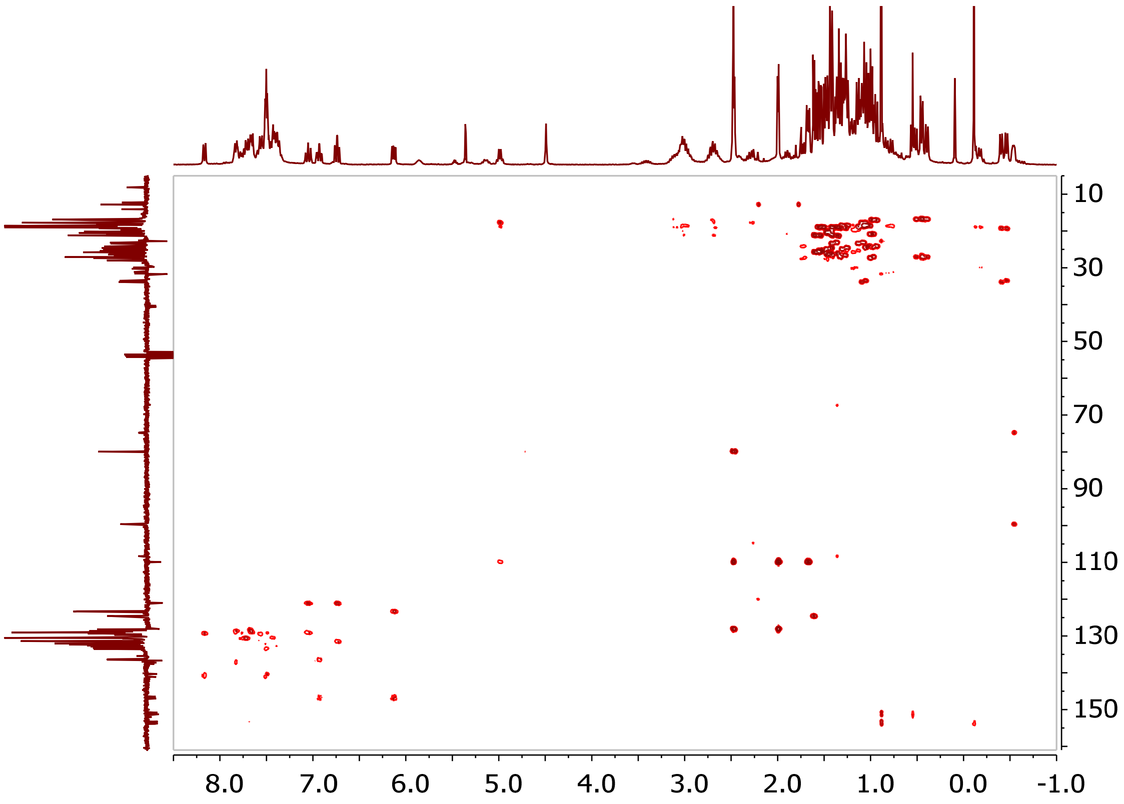

Figure S27. $^{1}H/^{13}C$ HMBC NMR spectrum of **4** in CD_2Cl_2 at 253 K.

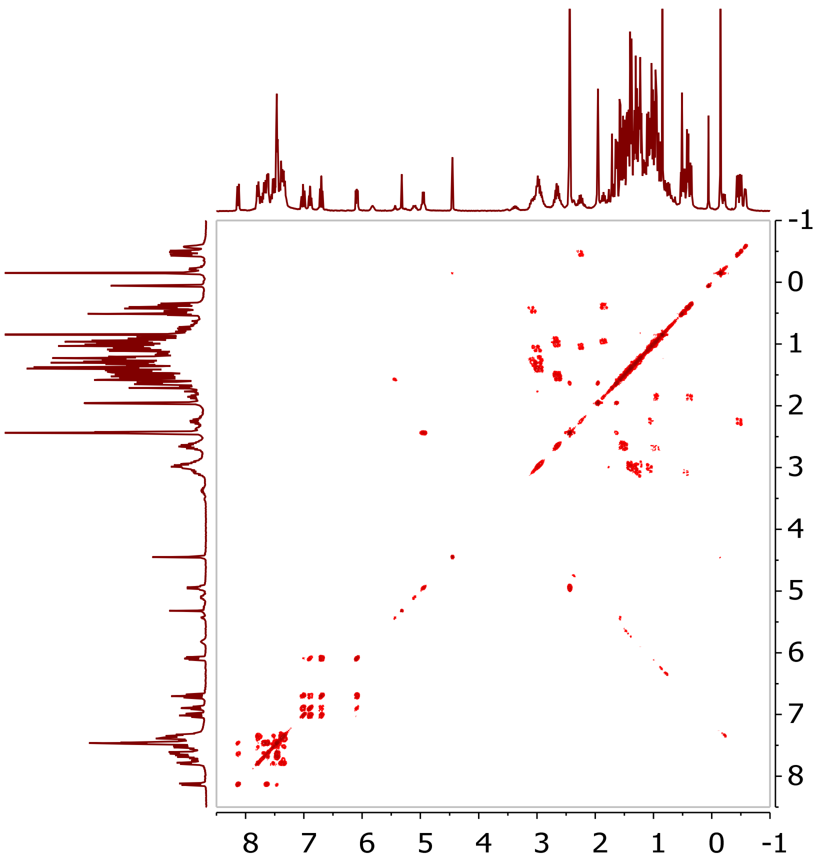

Figure S28. ^{1}H COSY NMR spectrum of **4** in CD_2Cl_2 at 253 K.

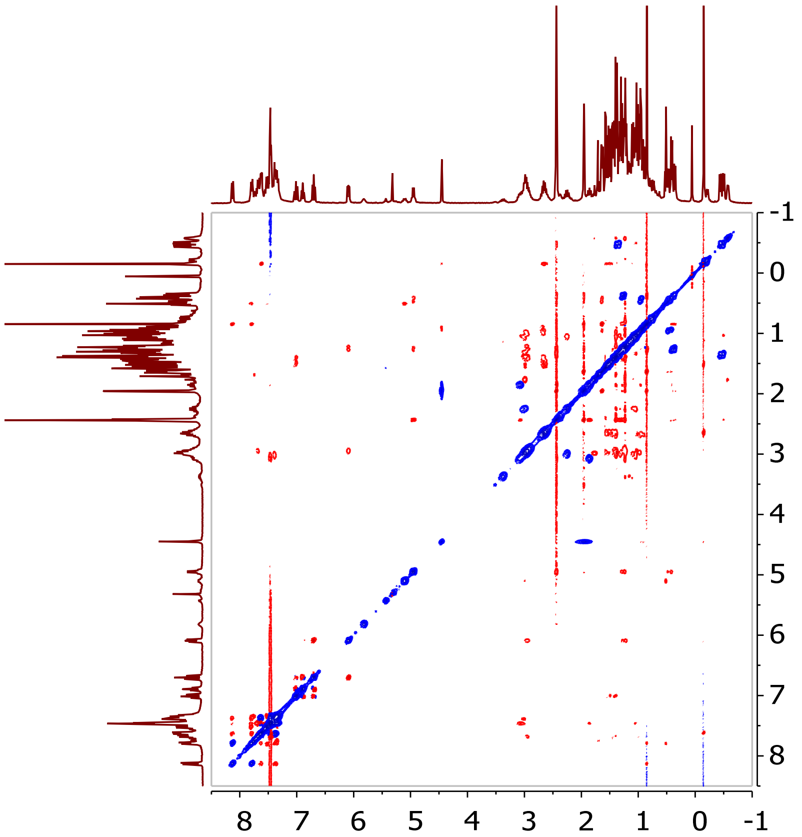

Figure S29. ^{1}H NOESY NMR spectrum of **4** in CD_2Cl_2 at 253 K.

[IrH{κP,P,Si-SiMe(C_6H_4-2-P*i*Pr_2)$_2$}{η^2-CMe≡CMe}](CF_3SO_3) (5): A solution of **1** (21.1 mg, 27 μmol) in CD_2Cl_2 (0.4 mL) cooled at 233 K was treated with 2-butyne (8 μL, 100 μmol). The NMR spectra indicated the formation of **5** as the main reaction product. ^{1}H NMR (CD_2Cl_2, 233 K): δ −14.44 (t, $^2J_{HP}$ = 16.5, 1H, IrH), 0.73 (s, 3H, $SiCH_3$), 0.79 (dvt, $^{3,5}N$ = 19.0, $^3J_{HH}$ = 10.0, 6H, PCHCH_3), 0.90 (dvt, $^{3,5}N$ = 18.0, $^3J_{HH}$ = 10.4, 6H, PCHCH_3), 1.11 (dvt, $^{3,5}N$ = 18.3, $^3J_{HH}$ = 10.0, 6H, PCHCH_3), 1.19 (dvt, $^{3,5}N$ = 15.8, $^3J_{HH}$ = 6.8, 6H, PCHCH_3), 2.83 (s, 6H, C≡CH_3), 3.13, 3.23 (both m, 2H each, PC*H*CH_3), 7.44-7.59 (m, 4H, CH), 8.08 (d, J_{HH} = 7.4, 2H, CH). ^{19}F NMR (CD_2Cl_2, 253 K): δ −78.94 (s). ^{31}P{^{1}H} NMR (CD_2Cl_2, 233 K): δ 64.22 (s).

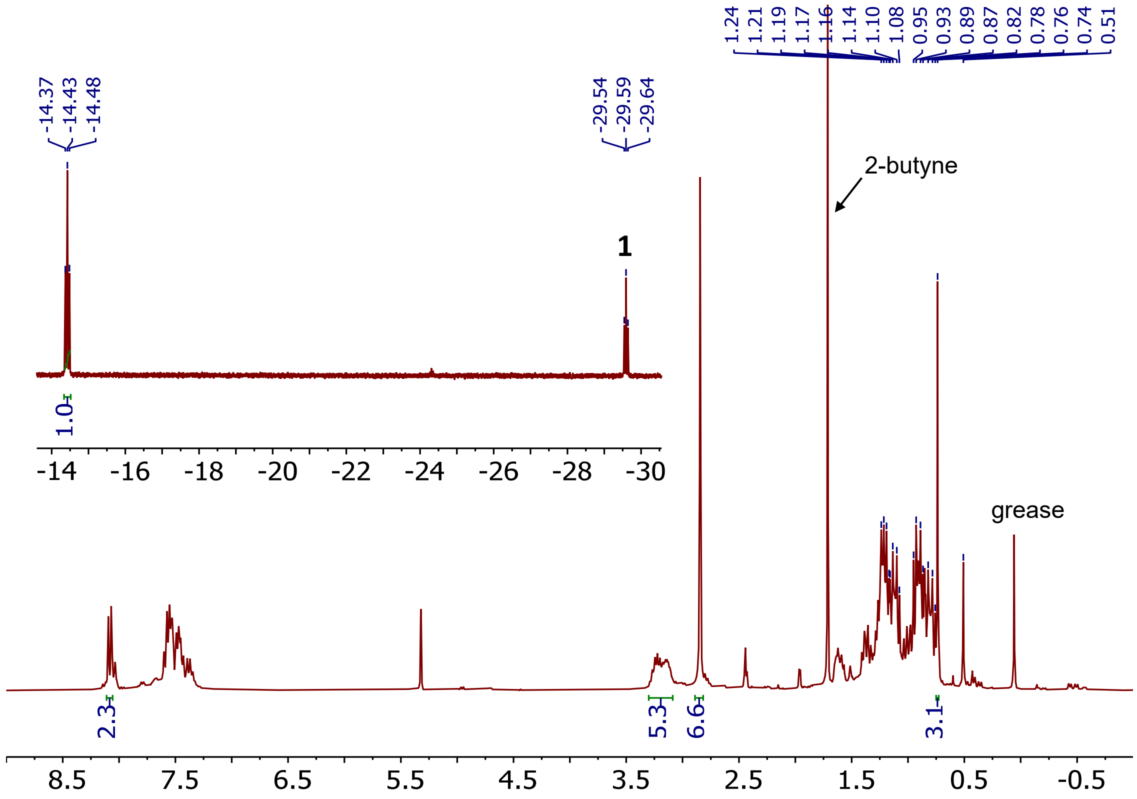

Figure S30. ^{1}H NMR spectrum of **5** in CD_2Cl_2 at 253 K.

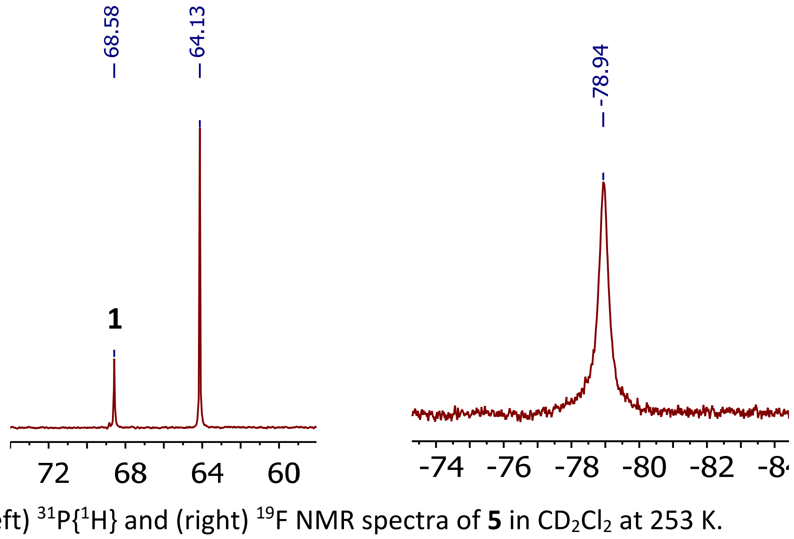

Figure S31. (left) ^{31}P{^{1}H} and (right) ^{19}F NMR spectra of **5** in CD_2Cl_2 at 253 K.

[Ir{κC,η^3-CMeCMeCMeCHMe}{κP,P,Si-SiMe(C_6H_4-2-P*i*Pr_2)$_2$}](CF_3SO_3) (6): A solution of **1** (23.1 mg, 30 μmol) in CD_2Cl_2 (0.4 mL) at 195 K was treated with 2-butyne (10 μL, 120 μmol) and stored at 253 K for 24 h. The NMR spectra at 253 K indicated the selective formation of a new compound, **6,** which was characterized by NMR after evacuation of the 2-butyne excess in a stream of argon. ^{1}H NMR (CD_2Cl_2, 253 K): δ −0.47 (dd, $^3J_{HP}$ = 17.2, $^3J_{HH}$ = 7.0, 3H, PCHCH_3), 0.38 (dd, $^3J_{HP}$ = 16.5, $^3J_{HH}$ = 7.2, 3H, PCHCH_3), 0.44 (dd, $^3J_{HP}$ = 18.6, $^3J_{HH}$ = 6.6, 3H, PCHCH_3), 0.85 (s, 3H, SiCH_3), 0.95 (dd, $^3J_{HP}$ = 10.9, $^3J_{HH}$ = 6.7, 3H, PCHCH_3), 1.04 (dd, $^3J_{HP}$ = 13.1, $^3J_{HH}$ = 6.8, 3H, PCHCH_3), 1.07 (dd, $^3J_{HP}$ = 16.5, $^3J_{HH}$ = 7.0, 3H, PCHCH_3), 1.26 (dd, $^3J_{HP}$ = 16.3, $^3J_{HH}$ = 7.0, 3H, PCHCH_3), 1.36 (dd, $^3J_{HP}$ = 15.8, $^3J_{HH}$ = 7.0, 3H, PCHCH_3), 1.63 (dd, $^4J_{HP}$ = 5.6, $^4J_{HP'}$ = 3.3, 3H, CCH_3), 1.85 (m, 1H, PC*H*CH_3), 1.96 (d, $^4J_{HP}$ = 2.9, 3H, CCH_3), 2.26 (m, 1H, PC*H*CH_3), 2.43 (d, $^3J_{HH}$ = 6.4, 3H, CHCH_3), 2.44 (s, 3H, CCH_3), 3.00, 3.08 (both m, 1H each, PC*H*CH_3), 4.95 (q, $^3J_{HH}$ = 6.4, 1H, C*H*CH_3), 7.33, 7.37, 7.40 (all m, 1H each, CH), 7.46 (m, 2H, CH), 7.63 (m, 1H, CH), 7.79 (d, J_{HH} = 7.0, 1H. CH), 8.13 (d, J_{HH} = 7.3, 1H, CH). ^{19}F NMR (CD_2Cl_2, 253 K): δ −79.04 (s). ^{31}P{^{1}H} NMR (CD_2Cl_2, 253 K): δ 57.45, 35.74 (both d, $^2J_{PP}$ = 8.1). ^{13}C{^{1}H} NMR (CD_2Cl_2, 253 K): δ 1.09 (s, SiCH_3), 13.13 (d, $^3J_{CP}$ = 3.7, *C*CH_3), 17.03 (d, $^2J_{CP}$ = 6.4, PCH*C*H_3), 17.12, (s, PCH*C*H_3), 18.02 (s, C*C*H_3), 18.08 (s, PCH*C*H_3), 19.00 (d, $^2J_{CP}$ = 4.7, PCH*C*H_3), 19.13 (s, CH*C*H_3), 19.28 (d, $^2J_{CP}$ = 4.8, PCH*C*H_3), 19.34 (s, PCH*C*H_3), 19.60 (d, $^2J_{CP}$ = 6.6, PCH*C*H_3), 21.14 (d, $^2J_{CP}$ = 4.2, PCH*C*H_3), 23.56 (d, $^1J_{CP}$ = 19.0, P*C*HCH_3), 27.14 (dd, $^1J_{CP}$ = 35.5, $^3J_{CP}$ = 1.4, P*C*HCH_3), 27.49 (d, $^1J_{CP}$ = 25.0, P*C*HCH_3), 28.32 (d, $^4J_{CP}$ = 5.0, C*C*H_3), 33.95 (dd, $^1J_{CP}$ = 28.6, $^3J_{CP}$ = 3.7, P*C*HCH_3), 80.12 (s, *C*HCH_3), 110.14 (dd, $^2J_{CP}$ = $^2J_{CP'}$ = 4.7, *C*CH_3), 121.06 (q, $^1J_{CF}$ = 320.9, CF_3SO_3), 128.31 (d, $^2J_{CP}$ = 4.0, *C*CH_3), 128.51 (d, J_{CP} = 6.2, CH), 128.87 (d, J_{CP} = 5.8, CH), 129.49 (d, J_{CP} = 7.6, CH), 130.65 (d, J_{CP} = 2.0, CH), 130.78 (d, J_{CP} = 3.0, CH), 131.70 (d, J_{CP} = 2.5, CH), 133.00 (d, J_{CP} = 18.2, CH), 133.67 (d, J_{CP} = 18.2, CH), 137.24 (d, $^1J_{CP}$ = 44.4, C), 140.99 (d, $^1J_{CP}$ = 61.3, C), 151.29 (d, $^2J_{CP}$ = 31.9, C), 153.75 (d, $^2J_{CP}$ = 38.8, C), 239.85 (d, J_{CP} = 65.6, Ir=*C*CH_3). ^{29}Si{^{1}H} NMR (CD_2Cl_2, 253 K): δ 33.90 (dd, J_{SiP} = 8.1, $J_{SiP'}$ < linewidth).

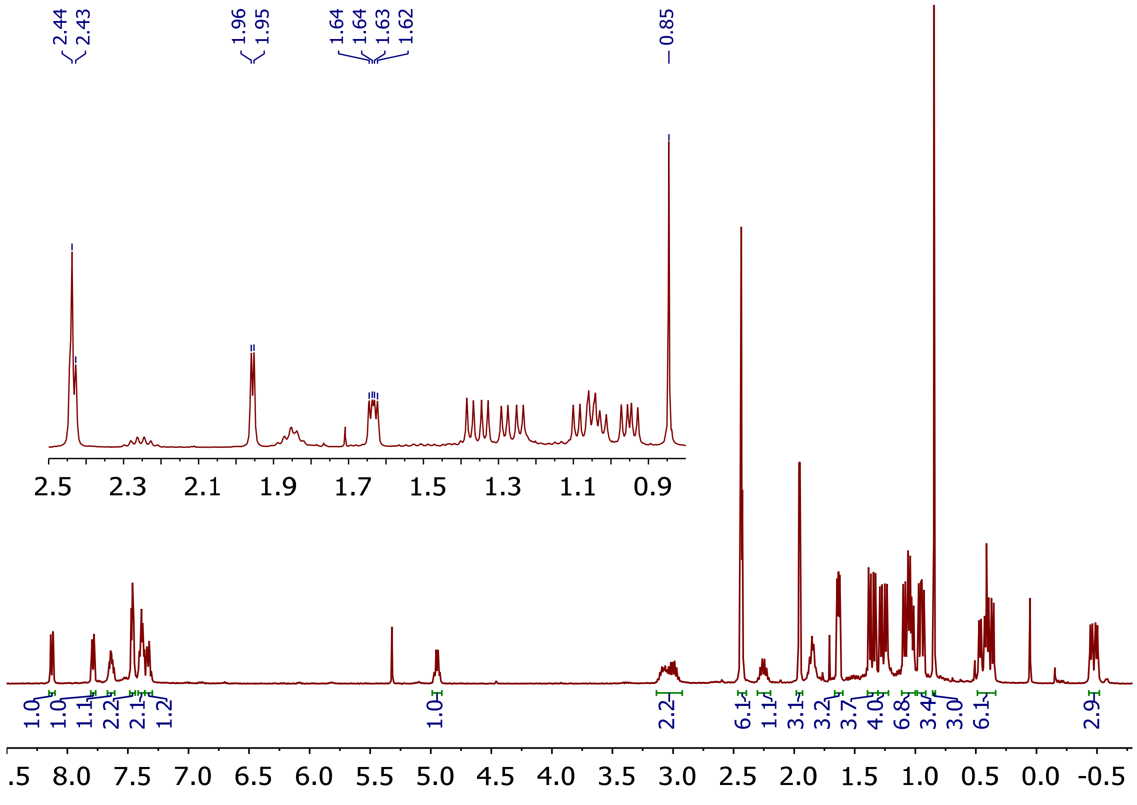

Figure S32. ^{1}H NMR spectrum of **6** in CD_2Cl_2 at 253 K.

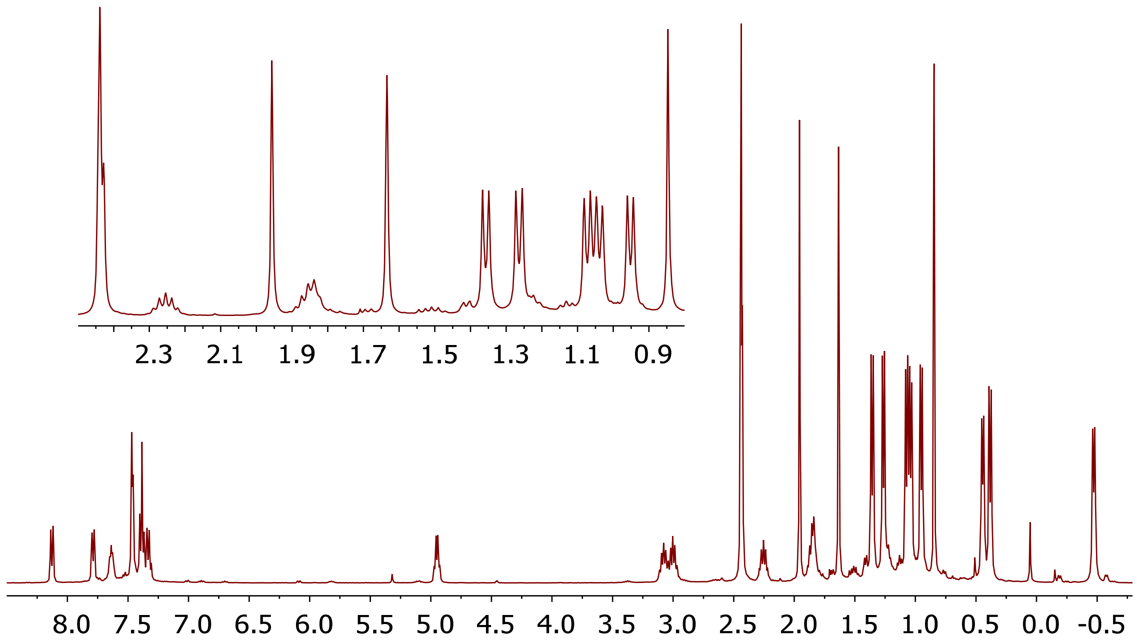

Figure S33. $^{1}H\{^{31}P\}$ NMR spectrum of **6** in CD_2Cl_2 at 253 K.

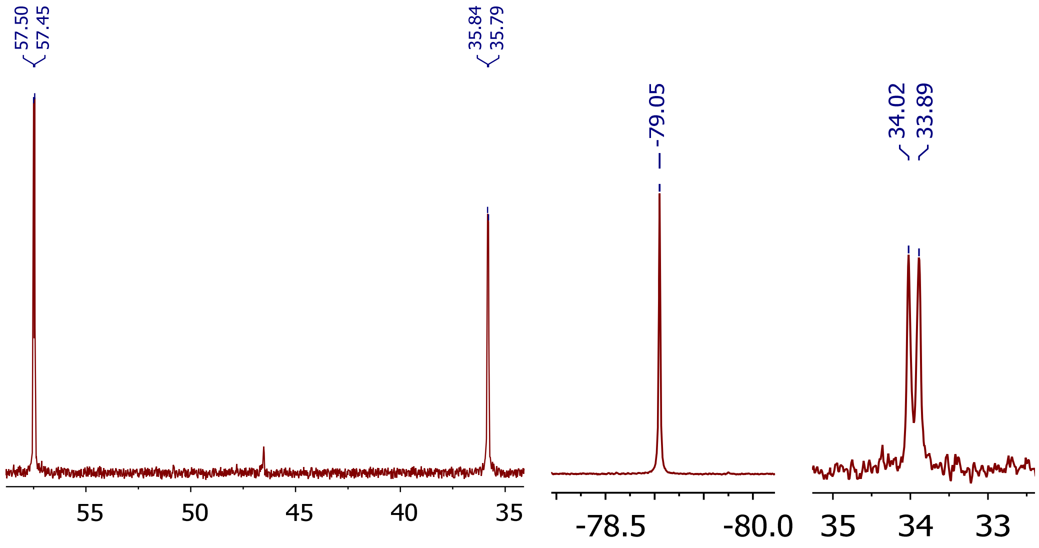

Figure S34. (left) $^{31}P\{^{1}H\}$, (center) ^{19}F, and (right) $^{29}Si\{^{1}H\}$ NMR spectra of **6** in CD_2Cl_2 at 253 K.

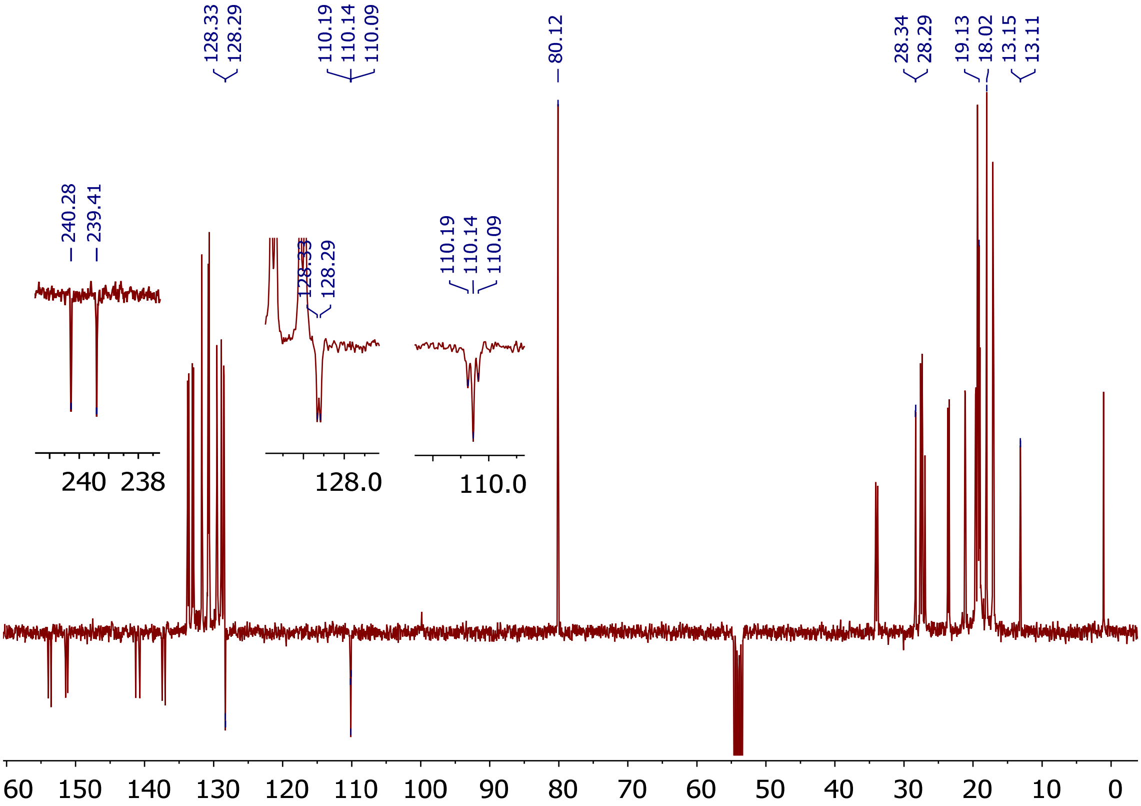

Figure S35. $^{13}C\{^{1}H\}$ APT NMR spectrum of **6** in CD_2Cl_2 at 253 K.

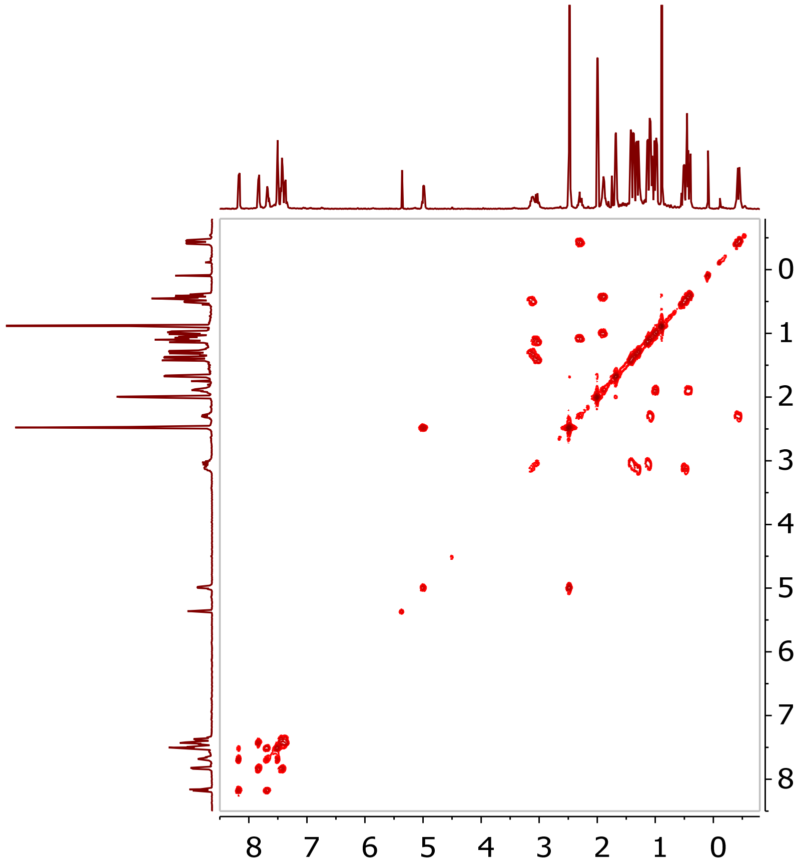

Figure S36. ^{1}H COSY NMR spectrum of **6** in CD_2Cl_2 at 253 K.

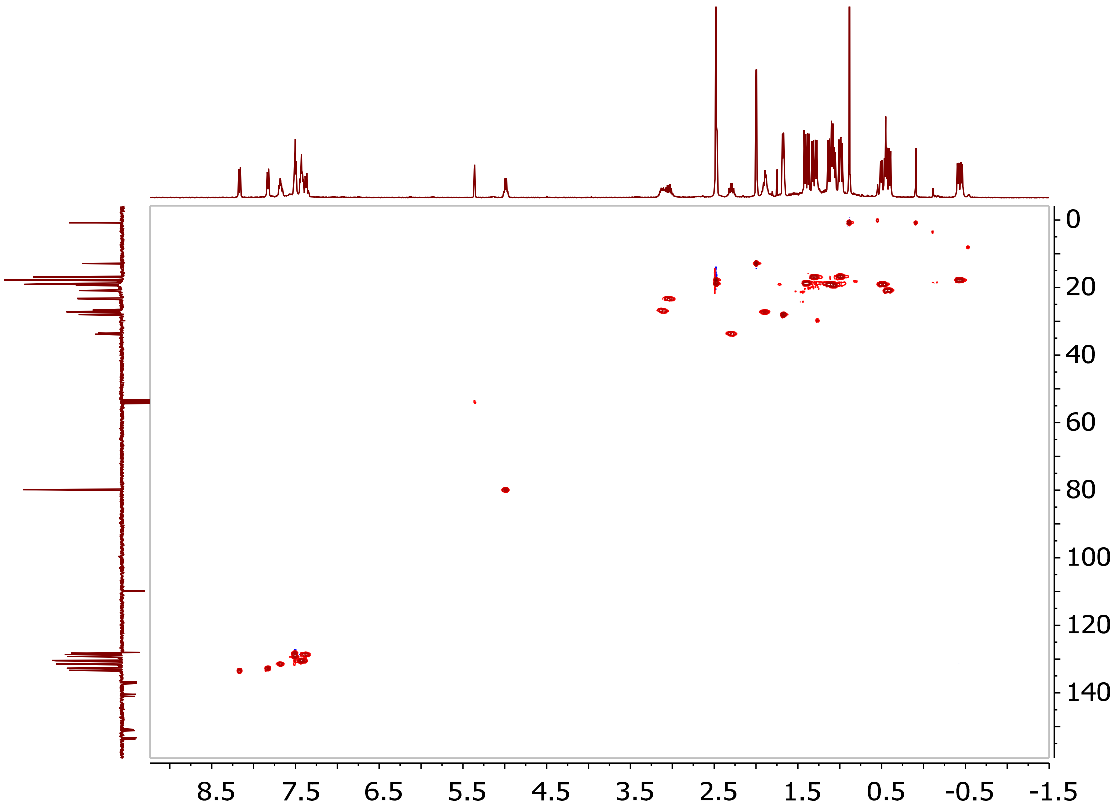

Figure S37. $^{1}H/^{13}C$ HSQC NMR spectrum of **6** in CD_2Cl_2 at 253 K.

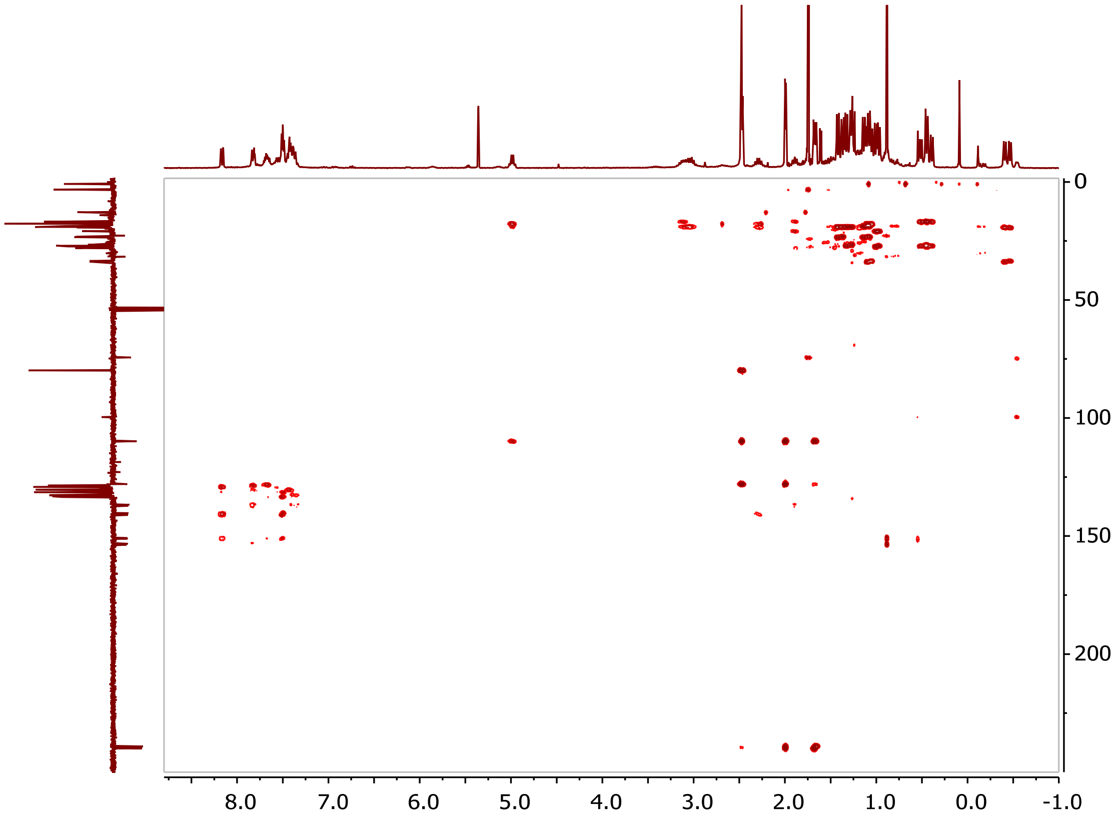

Figure S38. $^{1}H/^{13}C$ HMBC NMR spectrum of **6** in CD_2Cl_2 at 253K.

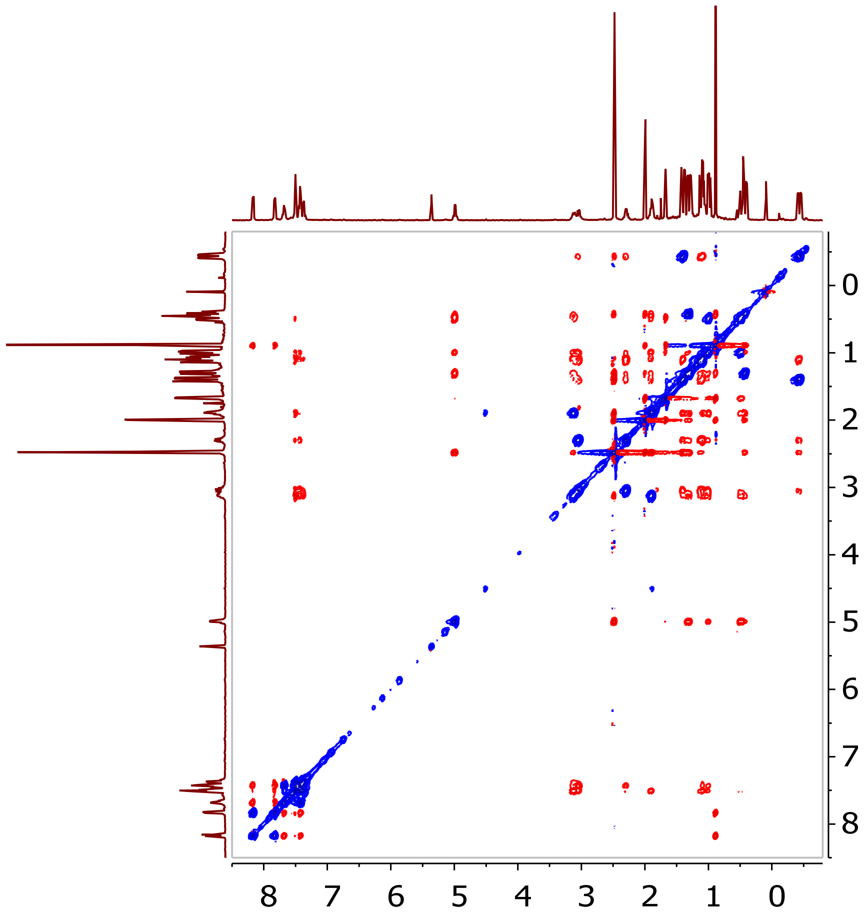

Figure S39. ^{1}H NOESY NMR spectrum of **6** in CD_2Cl_2 at 253 K.

[Ir{η^{3}-CH_2C(*Z*-CMe=CHMe)CHMe}{κP,P,Si-SiMe(C_6H_4-2-P*i*Pr_2)$_2$}](CF_3SO_3) (7): After evacuation of the 2-butyne excess, the previously described solution containing **6** was kept at room temperature for three days. The NMR spectra indicated the formation of compound **7** as the major evolution product. ^{1}H NMR (CD_2Cl_2): δ −0.38 (brdd, J_{HH} = 5.2, J_{HP} = 3.1, 3H, CHC*H*$_3$), 0.40 (s, 3H, $SiCH_3$), 0.58 (dd, $^{3}J_{HP}$ = 14.2, $^{3}J_{HH}$ = 6.9, 3H, PCHC*H*$_3$), 1.08 (dd, $^{3}J_{HP}$ = 15.6, $^{3}J_{HH}$ = 7.0, 3H, PCHC*H*$_3$), 1.11 (dd, $^{3}J_{HP}$ = 13.0, $^{3}J_{HH}$ = 7.2, 3H, PCHC*H*$_3$), 1.19 (dd, $^{3}J_{HP}$ = 15.5, $^{3}J_{HH}$ = 7.0, 3H, PCHC*H*$_3$), 1.28-1.37 (m, 12H, PCHC*H*$_3$), 1.54 (s, 3H, CCH_3), 1.57 (m, 1H, CH_2), 1.74 (d, J_{HH} = 7.0, 3H, CHC*H*$_3$), 2.27, 2.36, 3.10, 3.27 (all m, 1H each, PC*H*CH_3), 3.48 (m, 1H, CH_2), 5.84 (m, 1H, C*H*CH_3), 6.21 (q, J_{HH} = 7.0, 1H, C*H*CH_3), 7.37, 7.45 (both m, 2H each, CH), 7.51 (m, 1H, CH), 7.64 (m, 3H, CH). ^{19}F NMR (CD_2Cl_2): δ −78.79 (s). ^{31}P{^{1}H} NMR (CD_2Cl_2): δ 47.42, 41.90 (both br). ^{13}C{^{1}H} NMR (CD_2Cl_2): δ −1.24 (s, $SiCH_3$), 9.03 (d, $^{3}J_{CP}$ = 3.1, CH*C*H$_3$), 13.89 (s, C*C*H$_3$), 15.11 (s, CH*C*H$_3$), 19.80 (d, $^{2}J_{CP}$ = 2.9, PCH*C*H$_3$), 19.98 (d, $^{2}J_{CP}$ = 2.3, PCH*C*H$_3$), 20.00 (d, $^{2}J_{CP}$ = 1.3, PCH*C*H$_3$), 20.14 (d, $^{2}J_{CP}$ = 0.8, PCH*C*H$_3$), 20.15 (d, $^{2}J_{CP}$ = 3.6, PCH*C*H$_3$), 20.58 (d, $^{2}J_{CP}$ = 2.9, PCH*C*H$_3$), 21.97 (s, PCH*C*H$_3$), 23.19 (d, $^{2}J_{CP}$ = 2.3, PCH*C*H$_3$), 27.96 (dd, $^{1}J_{CP}$ = 32.4, $^{3}J_{CP}$ = 2.7, P*C*HCH_3), 29.04 (d, $^{1}J_{CP}$ = 24.0, P*C*HCH_3), 29.76 (d, J_{CP} = 25.2, P*C*HCH_3), 29.76 (dd, $^{1}J_{CP}$ = 29.2, $^{3}J_{CP}$ = 2.1, P*C*HCH_3), 36.98 (d, $^{2}J_{CP}$ = 27.2, CH_2), 66.85 (d, $^{2}J_{CP}$ = 15.8, *C*HCH_3), 125.32 (d, $^{2}J_{CP}$ = 1.8, *C*CH_3), 129.60 (d, J_{CP} = 4.0, CH), 129.75 (d, J_{CP} = 7.0, CH), 129.87 (d, J_{CP} = 6.5, CH), 130.00 (d, J_{CP} = 4.7, CH), 131.22 (d, J_{CP} = 2.2, CH), 131.65 (d, J_{CP} = 2.3, CH), 131.67 (d, $^{2}J_{CP}$ = 2.3, *C*CH_2), 132.24 (t, $^{2}J_{CP}$ = 1.1, *C*HCH_3), 132.40 (d, J_{CP} = 19.5, CH), 132.76 (d, J_{CP} = 19.9, CH), 139.55 (d, $^{1}J_{CP}$ = 56.5, C), 140.44 (d, $^{1}J_{CP}$ = 53.0, C), 153.04 (d, $^{2}J_{CP}$ = 41.0, C), 153.71 (d, $^{2}J_{CP}$ = 41.0, C). ^{29}Si{^{1}H} NMR (CD_2Cl_2): δ 24.12 (dd, J_{SiP} = 4.9, $J_{SiP'}$ = 2.2).

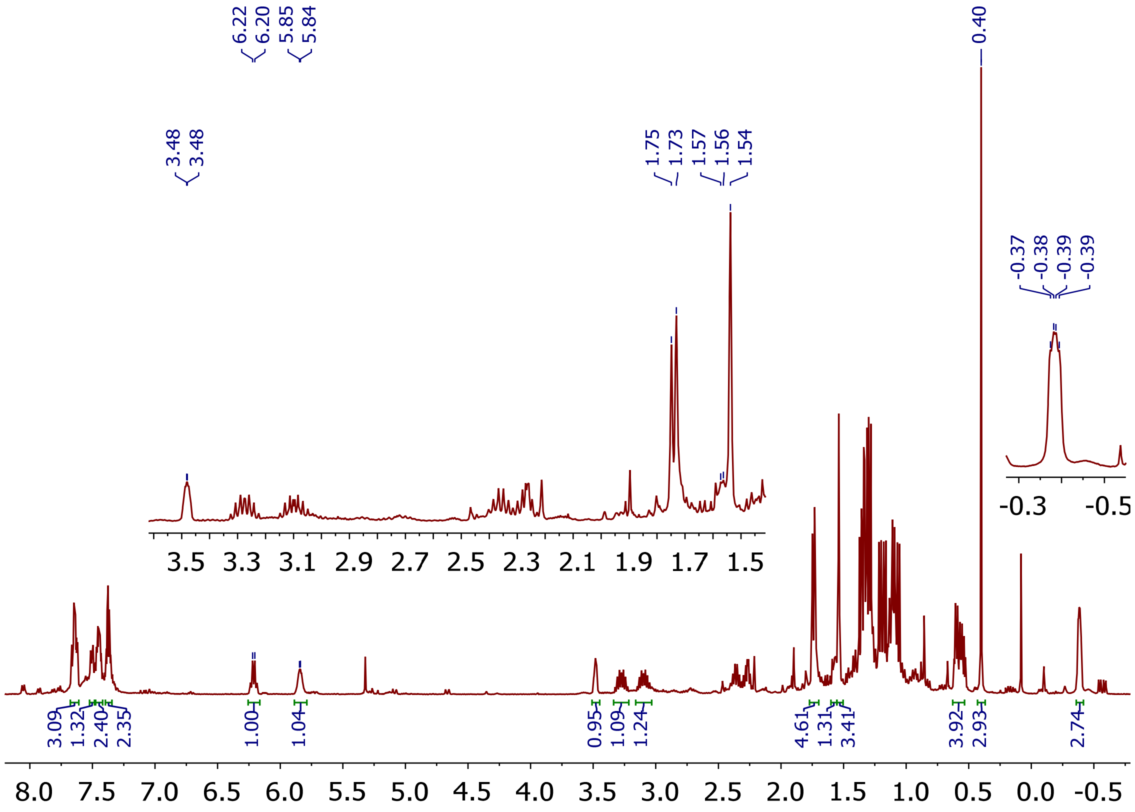

Figure S40. ^{1}H NMR spectrum of **7** in CD_2Cl_2 at 298 K.

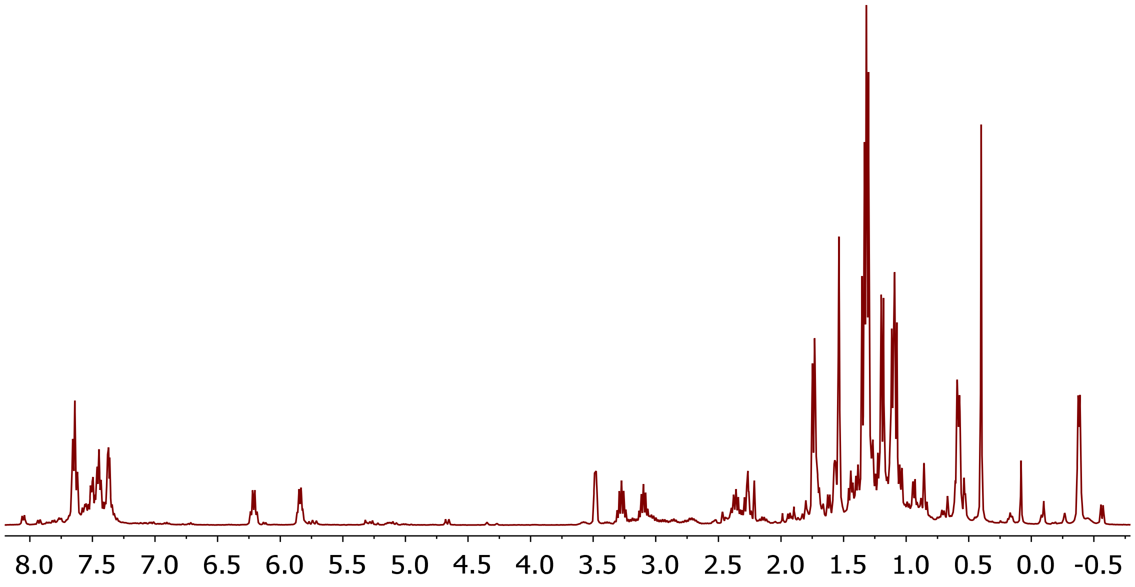

Figure S41. $^{1}H\{^{31}P\}$ NMR spectrum of **7** in CD_2Cl_2 at 298K.

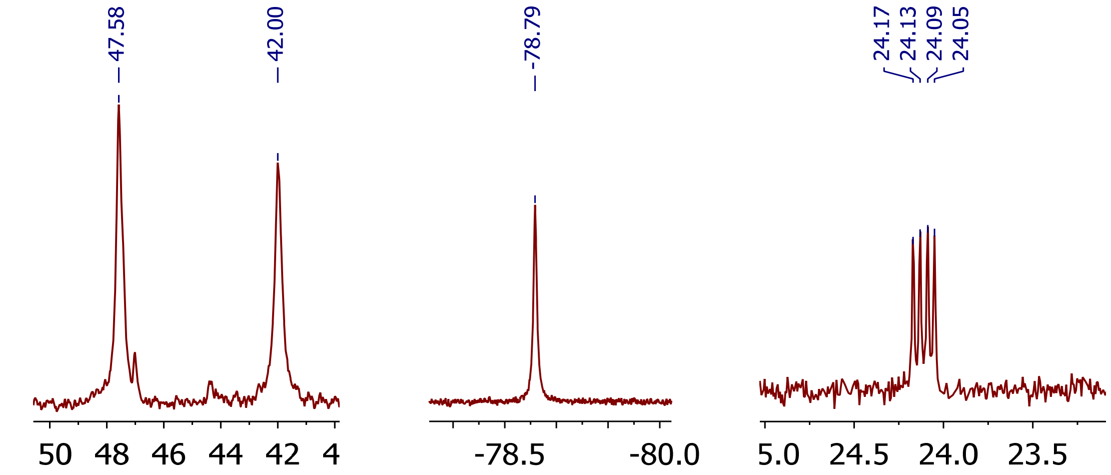

Figure S42. (left) $^{31}P\{^1H\}$, (center) ^{19}F, and (right) $^{29}Si\{^1H\}$ NMR spectra of **7** in CD_2Cl_2 at 298 K.

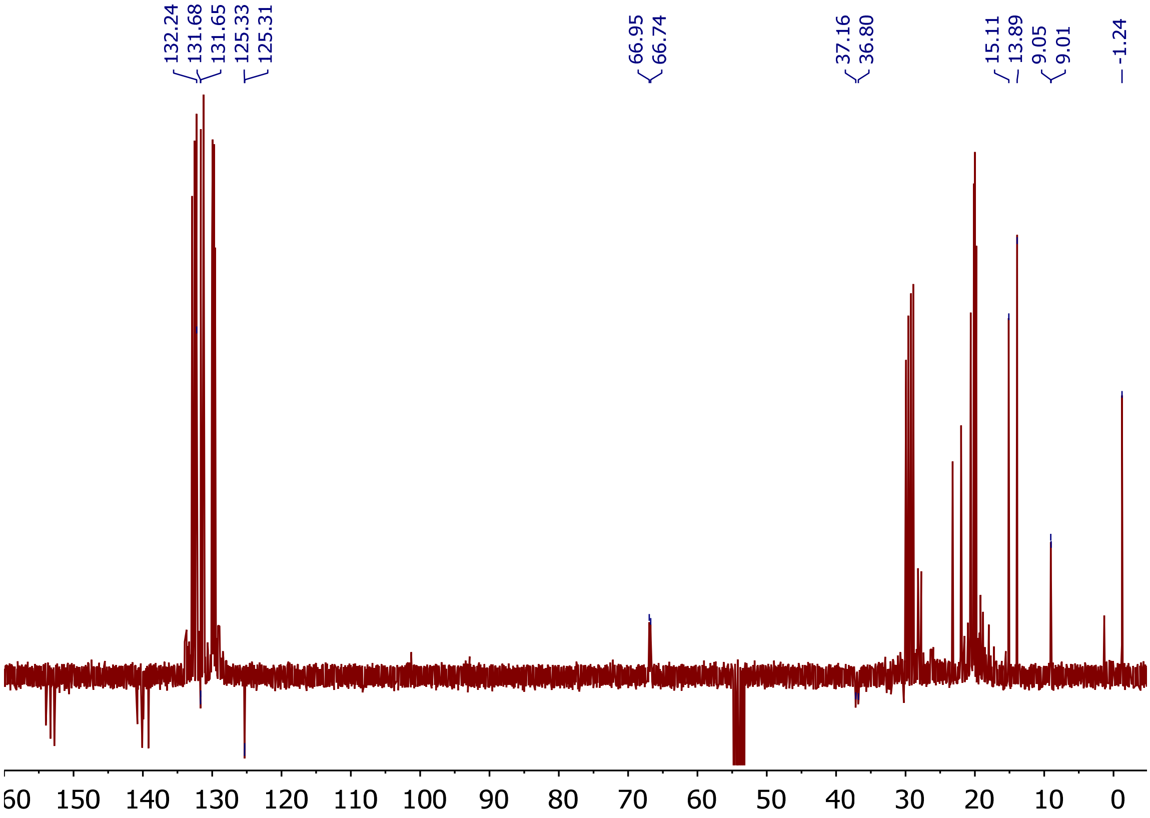

Figure S43. $^{13}C\{^1H\}$ APT NMR spectrum of **7** in CD_2Cl_2 at 298 K.

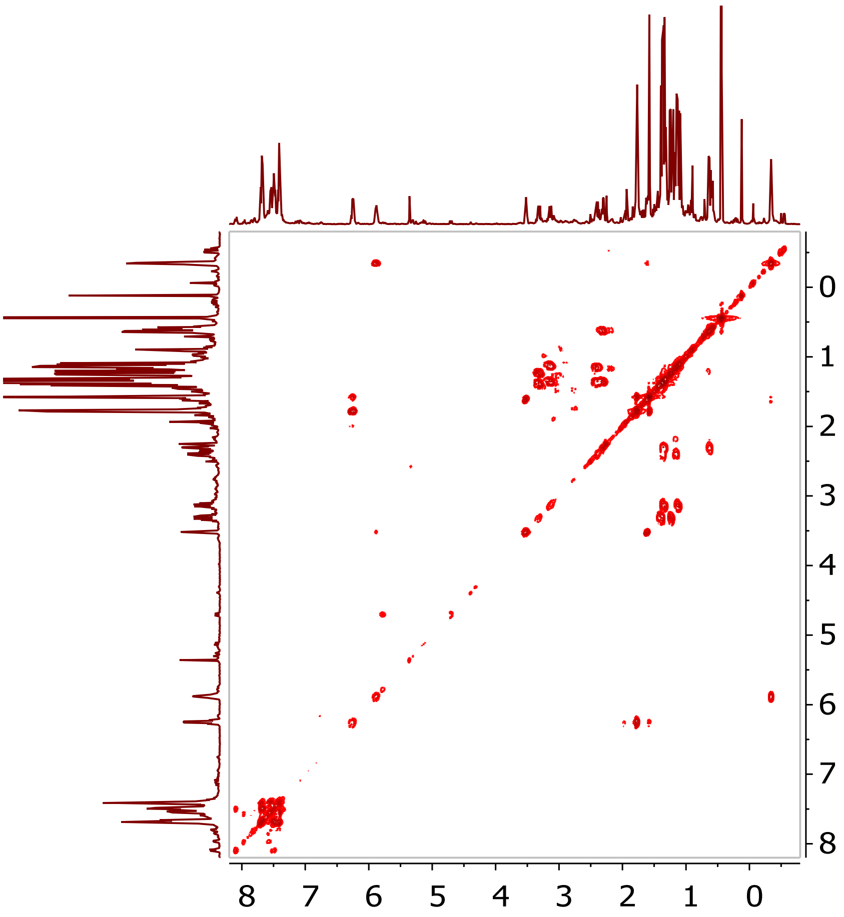

Figure S44. ^{1}H COSY NMR spectrum of **7** in CD_2Cl_2 at 298K.

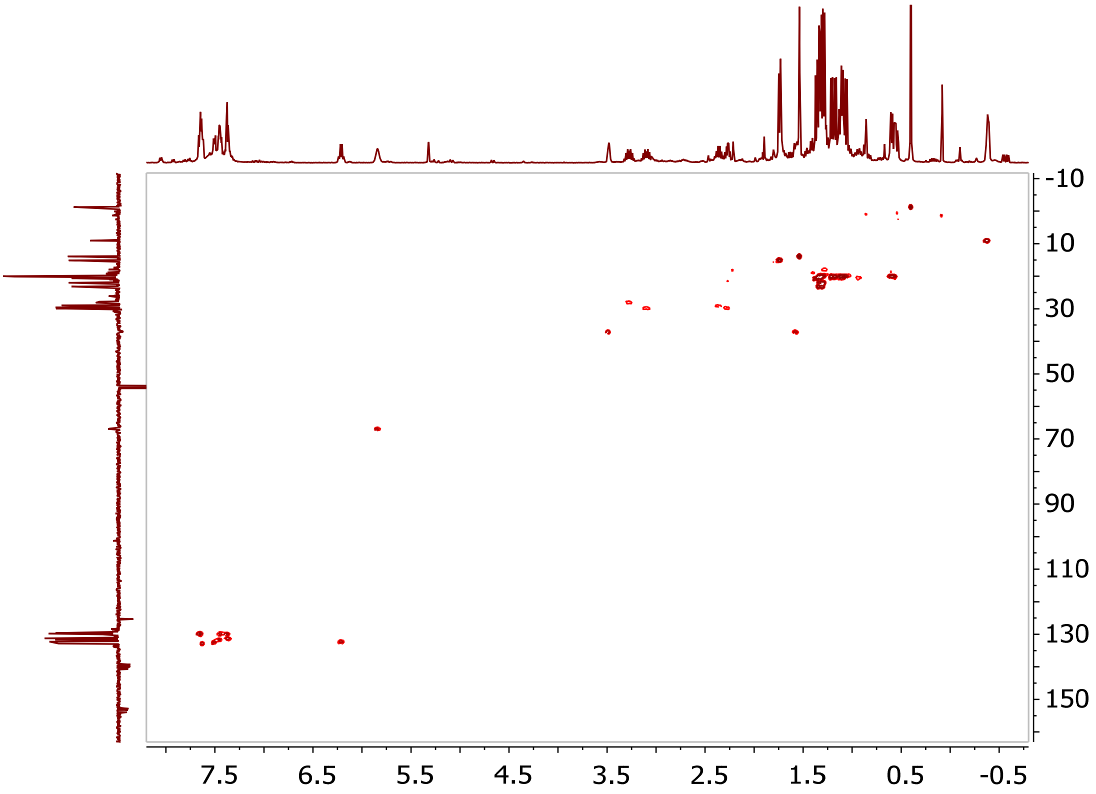

Figure S45. ^{1}H/^{13}C HSQC NMR spectrum of **7** in CD_2Cl_2 at 298 K.

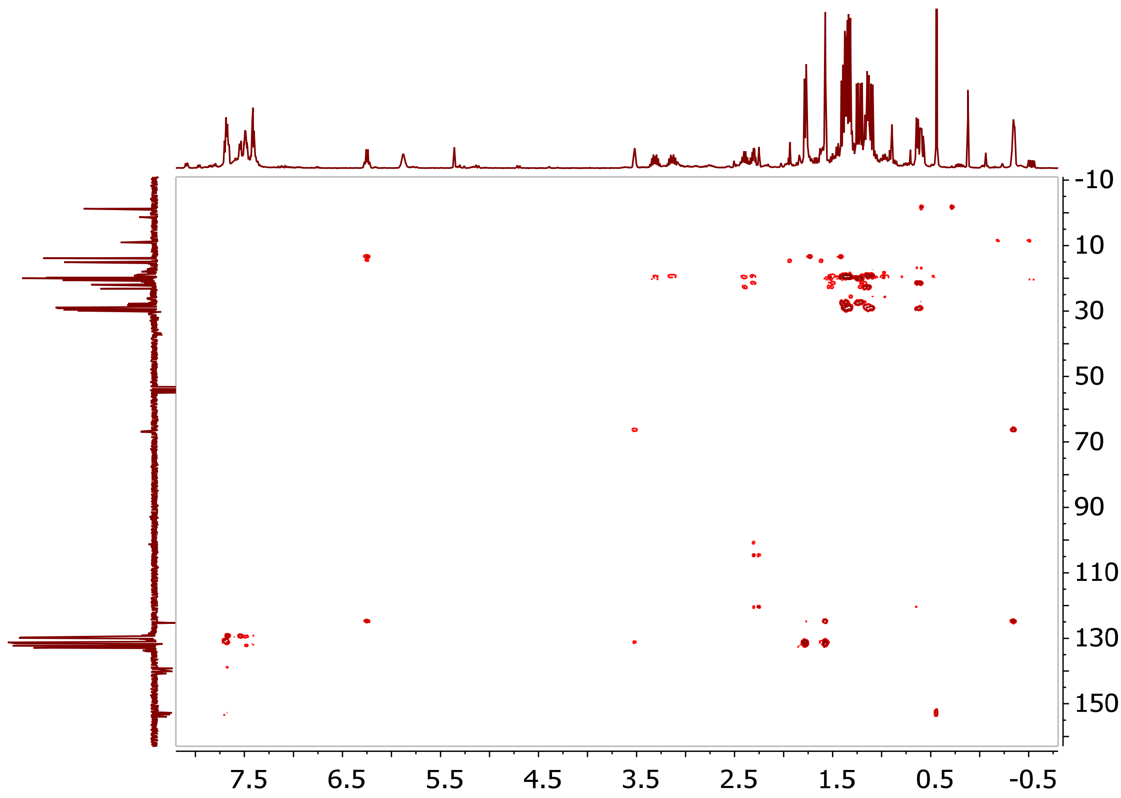

Figure S46. $^{1}H/^{13}C$ HMBC NMR spectrum of **7** in CD_2Cl_2 at 298 K.

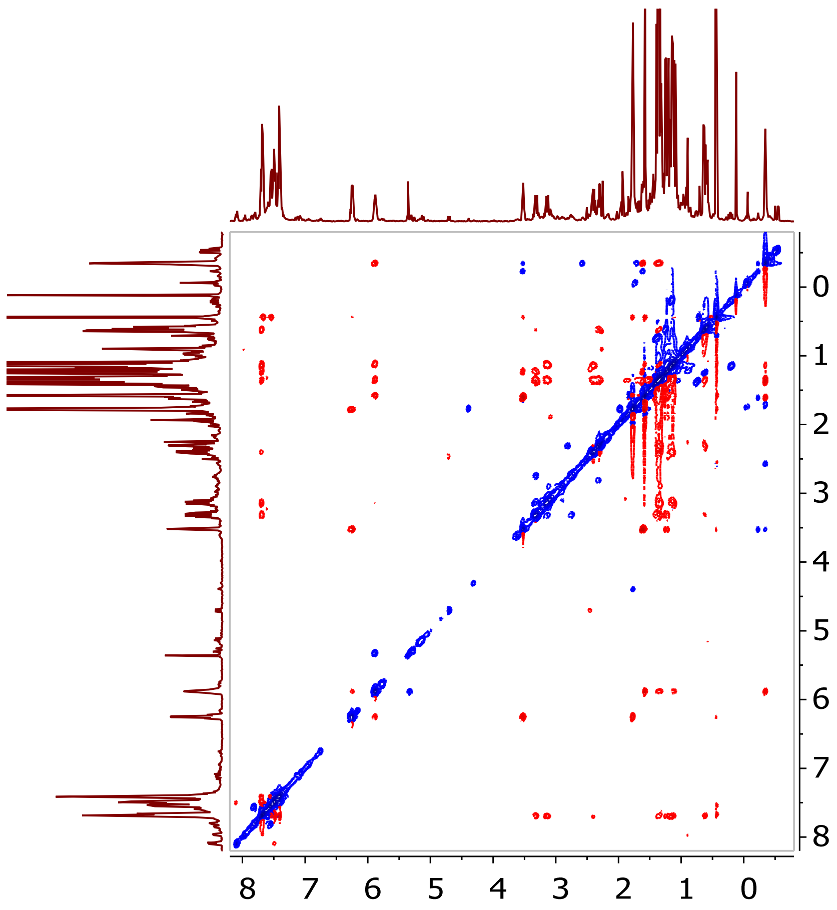

Figure S47. ^{1}H NOESY NMR spectrum of **7** in CD_2Cl_2 at 298 K.

Structural analysis of 2, [IrH{κP,P,Si-SiMe(C_6H_4-2-P*i*Pr$_2$)$_2$}($NCCH_3$)$_2$](BF_4) and 4:

X-ray data were collected at 100.0(2) K on Bruker SMART APEX CCD (**2, [IrH{κP,P,Si-Si(Me)(C_6H_4-2-P*i*Pr$_2$)$_2$}($NCCH_3$)$_2$](BF_4)**) and Smart APEX CCD DUO (**4**) diffractometers using graphite-monochromated Mo Kα radiation (λ = 0.71073 Å). Single crystals were mounted on a fiber and covered with protective perfluoropolyether. Data were collected over the complete sphere by a combination of six sets (**2, [IrH{κP,P,Si-Si(Me)(C_6H_4-2-P*i*Pr$_2$)$_2$}($NCCH_3$)$_2$](BF_4)**) or five sets (**4**). Data were corrected for absorption by using a multi-scan method applied with the SADABS program.[10] The structures were solved by the Patterson method and refined by full-matrix least squares on F^2 using the Bruker SHELXTL program package,[11] including isotropic and subsequently anisotropic displacement parameters for all non-hydrogen atoms. Weighted *R* factors (R_w) and goodness of fit (*S*) are based on F^2, and conventional *R* factors are based on *F*.

Data for 2. $C_{29}H_{46}P_2SiIr.CF_3SO_3$, *M* = 825.98; colourless irregular block, 0.09 x 0.07 x 0.07 mm^3; monoclinic, *P*21/c; *a* = 12.6431(9) Å, *b* = 13.6552(9) Å, *c* = 18.9344(13) Å; β = 95.0550(10); *Z* = 4; *V* = 3256.2(4) Å^3; D_c = 1.685 g/cm^3; μ = 4.347 mm^{-1}, minimum and maximum transmission factors 0.542 and 0.624; $2\theta_{max}$ = 57.50; 23257 reflections collected, 7721 unique [*R*(int) = 0.0849]; number of data/restrains/parameters 7721/0/400; final GoF 0.775, *R*1 = 0.0353 [5628 reflections $I > 2\sigma(I)$], *wR*2 = 0.0741 for all data; largest peak and hole 1.881 and −1.790 e Å^{-3}. Hydrogen atoms of the allyl ligand were observed in the difference Fourier maps and refined with the thermal parameter related to the bonded atoms. The rest of the hydrogen atoms were calculated and refined using a restricted riding model on their respective carbon atoms. The highest electronic residuals were observed in close proximity of the Ir center and make no chemical sense.

Data for [IrH{κP,P,Si-SiMe(C_6H_4-2-P*i*Pr$_2$)$_2$}($NCCH_3$)$_2$](BF_4). $C_{29}H_{46}N_2P_2SiIr.BF_4.C_2H_3N$, *M* = 832.79; colourless irregular block, 0.16 x 0.12 x 0.10 mm^3; monoclinic, *P*21/c; *a* = 18.0204(9) Å, *b* = 9.1341(5) Å, *c* = 22.0261(11) Å; β = 96.8920(10); *Z* = 4; *V* = 3599.3(3) Å^3; D_c = 1.537 g/cm^3; μ = 3.877 mm^{-1}, minimum and maximum transmission factors 0.406 and 0.499; $2\theta_{max}$ = 57.52; 62356 reflections collected, 8854 unique [*R*(int) = 0.0391]; number of data/restrains/parameters 8854/31/406; final GoF 1.109, *R*1 = 0.0246 [7767 reflections $I > 2\sigma(I)$], *wR*2 = 0.0584 for all data; largest peak and hole 1.246 (close to the Ir atom) and −0.959 e Å^{-3}. Hydrogen atoms (except the hydride) were calculated using a restricted riding model on their respective carbon atoms with the thermal parameter related to the bonded atom. The hydride was refined freely. The disordered BF_4 was refined with three moieties, complementary occupancy factors and restrained geometry. One crystallization molecule of acetonitrile was observed in the unit cell and refined freely.

Data for 4. $C_{25}H_{44}O_4P_2SiIr.CF_3SO_3.2(CH_2Cl_2)$, *M* = 1009.77; colourless irregular block, 0.30 x 0.16 x 0.12 mm^3; triclinic, *P*-1; *a* = 13.1361(13) Å, *b* = 13.5956(13) Å, *c* = 14.7124(14) Å; *a* = 89.1080(10); β = 63.6510(10); γ = 61.4270(10); *Z* = 2; *V* = 2002.1(3) Å^3; D_c = 1.675 g/cm^3; μ = 3.817 mm^{-1}, minimum and maximum transmission factors 0.254 and 0.362; $2\theta_{max}$ = 59.58; 25668 reflections collected, 10475 unique [*R*(int) = 0.0284]; number of data/restrains/parameters 10475/90/508; final GoF 1.058, *R*1 = 0.0322 [9688 reflections $I > 2\sigma(I)$], *wR*2 = 0.0824 for all data; largest peak and hole 5.178 and −1.327 e Å^{-3}. The OH and OH_2 hydrogen atoms were observed in the difference Fourier maps and refined with weak positional restrains and the thermal parameters related to the bonded oxygen atoms. The rest of the hydrogen atoms were calculated and refined using a restricted riding model on their respective carbon atoms. Two independent crystallization molecules of dichloromethane were observed in the unit cell, one of them was freely refined and the other one was refined with three moieties, complementary occupancy factors and restrained geometry. The highest electronic residuals were observed in close proximity of the Ir center and make no chemical sense.

Computational details

Calculations were carried out using the Gaussian09[12] and Gaussian16[13] program packages and an ONIOM-type approach. The first layer included all atoms except the four isopropyl substituents of the PSiP ligand for which a molecular mechanics force field (UFF) was used to estimate their steric effects. Optimizations and frequency calculations used the PBE1PBE[14] level of theory and the def2svp[15] basis set with its associated pseudopotential. Potential energies were further refined using the ωB97x-D[16] level of theory and the larger def2tzvp basis set with the corresponding ECP.[15] All the structures were optimized in solution using the default polarizable continuum method. Vibrational frequency calculations for all stationary points were carried out in order to assign their nature as minima (zero imaginary frequencies) or as transition state (one imaginary frequency). All the reported energies are free energies in solution at 298 K and 1 atm.

Table S2. Energies of the calculated compounds and transition states

	hartrees	hartrees	hartrees	cal mol^{-1} K^{-1}	hartrees	Kcal mol^{-1}	
	E_{el}	ZPE	H	S	G	ΔG	
**gas phase**							
2-butyne	-155.9812085	0.084160	0.089911	67.973	0.057614	0.00	
butadiene	-155.99390	0.085305	0.090913	67.500	0.058840	-7.20	
dendralene a	-312.0301403	0.174965	0.185488	94.308	0.140679	-26.53	
hexatriene b	-312.03110	0.175413	0.186037	94.915	0.140940	-26.96	
cyclohexadiene c	-312.06800	0.178415	0.187497	86.300	0.146493	-46.63	
triflate	-961.6785114	0.028111	0.036141	85.269	-0.004373		
triflic acid	-962.0701486	0.036025	0.043029	84.535	0.003807		
**1**$_{calc}$	-2543.337222	0.63489	0.677063	234.012	0.565876		
**[2**$_{calc}$**]**$^+$	-1737.522769	0.703261	0.739004	205.825	0.641210		0.00
**3**$_{calc}$	-2699.322698	0.727193	0.773278	258.439	0.650485		
**5**$_{calc}^{mer}$	-2699.304381	0.727864	0.773359	248.699	0.655194	0.00	
**[5**$_{cal}^{fac}$**]**$^+$	-1737.5070	0.698240	0.735423	209.646	0.635423	59.47	6.29
**[6**$_{calc}$**]**$^+$	-1893.570425	0.793312	0.833719	221.258	0.728592		-23.02
**[6'**$_{calc}$**]**$^+$	-1893.550475	0.791763	0.832924	226.590	0.725264		-12.59
**[7**$_{calc}$**]**$^+$	-1893.584282	0.792642	0.833367	225.458	0.726244	0.00	-33.19
**[7'**$_{calc}$**]**$^+$	-1893.559917	0.792577	0.833146	225.566	0.725972	15.12	-18.07
**[TS 2-8]**$^+$	-1737.493458	0.700096	0.734872	198.575	0.640523		17.96
**[8**$_{calc}$**]**$^+$	-1737.505544	0.702950	0.737792	198.617	0.643423		12.20
**[9**$_{calc}$**]**$^+$	-1893.558403	0.793289	0.833124	220.538	0.728339	17.55	-15.63
**benzene**							
triflate	-961.7555201	0.028099	0.036105	85.183	-0.004368		
**[5**$_{cal}^{fac}$**]**$^+$	-1737.535054	0.698325	0.735084	209.595	0.635499	0.00	
**5**$_{calc}^{mer}$	-2699.313513	0.727536	0.773131	249.448	0.654610	0.34	
**[TS 5**^{fac}**-10]**$^+$	-1737.515802	0.694286	0.731133	210.589	0.631075	9.30	
**[10**$_{calc}$**]**$^+$	-1737.543726	0.698033	0.735251	213.519	0.633801	-6.51	

**[TS 10-11^{syn}]$^{+}$**	-1737.504421	0.696553	0.733092	209.203	0.633693	18.09
**[TS 10-11^{anti}]$^{+}$**	-1737.505396	0.695556	0.732310	211.853	0.631652	16.20
**[11$_{calc}$^{syn}]$^{+}$**	-1737.5200	0.699292	0.735760	208.947	0.636482	10.03
**[11$_{calc}$^{anti}]$^{+}$**	-1737.529146	0.700732	0.736451	202.554	0.640211	6.66
**[TS 11^{syn}-2]$^{+}$**	-1737.507562	0.697032	0.733217	207.133	0.634801	16.81
**[TS 11^{anti}-2]$^{+}$**	-1737.510292	0.695089	0.732332	209.636	0.632728	13.80
**[2$_{calc}$]$^{+}$**	-1737.56479	0.704642	0.740058	200.742	0.644679	-12.90
**12$_{calc}$**	-2699.31478	0.728600	0.774265	254.650	0.653272	-1.63
**TS 12-13**	-2699.290344	0.723246	0.768882	258.178	0.646213	9.27
**13$_{calc}$**	-2699.3220	0.728146	0.773824	252.341	0.653929	-5.73
**TS 13-14**	-2699.286586	0.727294	0.771906	246.537	0.654768	17.00
**14$_{calc}$**	-2699.312056	0.727316	0.772944	255.775	0.651418	-1.09
**TS 14-15**	-2699.286067	0.722390	0.767223	253.137	0.646949	12.42
**15$_{calc}$**	-2699.334373	0.732244	0.777180	252.022	0.657436	-11.32
**16$_{calc}$**	-2699.3450	0.734189	0.777880	240.255	0.663728	-14.06
**TS 16-17**	-2699.285478	0.725018	0.769348	247.196	0.651897	15.89
**17$_{calc}$**	-2699.298942	0.728869	0.774105	256.324	0.652317	7.70
**TS 13-18**	-2699.298986	0.731800	0.775419	240.675	0.661067	13.17
**18**	-2699.304473	0.731941	0.77613	243.829	0.660281	9.23
**TS 18-3**	-2699.28599	0.732591	0.776719	246.431	0.659632	20.42
**19$_{calc}$**	-2699.314427	0.729830	0.774854	252.499	0.654883	-0.40
**TS 19-3**	-2699.304704	0.726450	0.772155	257.063	0.650017	2.65
**3$_{calc}$**	-2699.326615	0.726556	0.772862	257.539	0.650497	-10.80
**dichloromethane**						
triflate	-961.75552	0.027996	0.036020	85.333	-0.00452	
**[5$_{cal}$^{fac}]$^{+}$**	-1737.551917	0.697956	0.734821	210.670	0.634725	0.00
**5$_{calc}$^{mer}**	-2699.323596	0.727150	0.772839	250.140	0.653989	4.79
**TS [5^{fac}]$^{+}$ - [10]$^{+}$**	-1737.531786	0.693704	0.730700	209.951	0.630946	10.26
**[10$_{calc}$]$^{+}$**	-1737.565338	0.699933	0.736850	210.492	0.636838	-7.10
**TS [10]$^{+}$ - [11^{syn}]$^{+}$**	-1737.521554	0.695848	0.732436	209.648	0.632825	17.86
**[11$_{calc}$^{syn}]$^{+}$**	-1737.540243	0.700152	0.736394	207.384	0.637859	9.29
**TS [11^{syn}]$^{+}$ - [2]$^{+}$**	-1737.524563	0.696583	0.732775	207.227	0.634315	16.91
**[2$_{calc}$]$^{+}$**	-1737.58585	0.705201	0.740412	199.214	0.645760	-14.37
**16$_{calc}$**	-2699.354374	0.733953	0.777733	240.960	0.663245	-8.72
**TS 16-17**	-2699.289604	0.768257	0.769201	248.165	0.651290	24.42
**17$_{calc}$**	-2699.303812	0.726910	0.773838	255.616	0.652386	16.20

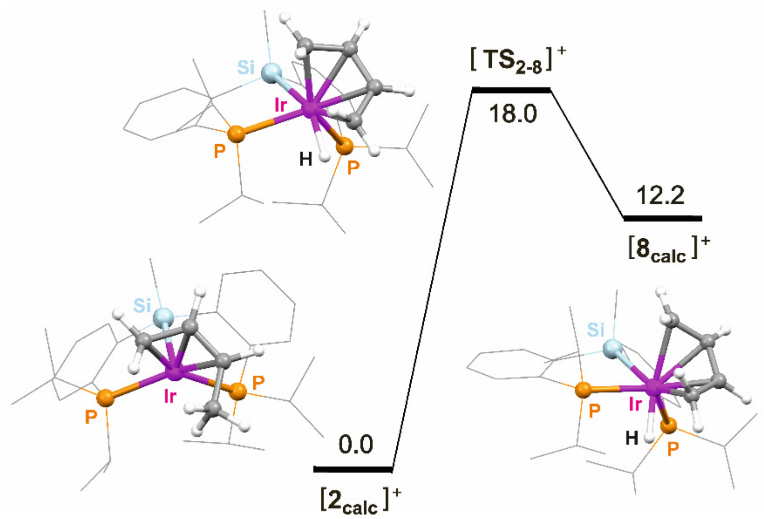

Figure S48. Transition state **[TS$_{2\text{-}8}$]**$^+$ and hydrido-butadiene intermediate **[8$_{calc}$]**$^+$ calculated for the intraligand H-atom exchange in cationic complex **2**.

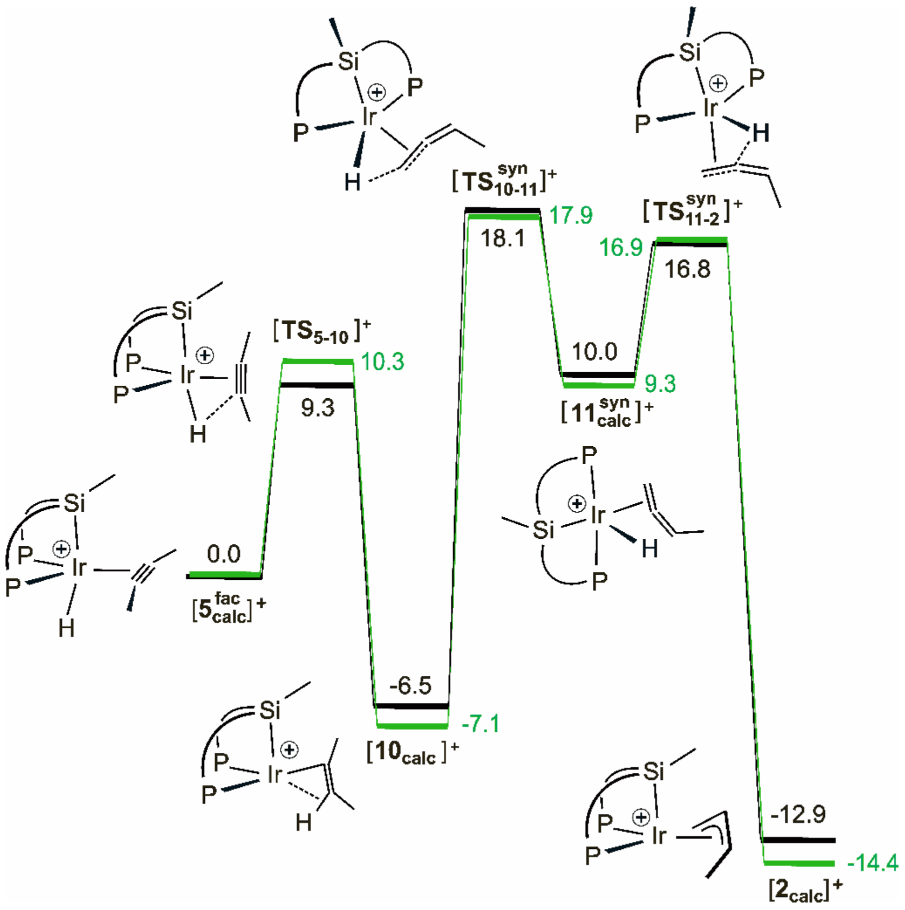

Figure S49. Comparison between calculated trajectories for the Green's mechanism in benzene (black) and dichloromethane (green). Only the syn intermediates are shown for the sake of clarity.

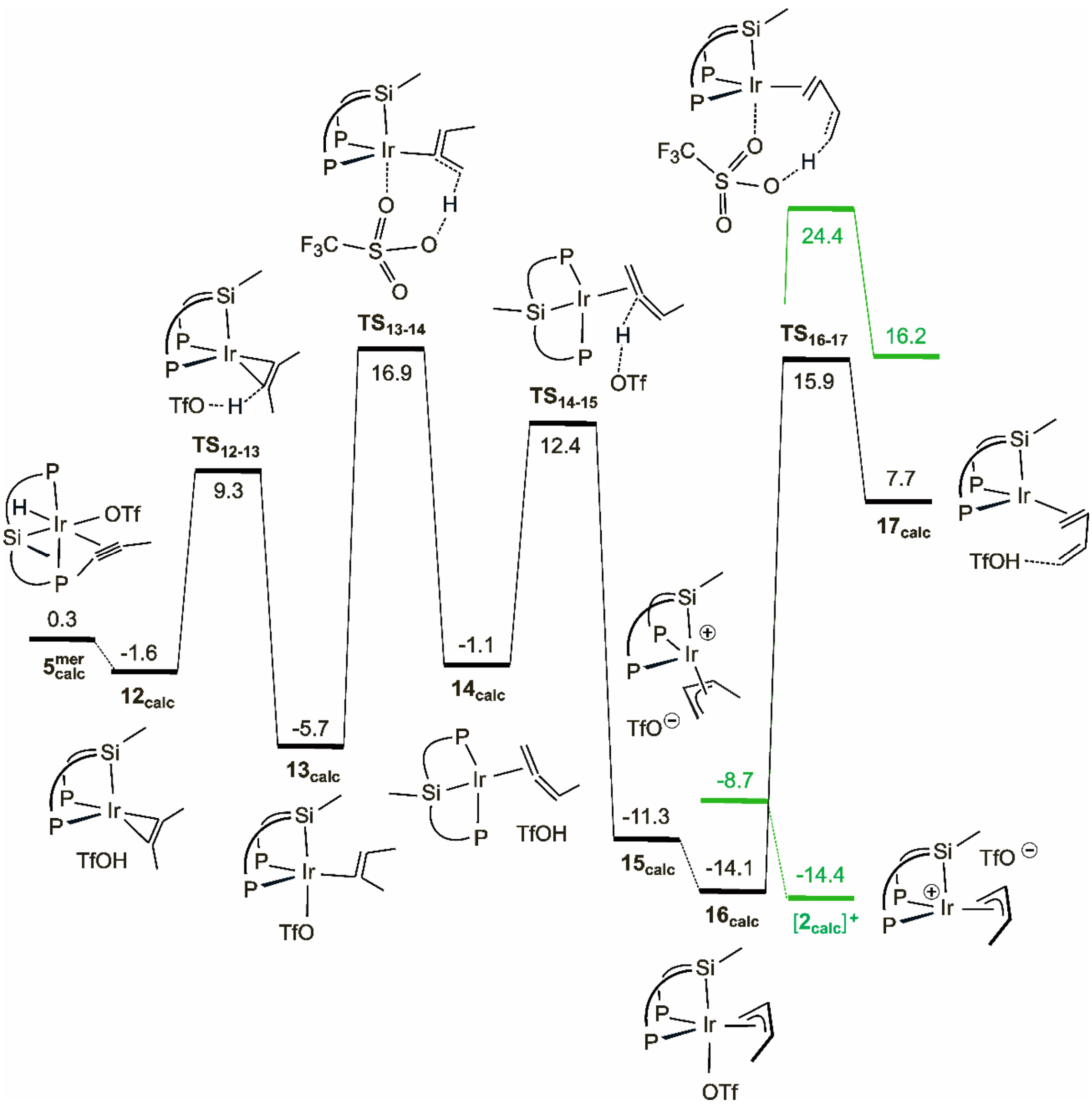

Figure S50. Comparison between calculated trajectories for butadiene release in benzene (black) and dichloromethane (green).

References

1 Suárez, E.; Plou, P.; Gusev, D. G.; Martín, M.; Sola, E. Cationic, Neutral, and Anionic Hydrides of Iridium with PSiP Pincers. *Inorg. Chem.* **2017**, *56*, 7190–7199.

2 Fang, H.; Choe, Y. K.; Li, Y.; Shimada, S. Synthesis, Structure, and Reactivity of Hydridoiridium Complexes Bearing a Pincer-Type PSiP Ligand. *Chem., Asian J.* **2011**, *6*, 2512–2521.

3 Gajewski, J. J.; Shih, C. N. Characterization of the Dimethyl-l,2-Dimethylenecyclobutanes from the Methylallene Thermal Dimerization. *J. Org. Chem.* **1972**, *37*, 64–68.

4 Doering, W. V. E.; Roth, W. R.; Bauer, F.; Boenke, M.; Breuckmann, R.; Ruhkamp, J.; Wortmann, O. Rotationsbarrieren Vinyl-substituierter Olefine. *Chem. Ber.* **1991**, *124*, 1461–1470.

5 Becker, K. B. Preparation of 2,3-Dialkylcyclohexa-l,3-Dienes by the Bis-Wittig Reaction. *Synth.* **1980**, *1980* (3), 238–240.

6 Karunakaran, C.; Santharaman, P.; Balamurugan, M. 1H and 13C Nuclear Magnetic Resonance Spectroscopy. In *Spin Resonance Spectroscopy: Principles and applications*; Karunakaran, C. B. T.-S. R. S., Ed.; Elsevier, 2018; pp 49–110.

7 Faller, J. W. Spin Saturation Labeling. In *Determination of Organic Structures by Physical Methods*; Elsevier, 1973; Vol. 5, pp 75–97. https://doi.org/10.1016/b978-0-12-513405-7.50008-5.

8 Morse, P. M.; Spencer, M. D.; Wilson, S. R.; Girolami, G. S. A Static Agostic α-CH···M Interaction Observable by NMR Spectroscopy: Synthesis of the Chromium(II) Alkyl [Cr2(CH2SiMe3)6]2− and Its Conversion to the Unusual "Windowpane" Bis(Metallacycle) Complex [Cr(K2C,C′-CH2SiMe2CH2)2]2−. *Organometallics* **1994**, *13* (5), 1646–1655.

9 Taylor, J. R. *An Introduction to Error Analysis*, 2nd ed.; University Science Books: Mill valley, CA, 1982

10 Blessing, R. H. Acta Crystallogr., Sect. A: Found. Crystallogr. 1995, 51, 33. SADABS: Area-detector absorption correction; Bruker-AXS, Madison, WI, 1996.

11 SHELXTL Package v. 6.14; Bruker-AXS, M., WI, 2000. Sheldrick, G. M. A Short History of SHELX. Acta Crystallogr., Sect. A: Found. Crystallogr., 2008, 64, 112–122.

12 Frisch, M. J.; Trucks, G. W.; Schlegel, H. B.; Scuseria, G. E.; Robb, M. A.; Cheeseman, J. R.; Scalmani, G.; Barone, V.; Mennucci, B.; Petersson, G. A.; Nakatsuji, H.; Caricato, M.; Li, X.; Hratchian, H. P.; Izmaylov, A. F.; Bloino, J.; Zheng, G.; Sonnenberg, J. L.; Hada, M.; Ehara, M.; Toyota, K.; Fukuda, R.; Hasegawa, J.; Ishida, M.; Nakajima, T.; Honda, Y.; Kitao, O.; Nakai, H.; Vreven, T.; Peralta, J. E., Jr; Ogliaro, F.; Bearpark, M.; Heyd, J. J.; Brothers, E.; Kudin, K. N.; Staroverov, V. N.; Kobayashi, R.; Normand, J.; Raghavachari, K.; Rendell, A.; Burant, J. C.; Iyengar, S. S.; Tomasi, J.; Cossi, M.; Rega, N.; Millam, J. M.; Klene, M.; Knox, J. E.; Cross, J. B.; Bakken, V.; Adamo, C.; Jaramillo, J.; Gomperts, R.; Stratmann, R. E.; Yazyev, O.; Austin, A. J.; Cammi, R.; Pomelli, C.; Ochterski, J. W.; Martin, R. L.; Morokuma, K.; Zakrzewski, V. G.; Voth, G. A.; Salvador, P.; Dannenberg, J. J.; Dapprich, S.; Daniels, A. D.; Farkas; Foresman, J. B.; Ortiz, J. V.; Cioslowski, J.; Fox, D. J. Gaussian 09, revision D.01; Gaussian Inc.: Wallingford, CT, 2013.

13 Frisch, M. J.; Trucks, G. W.; Schlegel, H. B.; Scuseria, G. E.; Robb, M. A.; Cheeseman, J. R.; Scalmani, G.; Barone, V.; Petersson, G. A.; Nakatsuji, H.; Li, X.; Caricato, M.; Marenich, A. V.; Bloino, J.; Janesko, B. G.; Gomperts, R.; Mennucci, B.; Hratchian, H. P.; Ortiz, J. V.; Izmaylov, A. F.; Sonnenberg, J. L.; Williams-Young, D.; Ding, F.; Lipparini, F.; Egidi, F.; Goings, J.; Peng, B.; Petrone, A.; Henderson, T.; Ranasinghe, D.; Zakrzewski, V. G.; Gao, J.; Rega, N.; Zheng, G.; Liang, W.; Hada, M.; Ehara, M.; Toyota, K.; Fukuda, R.; Hasegawa, J.; Ishida, M.; Nakajima, T.; Honda, Y.; Kitao, O.; Nakai, H.; Vreven, T.; Throssell, K.; Montgomery, J. A., Jr.; Peralta, J. E.; Ogliaro, F.; Bearpark, M. J.; Heyd, J. J.; Brothers, E. N.; Kudin, K. N.; Staroverov, V. N.; Keith, T. A.; Kobayashi, R.; Normand, J.; Raghavachari, K.; Rendell, A. P.; Burant, J. C.; Iyengar, S. S.; Tomasi, J.; Cossi, M.; Millam, J. M.; Klene, M.; Adamo, C.; Cammi, R.; Ochterski, J. W.; Martin, R. L.; Morokuma, K.; Farkas, O.; Foresman, J. B.; Fox, D. J. Gaussian 16, revision A.03; Gaussian, Inc.: Wallingford, CT, 2016.

14 Adamo, C.; Barone, V. Toward reliable density functional methods without adjustable parameters: The PBE0 model. *J. Chem. Phys.* **1999**, *110*, 6158−6170.

15 Weigend, F.; Ahlrichs, R. Accurate Coulomb-fitting basis sets for H to Rn. *Phys. Chem. Chem. Phys*. **2005**, *7*, 3297−3305.

16 Chai, J.-D.; Head-Gordon, M. Long-range corrected hybrid density functionals with damped atom-atom dispersion corrections. *Phys. Chem. Chem. Phys.* 2008, *10*, 6615–6620.
